# Novel track morphotypes from new tracksites indicate increased Middle Jurassic dinosaur diversity on the Isle of Skye, Scotland

**DOI:** 10.1371/journal.pone.0229640

**Published:** 2020-03-11

**Authors:** Paige E. dePolo, Stephen L. Brusatte, Thomas J. Challands, Davide Foffa, Mark Wilkinson, Neil D. L. Clark, Jon Hoad, Paulo Victor Luiz Gomes da Costa Pereira, Dugald A. Ross, Thomas J. Wade

**Affiliations:** 1 School of GeoSciences, University of Edinburgh, Edinburgh, Scotland, United Kingdom; 2 National Museums Scotland, Edinburgh, Scotland, United Kingdom; 3 The Hunterian, University of Glasgow, Glasgow, Scotland, United Kingdom; 4 Art of Ancient Life Limited, Perth, Scotland, United Kingdom; 5 Departamento de Geologia, Universidade Federal do Rio de Janeiro, Rio de Janeiro, Brazil; 6 Staffin Museum, Staffin, Isle of Skye, Scotland, United Kingdom; Perot Museum of Nature and Science, UNITED STATES

## Abstract

Dinosaur fossils from the Middle Jurassic are rare globally, but the Isle of Skye (Scotland, UK) preserves a varied dinosaur record of abundant trace fossils and rare body fossils from this time. Here we describe two new tracksites from Rubha nam Brathairean (Brothers’ Point) near where the first dinosaur footprint in Scotland was found in the 1980s. These sites were formed in subaerially exposed mudstones of the Lealt Shale Formation of the Great Estuarine Group and record a dynamic, subtropical, coastal margin. These tracksites preserve a wide variety of dinosaur track types, including a novel morphotype for Skye: *Deltapodus* which has a probable stegosaur trackmaker. Additionally, a wide variety of tridactyl tracks shows evidence of multiple theropods of different sizes and possibly hints at the presence of large-bodied ornithopods. Overall, the new tracksites show the dinosaur fauna of Skye is more diverse than previously recognized and give insight into the early evolution of major dinosaur groups whose Middle Jurassic body fossil records are currently sparse.

## Introduction

Dinosaur fossils are sparse from the Middle Jurassic (174–164 Ma) [1]. However, the fossiliferous Great Estuarine Group which crops out in the Hebrides of Scotland, UK, spans this time period [2]. Both body and trace fossils found in the Hebrides, particularly on the Isle of Skye, have the potential to fill in gaps in our understanding of dinosaurs during this critical time in their evolution when sauropods, theropods, and thyreophorans all experienced evolutionary radiations [3–7].

The Great Estuarine Group of Skye has yielded isolated dinosaur body fossils including sauropod teeth and limb bones, theropod teeth and vertebrae, and a thyreophoran ulna and radius [8–14]. Skye preserves evidence of vibrant terrestrial and marine ecosystems that, in addition to dinosaurs, included sharks, ichthyosaurs, plesiosaurs, salamanders, turtles, crocodylomorphs, tritylodonts, and docodonts [15–20].

By far, however, the most common dinosaur fossils on Skye are tracks. They are often found on intertidal platforms, primarily along the northern coast of the island [21 and references therein]. The first Scottish dinosaur fossil reported was a single isolated footprint (GLAHM V1980), which had fallen from the cliffs at Rubha nam Brathairean (Brothers’ Point), on the Trotternish Peninsula of Skye [22]. Since that time, several other tracksites have been discovered across the island, including well-known trackways at Staffin [23] and Duntulm [24] that are visible *in-situ* and have become popular tourist attractions. Most recently, our team has described a new site with sauropod and theropod tracks from Rubha nam Brathairean (the ‘Brothers’ Point 2’ site), geographically close to where the first isolated track was found, but likely from a different stratigraphic horizon [25].

Here we describe two new dinosaur tracksites from Rubha nam Brathairean (hereafter called by its English name, Brothers’ Point). These tracksites preserve a novel track morphotype of a probable stegosaur that was hitherto unknown from Skye and which also represents one of the oldest fossil records of this major dinosaur group from anywhere in the world. These new tracksites complement the sparse dinosaur body fossil record of Skye and further illustrate the high diversity of dinosaurs on the island during the Middle Jurassic.

## Discovery of the tracksites

The two tracksites we describe here are referred to as Brothers’ Point 1 (BP1) and Brothers’ Point 3 (BP3). As mentioned above, another tracksite from the same area, denoted as Brothers’ Point 2, which preserves sauropod and theropod tracks made in a shallow lagoon, was described separately in [25]. All three tracksites were discovered during fieldwork on the Isle of Skye by the PalAlba consortium of Scottish-based paleontologists. The numbering scheme refers to the order in which the three sites were found, over the course of fieldwork from 2015–2017.

BP1 was discovered in the autumn of 2015 by Thomas Challands while prospecting with Neil Clark. BP3 was discovered in May 2017, by Paulo Pereira, while prospecting as part of a large PalAlba fieldtrip funded by the National Geographic Society. Both sites were subsequently mapped and studied in detail by Paige dePolo as part of her MScR. thesis at the University of Edinburgh [26].

## Geological context

The Inner Hebrides, an island archipelago off the west coast of Scotland, feature one of the most complete sequences of Middle Jurassic sedimentary rocks in the world [27]. A series of formations, known collectively as the Great Estuarine Group, record repeated cycles of delta progradation and retrogradation into marine-influenced lagoons, during the Bajocian-Bathonian (ca. 170–166 million years ago) [28]. The dating of these rocks has proved challenging historically because they do not preserve the correct depositional environments for ammonites, which are the most relevant index fossils [29]. However, their stratigraphic relationships with the underlying Bajocian Garantiana Clay Member and the Bearreraig Sandstone Formation and the overlying Callovian Staffin Bay Formation constrain the age range for these rocks to latest Bajocian to Bathonian [2, 30–32]. The best exposures of the Great Estuarine Group are on the Isle of Skye, the largest island of the Inner Hebrides. Both new tracksites (BP1 and BP3) are located at Brothers’ Point on the Trotternish Peninsula and occur in the Lonfearn Member of the Lealt Shale Formation (Great Estuarine Group).

**Fig 1:**
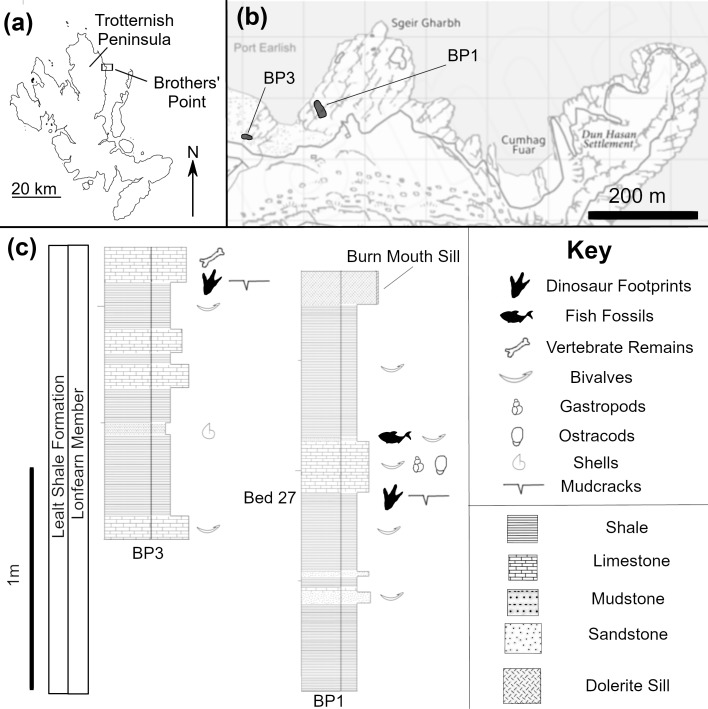
Geographical and geological context of BP1 and BP3. The geographical location of the sites on (a) the Isle of Skye and (b) the Trotternish Peninsula and (c) the bedding-scale stratigraphy of the Lonfearn Member, Lealt Shale Formation around each site. Bed 27 marks the inferred stratigraphic layer of the first dinosaur footprint from Scotland [22] according to the numbering scheme from [2]. It is located at or near the level of the track-bearing layer at BP1. Maps adapted from BGS 1:50 000 [Shapefile geospatial data], scale 1:50 000, tile: SC0803, version 2016, British Geological Survey, UK, using: EDINA Geology Digimap Service http://digimap.edina.ac.uk, downloaded October 2017, © Geological Map Data BGS © NERC 2017.

BP1 is located on an inter-tidal platform east of the mouth of Lonfearn Burn and immediately adjacent to the large sill that forms the western edge of Sgeir Gharbh (57.5865° N, 6.1472° W; Fig 1B). This sill is referred to as the Burn Mouth Sill (John Hudson, personal communication). The walking path to Brothers’ Point passes along the cliff face to the south of the outcrop and a small stream branches off from Lonfearn Burn and flows along the western side of the platform. BP1 is regularly exposed during low tide, although it is frequently covered by seaweed.

BP3 is located immediately west of the mouth of Lonfearn Burn into Port Earlish and south of (shoreward from) a large volcanic intrusion (57.5863°N, 6.1494°W; Fig 1B). The site is found among the large, wave-washed boulders of the coast. Since there was very little growth of seaweed and other coastal life on the exposed surfaces at the time of its discovery in 2017, BP3 likely was covered by boulders until soon before its discovery. Thus, in the future, BP3 may be particularly liable to becoming re-covered during storms.

BP1 and BP3 are located in the upper part of the Lonfearn Member, within the transitional facies between a lagoon-dominated depositional environment and the overlying deltaic sandstones of the Valtos Formation [2].

### Brothers’ point 1

The tracks at BP1 are preserved as impressions (concave epirelief) in a dark gray, thinly- laminated, fine-grained calcareous shale overlain and in-filled by a light tan, bioclastic limestone. Upon close examination, the track-bearing horizon is composed predominantly of clay to silt-sized carbonate mud with intermittent, weakly defined layers of broken bivalve shells. Moderate pyritization occurs around the outer edges of some of the larger shells in the section.

The shale of the track-bearing layer contains extensive mudcracks that often propagate from the toes of the tridactyl tracks. This association of mudcracks and dinosaur tracks indicates that both features were formed near the same time when the sediments in question were exposed subaerially. Fig 1C shows a stratigraphic section of BP1 through the beds immediately above and below the track-bearing layer. The section is dominated by shales with isolated beds of coarser shelly limestones and sandstones. The track-bearing layer marks the only definitive subaerial exposure in this short (1.90 m) section that was logged at the cm-scale. In some beds below the track-bearing layer, broken bivalve shells are visible to the naked eye and lend support to the inference that the area was generally subaqueous. Additionally, the lithology immediately above the track-bearing layer indicates the subaerial exposure observed at the site was rather transient. In particular, the presence of a thin bone bed comprised of broken fish material immediately above the bioclastic limestone that infills the tracks provides strong evidence that the site was resubmerged after the desiccation event in which the mudcracks formed.

The limestone unit overlying the track-bearing layer is composed almost exclusively of densely packed shells of the bivalve *Neomiodon* (John Hudson, personal communication). Monotypic shells beds such as these are characteristic of the upper portions of the Lealt Shale Formation’s Lonfearn Member [2, 33]. In addition to *Neomiodon* as the dominant invertebrate, isolated smooth-shelled ostracods, possibly *Alicenula phaselus*, are present (Matt Wakefield, personal communication). Very rarely, broken gastropods are visible in thin section.

One of the characteristics of a brackish-water faunal assemblage is a low variety of species and a large number of individuals [33]. The assemblage of predominantly *Neomiodon*, with lower concentrations of *Viviparus*, ostracods and conchostracans (known in older literature as *Estheria*, *Euestheria*, *Cyzicus*, and spinicaudatans) is indicative of a low-salinity environment [33]. This faunal assemblage indicates that the track-bearing bed and adjacent layers are located in the transitional zone between the more brackish Lealt Shale Formation and the freshwater Valtos Formation [34].

We reconstruct the depositional environment for BP1 as a briefly exposed, subaerial mudflat adjacent to brackish (but tending towards freshwater) lagoons. While the dinosaur tracks were made upon a desiccating surface, this time of exposure was, likely, relatively brief in terms of geologic time and, as evidenced by the presence of fish bones in the beds immediately overlying the desiccation surface, the spot was quickly reclaimed by water. The overall environment reconstructed for this area is a dynamic, rapidly fluctuating, coastal margin.

### Brothers’ point 3

The tracks at BP3 are preserved as impressions (concave epirelief) in a shale overlain and in-filled by a well-sorted, bioclastic limestone (Fig 1C). The shale is medium to dark blue-gray, micritic, and dominated by thin (< 5 mm) laminations. Thin lenses (< 1 mm thick) of a paler colored fine-grained sand are sparsely interspersed between the shale layers. The overlying bioclastic limestone is predominantly composed of disarticulated to broken shell fragments of *Neomiodon* with mm-scale lenses of dark-colored silt. Large (3–4 mm), subhedral pyrite crystals are present in the bioclastic limestone. They are probably of diagenetic origin [35].

The invertebrate fauna at BP3 is dominated by *Neomiodon*. However, the shell beds are not as densely packed as those at BP1 and mud separates the disarticulated valves. Other invertebrates present at the site include ostracods and conchostracans. Conchostracans are common in the Lealt Shale Formation but become rarer with increasing stratigraphic height within the formation and through the associated facies shift to the Valtos Formation which contains few examples of these invertebrates [2, 36]. Their abundance in the track-bearing mudstone, in particular, is reasonable evidence that the bed in question is still part of the Lealt Shale Formation.

The dinosaur tracks at BP3 occur in a single, track-bearing layer with localized soft-sediment deformation associated with tracks disrupting the underlying thin shaley laminations. Desiccation cracks are not visible along the southern portion of the platform where the largest concentration of tracks is located. However, these sedimentary structures are visible on the same bedding surface about 5 m to the north and are associated with two additional tracks. The variable association of footprint and desiccation crack occurrences can arise due to variations in surface saturation or in the rates at which different portions of the surface dried. Due to the presence of desiccation cracks and the undeformed nature of the overlying bioclastic limestone that is infilling the tracks, it is likely that the currently exposed surface was close to the original surface on which the dinosaurs walked and that it was subaerially exposed near the time of track formation.

While the dinosaur tracks at BP3 were being described, an articulated pterosaur skeleton (illustrated as ‘vertebrate remains’ in Fig 1C) was found in the overlying limestone layer by Amelia Penny. The unfractured nature of the delicate bones (many of which are hollow) and overall completeness of the skeleton indicates that the overlying limestone was deposited in a relatively low-energy environment. This specimen is currently under study and will be fully described in a subsequent PalAlba publication. The inferred energy setting of the overlying limestone indicates that, when the track-bearing surface was resubmerged, the influence of currents, tides, and other potential mechanisms for reworking material was relatively minimal. Thus, the subsequent environment supported the preservation of these tracks.

We interpret the depositional environment for BP3, like at BP1, as a briefly exposed, subaerial mudflat where the dinosaur tracks were formed on a desiccating surface. The area was quickly reclaimed by the adjacent, low-energy, brackish to freshwater lagoons. The depositional environment for BP3 corresponds to that determined for BP1 –a rapidly changing coastal margin.

### Stratigraphic context of the tracksites

Several detailed stratigraphic sections for the Lealt Shale Formation have been published [2,29] and the locations of BP1 and BP3 can be loosely tied into these broader schemes. Bed-by-bed correlation upwards from the stratigraphically lower ‘6 m Sill’ several hundred meters to the east and bedding-scale sedimentological observations support the inference that BP1 is located in or near ‘Bed 27’ of the Lonfearn Member (Lealt Shale Formation; numbering from [2]). BP3 seems to be located slightly higher in the section based the dominant dip of the stratigraphy (~5–8°, dip direction = ~345°) and its geographic location relative to BP1. The precise stratigraphic position of BP3, relative to BP1, is particularly difficult to determine with confidence due to localized metamorphism, poor exposure of the sedimentary layers along the rocky shoreline, and possible faulting across Lonfearn Burn (John Hudson, personal communication).

These correlations are notable because Bed 27 is the inferred source of the first dinosaur fossil ever discovered on Skye (and indeed, in Scotland): the single footprint (GLAHM V1980), mentioned above, preserved as a positive relief cast (convex hyporelief) on a loose block that had fallen from a cliff further south at Brothers’ Point (~500–600 meters away) [22]. Thus, it is likely that both BP1 and BP3 are closely located stratigraphically to the rocks from which this original footprint derived. It may be possible that one or more of these beds preserve extensive tracksite surfaces across hundreds of meters of lateral continuity, but which are not discernable at outcrop scale because of the patchy nature of the exposures and the extensive metamorphism around Brothers’ Point. Indeed, other track-bearing surfaces appear slightly lower in the section at Brothers’ Point Site 2 which is located immediately above the ‘6 m sill’ in Beds 24 and 25 [25].

## Materials and methods

### Sampling permissions and ethics statement

Permission to conduct the study and collect drone-based data was granted by Scottish National Heritage (SNH). No plant or animal material was collected in this study.

### Track descriptions and measurements overview

The vast majority of the tracks at BP1/BP3 retain infillings or drapes of the overlying sedimentary unit. Although seaweed and some of the encrusting limpets were removed from the track surfaces, the tracks were not prepared further (i.e. the infilling-sediment was not removed) in the context of this study. The qualitative terminology used to describe these tracks follows the definitions found in [37, 38]. Additionally, each track was assigned a numerical value to quantify the quality of preservation according to the scheme of [39] (recently expanded by [40]). These numerical grades (S1 Appendix of S1 Table) allow appropriate caution to be exercised in making ichnotaxonomic assignments and help ensure track comparisons are made between specimens with similar levels of preservation.

Both tracksites are located on active modern tidal platforms. Therefore, the tracks can be measured only during the low tide window. Due to this time constraint, we took summary measurements (track length and track width) in the field while all other measurements were either taken from field photographs using ImageJ 1.51k [41] or from the scaled photogrammetric models of the sites using CloudCompare v2.8.

#### Tridactyl and quadrupedal tracks

**Fig 2:**
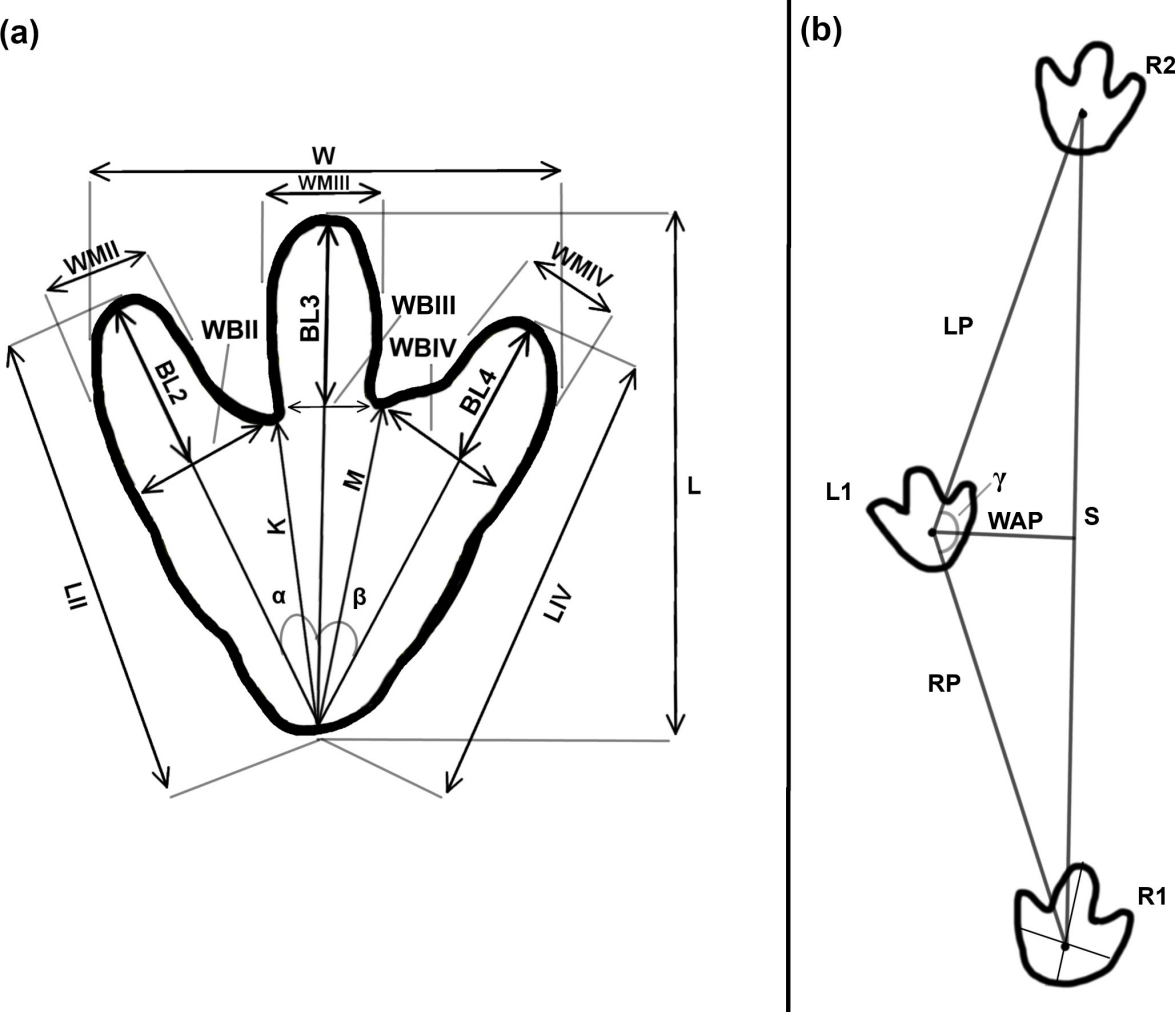
Track measurements for tridactyl tracks and trackways. (a) The measurements taken for each tridactyl track were derived from [37, 38, 42]. The abbreviations on the figure are as follows: total track length (L), total track width (W), total digital length (LII and LIV), basal digital length (BL), basal digital width (WB), middle digital width (WM), the heel-interdigital (hypex) distances (K and M), and the interdigital angles between digits II-III and digits III-IV (α, β). (b) This simplified bipedal tridactyl trackway shows how left and right pace (LP, RP), stride (S), width of angulation pattern (WAP), and the pace angulation (γ) are measured. Additionally, the lines used to define the ‘center of the track’ for trackway measurements are illustrated on track R1.

We collected the following measurements for tridactyl tracks: total track length (L), total track width (W), total digital length (LII and LIV), basal digital length (BL), basal digital width (WB), middle digital width (WM), the heel-interdigital (hypex) distances (K and M), and the interdigital angles between digits II-III and digits III-IV (α,β) (Fig 2). We also noted several additional qualitative features including the presence or absence of claws on the digits, the presence or absence of pads on the digits, the shape of the digits, and the shape of the ‘heel’ [42, 43].

**Fig 3:**
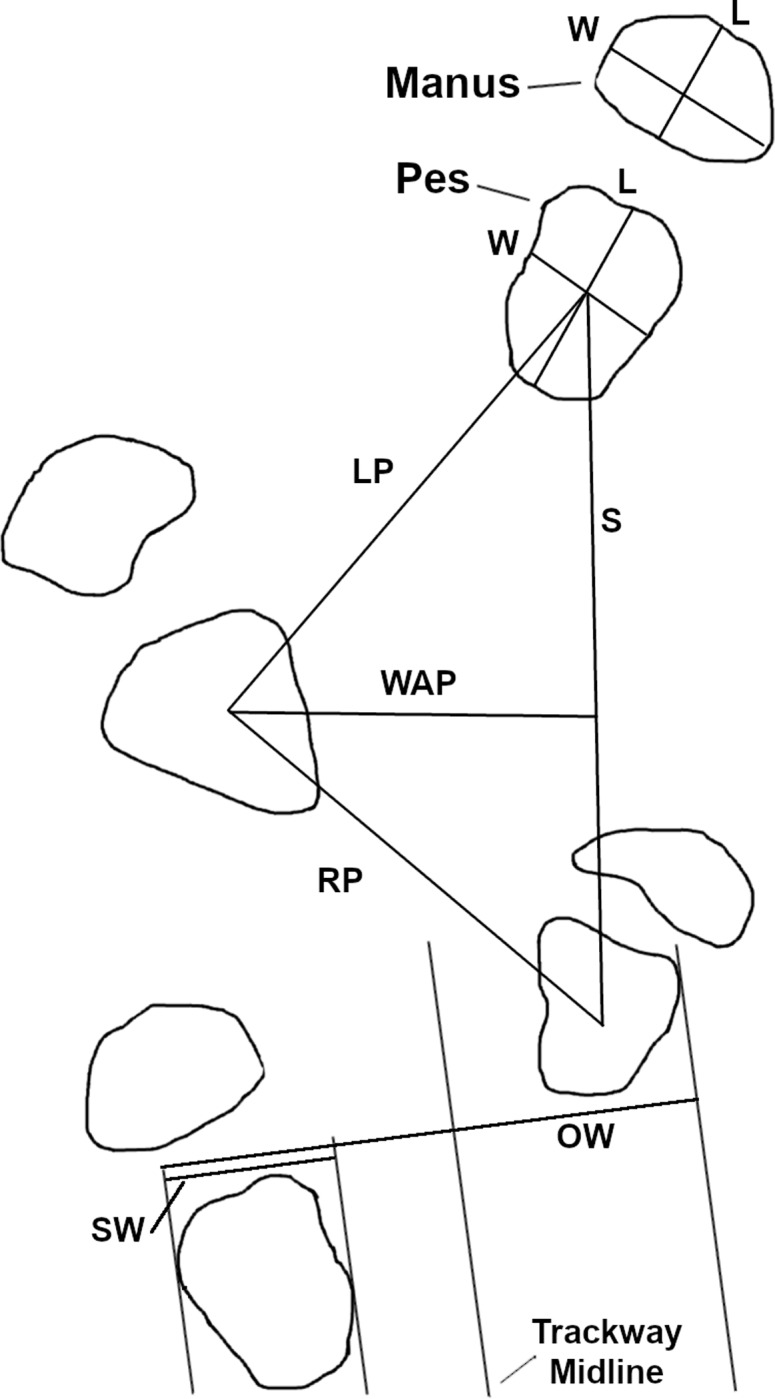
Quadrupedal track and trackway measurements. Length (L) and width (W) show the individual track measurements taken on quadrupedal tracks at Brothers’ Point. Trackway characteristics such as the left and right pace (LP, RP), stride (S), width of angulation pattern (WAP), side width (SW) and overall width (OW) were also measured. Figure adapted from [44].

We measured the manus and pes of quadrupedal tracks with length and width measurements as detailed by [44, 45] (Fig 3). Additionally, we made note of qualitative features like sediment displacement rims, heel marks, and the shape and extent of the digits.

We divided tracks into size classes using the general criteria outlined in [44] for sauropods and bipedal dinosaurs (ornithopods and theropods) and expanded upon by [46] for thyreophorans (S1 Appendix of S2 Table).

#### Trackway measurements

We described bipedal trackways using pace (P), stride (S), width of angulation pattern (WAP), angle of rotation (α), and pace angulation (γ). Fig 2B shows an idealized approach to taking bipedal trackway measurements [44, 47, 48]. The pace of a bipedal trackway is the distance between consecutive, alternating footprints (i.e. right-left or left-right) [49]. Stride length is defined as the distance between corresponding points in successive prints from the same foot (i.e. the distance between one right pes impression and the next) [37, 38]. [38] suggested that the best reference point for bipedal trackway measurements is the “tip of the principal digit” as it is often a well-defined feature. Later workers have broadly adopted this practice using the tip of digit III as the point of reference [44, 50]. This approach, however, proved impractical for the bipedal trackways from Brothers’ Point as they are usually short (3–5 tracks) and poorly preserved without consistently distinctive digit impressions. Therefore, we selected points of correspondence for measuring each individual trackway by assessing the common features between the component tracks of the trackway. In practice, the best correspondence point was defined as the “center of the track” (either the intersection of the axis of digit III and a perpendicular line halfway between the base of digit three and the rearmost portion of the heel or, when the toes or heel were indistinct, the intersection of the long and short track axes).

The width of angulation is a measure of the overall trackway width using established reference points on alternating (right-left) tracks. It can be calculated by applying the Pythagorean Theorem to the pace and stride lengths (*sensu* [44]; Fig 2B). The angle of rotation (α) is the angle between the long axis of a track and the midline of the trackway. Pace angulation of a bipedal trackway is measured as the angle between the lines used for two successive pace measurements (e.g. the angle between the right pace and the left pace as shown in Fig 2B) [38, 44, 49].

We applied the measurements used to describe quadrupedal trackways (pace, stride, width of angulation, and progression) to both manus and pes impressions [38, 44]. The pace, stride, and width of angulation of quadrupedal trackways are defined in the same manner as those of bipedal trackways (Fig 3). Progression describes how far forward the trackmaker moved during a single footfall and was calculated using Eq 1, where WAP is the width of the pes angulation pattern [44]. The progression of the trackways was calculated only for the pes impressions, as manus impressions were sparse in the quadrupedal trackway from Brothers’ Point.

$$Progression={\left[{\left(pace\right)}^{2}-{\left(WAP\right)}^{2}\right]}^{\left(0.5\right)}$$ (1)

The reference point for measurements made between successive quadrupedal tracks is the intersection of the lines along which length and width were measured [37, 38, 44].

Other bipedal and quadrupedal trackway measurements that we took include overall trackway length (measured from the heel of the first impression to the toe of the last) and trackway orientation (measured relative to magnetic north).

In addition to the trackway measures detailed above, we quantified the quadrupedal trackways according to gauge. While sauropod trackways have been divided into 'wide-gauge' and 'narrow-gauge' categories for about a quarter of a century [51], a more quantitative assessment of gauge called the ‘track ratio’ was recently proposed [52]. The track ratio can be calculated using Eq 2, where SW is the side width of the footprint (the width measured perpendicular to the trackway midline) and OW is the overall width (the width measured from the outermost edges of subsequent right and left impressions perpendicular to the trackway midline) (cf. Fig 3, [52]).

$$Track\;Ratio=\left(\frac{SW}{OW}\right)\times 100\%$$ (2)

We used track and trackway measurements to determine the estimated hip height, speed, and gait of the dinosaurian trackmakers. Several different approaches to measuring hip height have been reported in the literature. The most commonly used relationship (Eq 3) correlates footprint length (FL) with hip height (*h*) through a scaling factor [53].

h=4.0×FL (3)

Computer modeling has demonstrated that this equation provides a good estimation of hip heights of bipedal trackmakers [54] and, thus, makes it a reasonable approximation to use for later calculations.

The most effective way to estimate the size of quadrupedal dinosaurian trackmakers, particularly sauropods, is the topic of on-going debate [55]. Thus, Eq 3 was used for the footprints from the quadrupedal trackway to provide a first-order estimation of the hip height. In addition to this estimation, a thyreophoran hip height formula (Eq 4) developed by examining the skeletal proportions of *Kentrosaurus* was used to estimate the hip height (*h*) of the quadrupedal trackmakers from the width of the pes (W) [56].

h=6W (4)

The speed at which a dinosaur was traveling while leaving a trackway can be approximated with the following empirical equation (Eq 5), where v represents the speed in meters per second, g is the gravitational constant (9.81 m/s^2), S is the stride length of the trackway (m), and *h* is hip height (m) [53]. This equation most appropriately applies to trackways left by the animals moving at walking pace (denoted as where the S/*h* ratio is less than 2.0) [44].

$$\text{v}\approx 0.25{\text{g}}^{0.5}{\text{S}}^{1.67}{\text{h}}^{-1.17}$$ (5)

Estimating the speed at which a dinosaur was traveling relies on several assumptions with large amounts of uncertainty. One, in particular, is that the empirical relationships derived from mammal measurements can be extended to dinosaurs and the assumption that foot length is a reasonable proxy for hip height. With that said, the estimates provide a general predictor of speed that is consistent with other biomechanical considerations [57]. Essentially, the uncertainty inherent in determining the speed of dinosaurs must be acknowledged and the results of such calculations regarded as broad estimations rather than strict answers.

The gait of a dinosaur can be qualitatively described as walking, trotting, and running [58]. The points at which an animal transitions between gaits can roughly be defined using the relationship between stride length (S) and hip height (*h*). The relationship, walking < 2.0 < trotting < 2.9 < running, denotes what values of S/h approximate each gait [58].

Broad observations about the rate at which the trackway makers were moving through their respective environments can be made using these relationships. These observations can then support behavioral inferences.

*Ambiguity in tridactyl trackmakers and puzzles of preservation*. A common goal of track analysis is identifying the most likely trackmaking organisms. However, the overall shape of a track is influenced by some combination of three factors–autopodial anatomy (the shape of the foot), its dynamic motion (how the animal is locomoting), and the conditions of the substrate across which it traverses [59]. Frustratingly, the combination of these factors implies both that a single trackmaking organism can produce a variety of different track morphologies (as seen in the case of individual trackways in [60]) or different organisms can produce similar track morphologies. Additionally, the shape of a track can be further influenced by post-registrational taphonomic processes (those that occur after the autopodium has ceased contact with the substrate) like the desiccation of the trackbearing surface preburial or modern erosion [40].

In the case of footprints attributed to dinosaurs, tridactyl tracks prove particularly challenging to reliably assign to definitive trackmakers. Tridactyl tracks are commonly attributed to two clades of dinosaurian trackmakers–theropods and ornithopods–and discriminating between these trackmakers has historically been and continues to be a challenge [42, 61, 62]. The challenge of differentiating between these trackmakers arises because both dinosaur clades possess a functionally tridactyl, mesaxonic pes [63].

Some qualitative parameters including the length/width ratio, shape and length of the toes, presence or absence of claw impressions, and shape of the ‘heel’ have been traditionally used to distinguish between theropod and ornithopod trackmakers [38, 49]. Over the last thirty years, multivariate analyses of linear track parameters and ratios have also seen use in differentiating between theropod and ornithopod footprints [42, 61, 64, 65] and, more recently, geometric morphometric analyses have been brought to bear to investigate the question [62, 66].

However, both the qualitative and quantitative parameters are reliant on assessing a reasonable outline of the footprint in question. This process has been demonstrated to be subjective to the observer as the shape of a footprint and its resultant linear measurements can vary radically based on where and how the track outline is defined [67]. [67] recommends the most objective way to determine the shape of the track is to specify minimum (where the track walls meet the track floor) and maximum outlines (where the track intersects with the tracking surface) to ‘bracket’ the variations within the shape of a single track. In the case of the Brothers’ Point tracks, applying this method is not feasible for many tracks because of the presence of sedimentary infills which obscure both the track walls and the track floor. The 2d track outlines of these tracks generally follow the contact between the infilling sedimentary material and the plane of the trackbearing surface (excluding clear displacement rims or additional soft sediment deformation). These sedimentary infills also make observation of features associated with the track floor impossible and potentially could distort or disturb the original shape of the track impression.

When it comes to the footprints at Brothers’ Point, three main post-registrational taphonomic processes negatively affect the morphological preservation of the tracks–the propagation of cross-cutting dessication cracks from the impressions as the track-bearing surface dries, the subsequent infilling of the impressions by sediment, and erosion from the highly energetic modern tidal environment in which the tracks are exposed [40]. The consistently low preservation grade of the footprints and widespread occurrence of infilling sediments across the two tracksites calls into question the reliability of track shapes reconstructed and, in particular, raises the specter of linear measurements in the study being strongly affected by biases in morphological preservation [40, 67]. In particular, the lengths of the digits and the placement of track hypices are strongly affected by both the depth at which they are measured within the track and by sedimentary infilling [67]. Recognizing these limitations, we refrain from definitively assigning any tridactyl footprints in this study to ichnotaxa. Furthermore, although the likelihood parameters developed in [42] are used to investigate additional lines of evidence for either theropod or ornithopod affinities, the results of such ratios are not relied upon for definitive differentiation between dinosaur clades and instead are used to spur further reflection on possible trackmaker affinities. Thus, although both qualitative and quantitative parameters are used to investigate the likelihood of theropod and ornithopod trackmakers at the sites, the limitations of the footprints at these sites means that clear, unequivocal discrimination between these dinosaur groups is not possible. This uncertainty makes subsequent interpretations of different bipedal dinosaurian trackmakers somewhat speculative.

*Photogrammetric models*: *Data capture and construction*. Recently, unmanned aerial vehicles (UAVs, a.k.a. drones) have been used by paleontologists to document difficult trackway exposures [68–71]. Digital models (e.g. photogrammetric, LiDAR) of tracksites preserve spatial relationships between footprints over large areas and can provide lasting documentation of sites that are particularly susceptible to erosion or that are only exposed for a short time as the result of construction or mining activities [44, 71, 72]. At Brothers’ Point, we sought to automate the process of collecting the photographs for photogrammetric models because of the moderately expansive lateral extent and tidal nature of the tracksites.

At BP1, we used a custom UAV hexacopter built around a Tarot 680 Pro airframe with a payload of a Sony A6000 (24 mp, 20 mm lens) camera oriented at nadir. The Sony A6000 camera was run in aperture priority mode using an external trigger from the flight controller of the UAV. A total of 7 flights were completed over the exposure at varying heights above the ground surface (3 m and 6 m). The UAV was flown along a parallel survey line pattern using Pixhawk flight controllers running Arducopter 3.3 firmware and was operated from a ground control station running Mission Planner v.1.3.41 and communicating with the aircraft over a 433 MHz telemetry link. The flight paths and triggering locations for the aircraft were calculated in Mission Planner prior to the flights and the aircraft were operated in autonomous mode to ensure adequate photo coverage for later photogrammetric work. Twenty-four ground control points (GCPs) were included in the survey. Several markers were placed immediately outside the region of interest while others were spaced ‘randomly’ across the track platform itself. Using preexisting fractures was preferable to hammering directly into the rock, so we preferentially placed the GCP markers within these structures on the outcrop.

The unpredictability of the weather on Skye meant that the drone could not easily be flown at all the tracksites. Thus, to map BP3, we constructed an intervalometer to substitute for the drone. The intervalometer combined principles of time-lapse photography with a paired set of overlapping cameras arranged on a pole to collect a photogrammetric dataset with enough end lap and side lap for model construction. The cameras used to construct the intervalometer were Canon S110 (12 mp). The Canon S110 cameras were modified using CHDK (Canon Hacker Development Kit) firmware to enable external triggering of the camera and greater control of the ISO, aperture, and shutter speed. S1 Appendix contains further details of the intervalometer design and an assessment of its utility. Four GCPs were included in the survey. A similar approach to GCP distribution to BP1 was employed at BP3.

We used relative carrier phase GNSS (Global Navigation Satellite System) data collected using Leica GS10 receivers as GCPs to constrain the photogrammetric models of BP1. On the day of the GNSS survey, a base station was established at the site and allowed to collect location data for 4–5 hours. Individual ground control points on the outcrop were captured using a roving receiver mounted on a bipod-supported pole. The roving receiver remained at each ground control point for approximately five minutes. The ground control points were then paired with the base station to resolve ambiguities and perform an initial quality control check.

We used relative carrier phase GNSS (Global Navigation Satellite System) data collected using Leica SR530 receivers operating in RTK (real-time kinematic) mode as GCPs to constrain the photogrammetric model of BP3. On the day of the GNSS survey, a base station was established at the site and allowed to collect location data for ~3 hours. Individual ground control points on the outcrop were captured using a roving receiver mounted on a pole. Final control point locations were averaged from five field measurements of the location.

In both cases, the cut-off angle for satellites surveyed was 10° above the horizon. Upon returning from the field, the base stations were paired to an established Ordnance Survey reference station. The reference station used (Lochcarron–LCAR) was 44 km from Brothers’ Point.

The GNSS survey of BP1 was refined with precise ephemeris data downloaded from the Internation GNSS Service (IGS) (https://igscb.jpl.nasa.gov/components/prods_cb.html) on April 10, 2017. The estimated position quality for the ground control points was ~1 cm. Precise ephemeris data were not used to refine the results of the GNSS survey of BP3 because the potential location improvements were negligible given the length of the baseline, meaning the quality of the survey would not have been significantly improved. The estimated position quality for the ground control points was ~1 cm for both sites.

We generated a photogrammetric model of each tracksite in Agisoft Photoscan 1.2.5.2735 on the SAMSON workstation of the Airborne Geosciences Facility at the University of Edinburgh. A total of 284 images (from a drone flown at approximately 3 m above the ground surface) were used to construct the tracksite model of BP1. The Ground Sampling Resolution of the generated orthophoto was 0.6 mm/pix. The calculated error for the control points within the model averaged to 7.00 cm with 3.58 cm averaged error for the checkpoints. 460 images (from an intervalometer held approximately 1.5 m above the ground surface) were used to construct the tracksite model of BP3. The Ground Sampling Resolution of the generated orthophoto was 0.3 mm/pix. The calculated error for the control points within the model ranged between 1.74 cm and 6.17 cm for the checkpoints. The model processing protocol both models can be found in S1 Appendix. Links to the photosets used to construct BP1 and BP3 can be found in S1 Appendix.

The accuracy of the models was further evaluated by comparing measurements made in model space with field measurements. The >0.99 correlation coefficient between the field data and the model data yielded for both tracksites indicates that measurements taken from the site models and orthophotos can be considered representative of the reality of the track-bearing surface (S1 Appendix of Fig 6).

### Site overviews

We generated photogrammetric models and orthophotos for both tracksites. A link to high-quality versions of all orthophotos and models is in the S1 Appendix.

**Fig 4:**
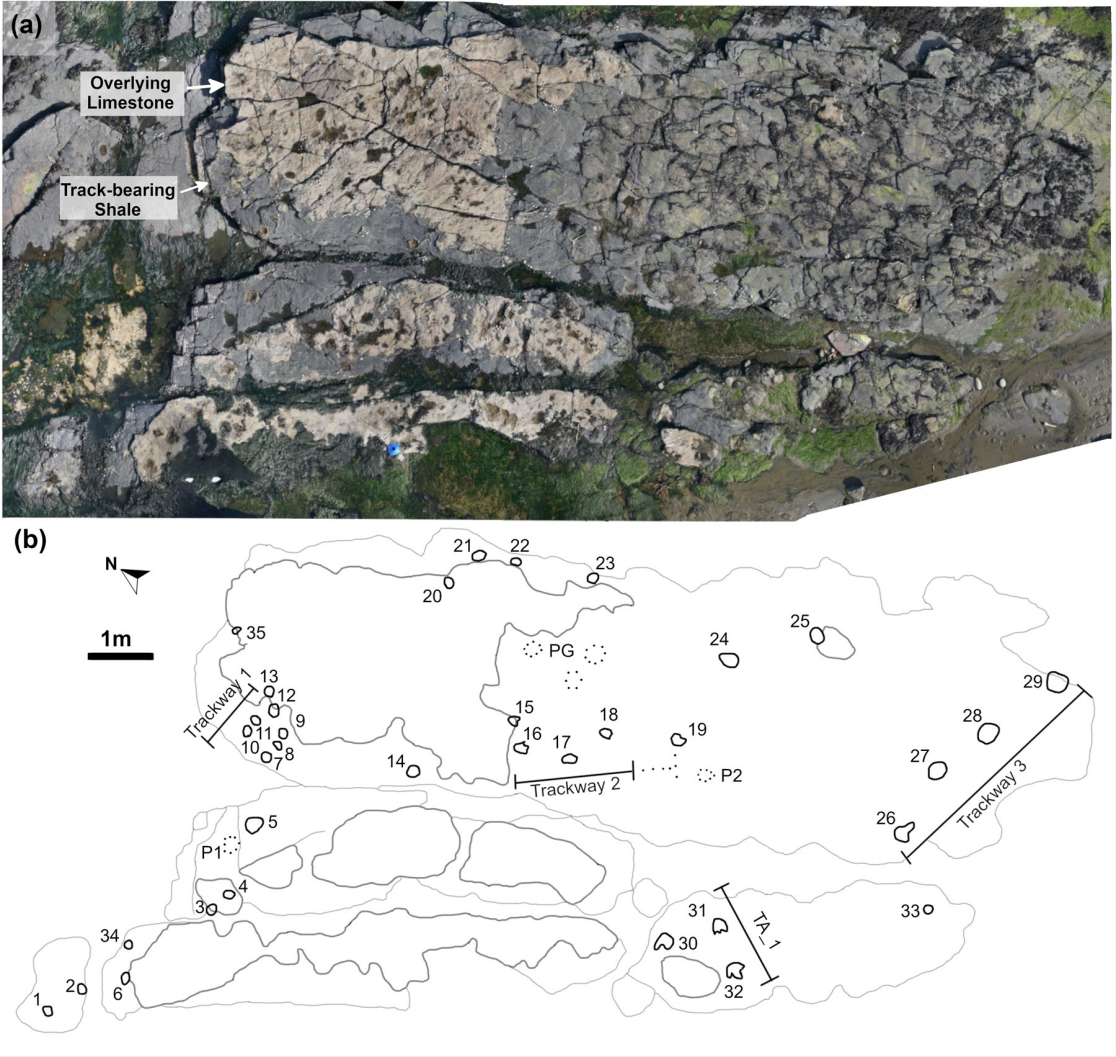
Orthophoto and site map of BP1. (a) The orthophoto for Brother's Point Site 1 (BP1) strikingly demonstrates the high contrast between the dark gray, track-bearing shale and the overlying and in-filling light tan limestone in the Lonfearn Member of the Lealt Shale Formation. (b) The site map highlights the track distribution spatially and denotes the orientation and length of the trackways relative to one another. Each individual track is labeled with its field number (1–35). P1 and P2 denote ‘possible’ tracks 1 and 2 while PG denotes a ‘possible group’ of shallow impressions. Thick black lines denote the track outlines while lighter lines highlight the contrast between the lithologies and fractures on the outcrop. Dark gray lines delineate the extent of the limestone while lighter gray lines show the platform edges of the shale and some fractures within it. One quadrupedal trackway (Trackway 1; BP1_Twy_01), two bipedal trackways (Trackway 2–3; BP1_Twy_02 –BP1_Twy_03), and one set of associated tracks (TA_1) are present at the site.

Thirty-five individual tracks (labeled BP1_01 –BP1_35) were identified at BP1. There are three distinctive trackways (in-text labels BP1_Twy_01 –BP1_Twy_03), a track association (TA_1), and a range of isolated tracks of varying quality. The orthophoto generated from the photogrammetric model (Fig 4A and S1 Appendix) was used as the base for the site map (Fig 4B).

**Fig 5:**
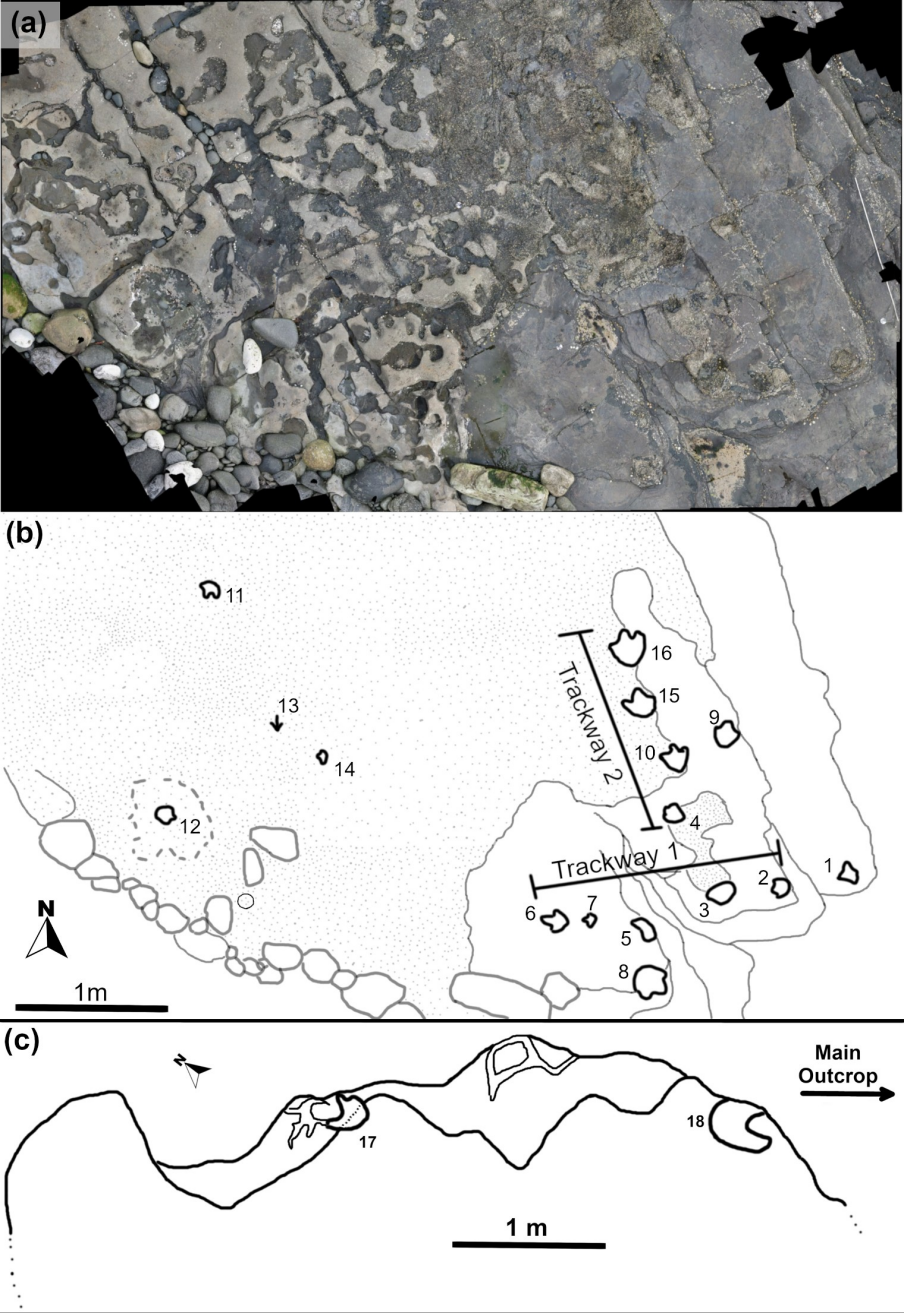
Orthophoto and site map of BP3. (a) The photogrammetric orthophoto of BP3 is oriented relative to geographic north. In this image, the largest concentration of tracks occurs in the southeast corner (lower left). Large tracks can be recognized a tan to gray disturbances in the otherwise smooth surface of the outcrop. (b) Line drawing of the site emphasizing both the locations of individual tracks (labeled with their field numbers; 1–16) and the path which trackways take (with Trackway 1–2 corresponding to BP3_Twy_01–02). The overlying limestone is denoted with light stippling. (c) The small extension of the main bedding plane of the track-bearing layer at BP3 is approximately 5 m to the north. Extensive mudcracks are present on the surface with some of the most prominent ones propagating from the toes of the two tridactyl tracks (17 and 18).

Eighteen tracks were identified at BP3 with sixteen located in the area surveyed for photogrammetric modeling. The tracksite encompasses two bipedal trackways (BP3_Twy_01 and BP3_Twy_02) and a scattering of isolated tracks at varying orientations. The orthophoto generated from the photogrammetric model (Fig 5A and S1 Appendix) was used as the base for the site map (Fig 5B). After the photogrammetric survey was complete, two additional tracks were found in a second exposure of the track-bearing layer approximately 5 m northeast of the main platform. These tracks were mapped separately from the main track grouping at BP3 (Fig 5C).

### Track descriptions and ichnotaxonomy

Comprehensive lists of track measurements and observations for both BP1 and BP3 that are not discussed in-text can be found in S1 Appendix.

### Brothers’ point 1

A variety of track morphologies are preserved at Brothers’ Point 1, including broken casts, several different types of tridactyl footprints, and sub-circular to ovoid tracks that comprise a quadrupedal trackway. Overall, three trackways are observable: the quadrupedal sequence (BP1_Twy_01) and two bipedal ones (BP1_Twy_02 –BP1_Twy_03). The 35 total tracks observed at the site are numbered using a system of BP1_01, BP1_02, etc.

#### Quadrupedal trackway (BP1_Twy_01)

**Fig 6:**
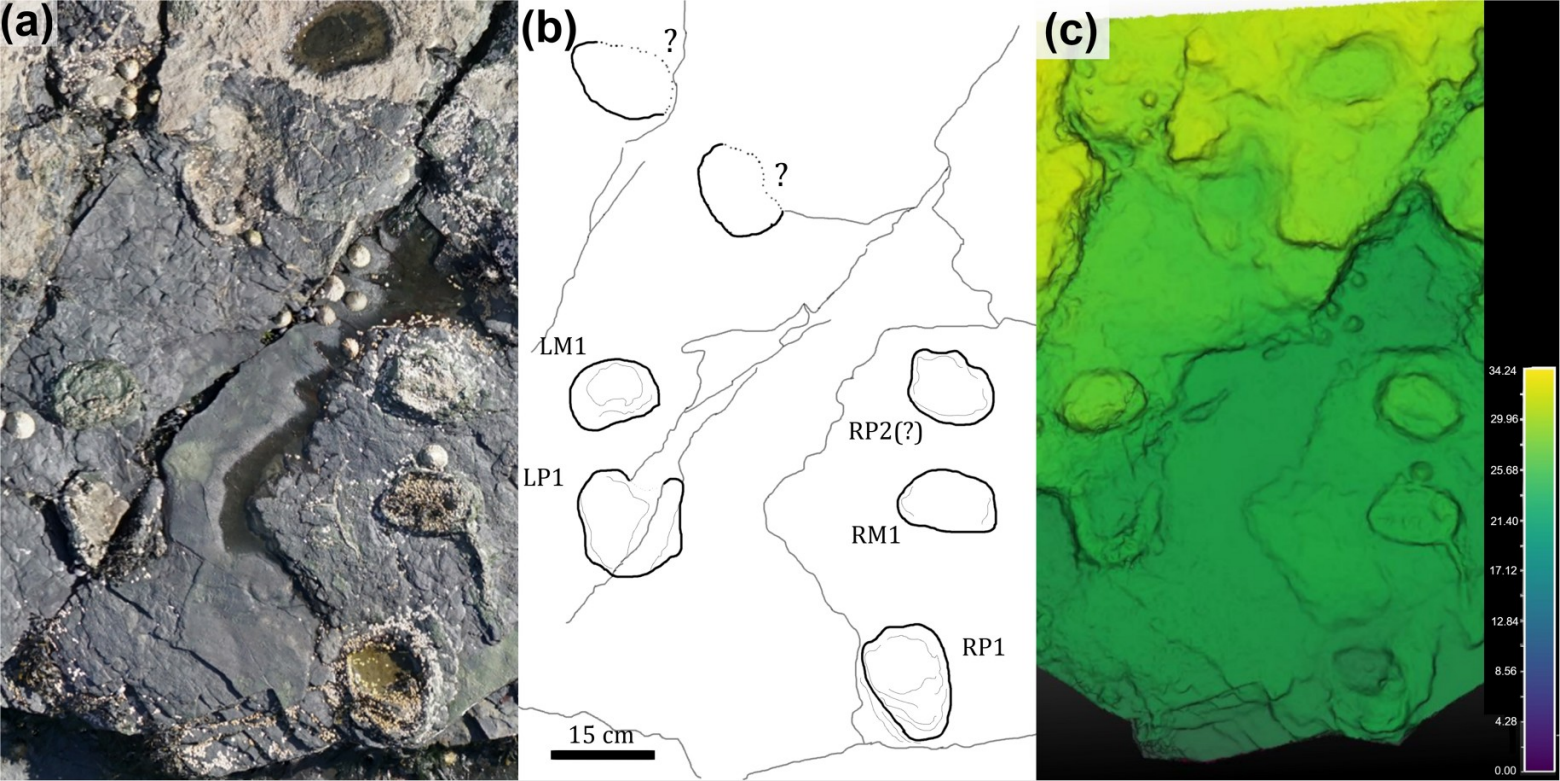
Quadrupedal trackway (BP1_Twy_01) at BP1. The quadrupedal trackway (BP1_Twy_01) shown in an (a) orthophoto from the tracksite photogrammetric model, (b) a line drawing emphasizing distinctive track features, and (c) a false color depth map rendered from the photogrammetric model. Five tracks are clearly associated into the trackway and represent alternating steps with the right manus/pes pair (RM1/RP1) and the left manus/ pes pair (LM1/LP1). Two additional tracks occur immediately above the trackway. However, they are out of line with the trackway progression and their edges are largely obscured by the overlying, light tan limestone layer. Thus, the trackway association is less clear. The color scale of (c) is in units of cm.

Of particular interest among the tracks at the site is a single, short quadrupedal trackway (BP1_Twy_01) that crosses the northern edge of the platform before passing underneath the overlying limestone layer (Fig 4). The trackway (Fig 6) consists of five definitive and two additional possible tracks located out of the expected line of trackway progression. S1 Appendix has a link to a subsample of the outcrop model focused on this trackway.

**Fig 7:**
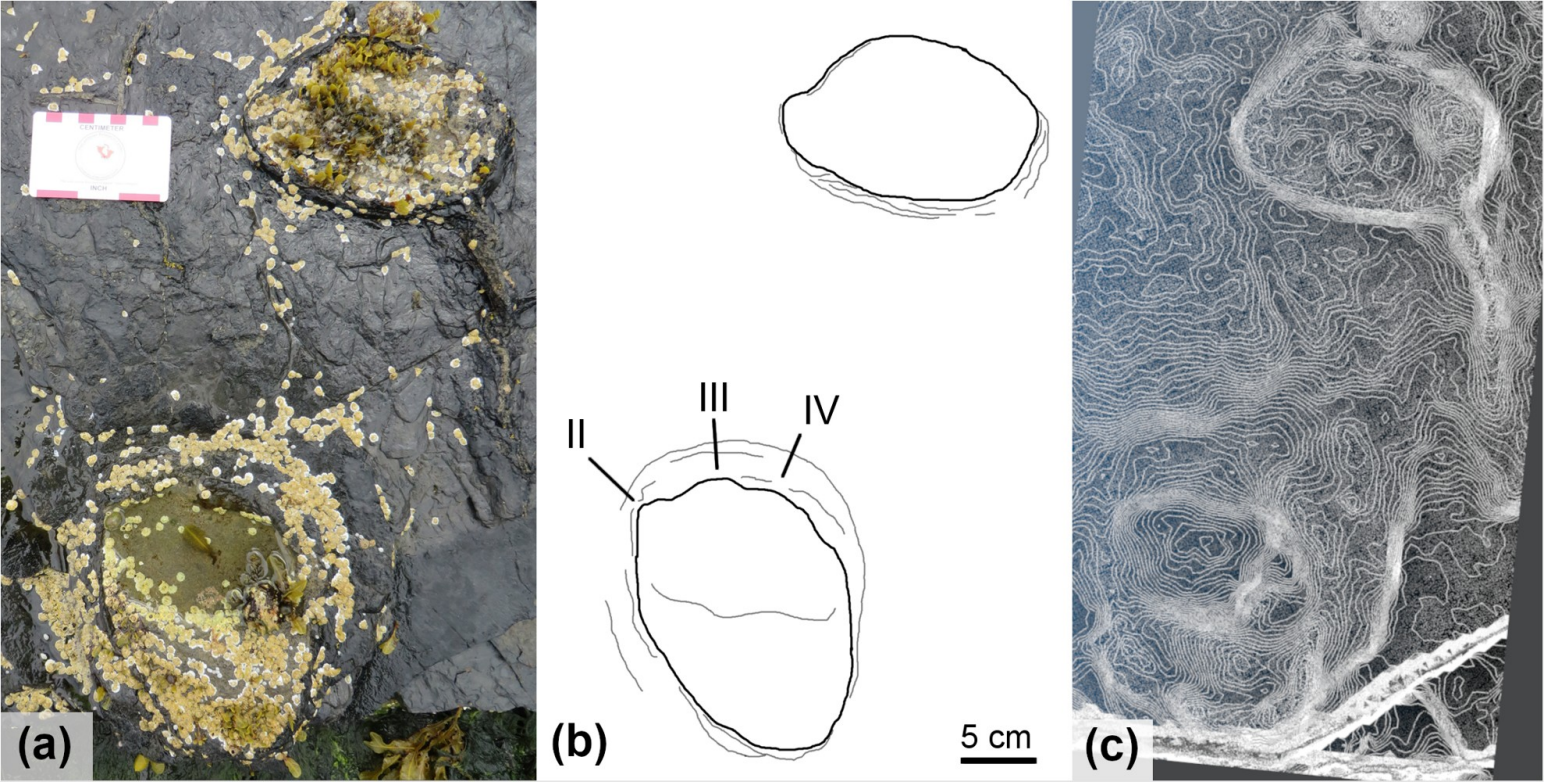
Exemplar manus/pes pair from BP1_Twy_01 at BP1. (a) Field photograph, (b) outline drawing, and (c) contour map of a right manus/pes pair from BP1_Twy_01 highlighting the sediment deformation around each track.

Using the schema outlined in [39, 40], the preservation grade of the tracks in BP1_Twy_01 is 1 because the manus can be distinguished from the pes, there are the remnants of toe marks, and the general outline of the footprints is preserved, but there are not clear indications of ungual marks or digital pads. The manus tracks are crescent-shaped with a convex anterior margin and a less curved posterior margin. The inner edge of the manus tracks has a subtle protrusion on it (Fig 7). Although this protrusion could be the result of the animal’s locomotion, the structure of the manus potentially played a greater role in the formation of this feature. This conclusion is suggested by a relatively low amount of soft-sediment deformation immediately adjacent to the protrusion. This protrusion is possibly the remnant of a pollex impression and makes the manus weakly entaxonic. The average manus length is ~10 cm and width is ~15 cm which yields an average L/W ratio of 1:1.5. The pes tracks are tridactyl with weak indications of short blunt toes along the anterior margin. Digit III is slightly more well-defined than digits II and IV. The pes tracks are weakly mesaxonic, elongate, suboval to subtriangular, and widen anteriorly. The medial and lateral margins of the pes are both nearly straight and the widest point of the pes is located near its anterior margin. The average pes length is ~17 cm and the average width is ~12 cm which yields an average L/W ratio of 1:0.71.

The trackway is 60 cm long (excluding the weakly associated tracks) and oriented at 105°. The manus and pes pace lengths are both ~60 cm. The stride length is ~52 cm between RP1 and RP2. The pes pace angulation is 51.7°. and the width of pace angulation (WAP) of the trackway is ~49 cm.

**Fig 8:**
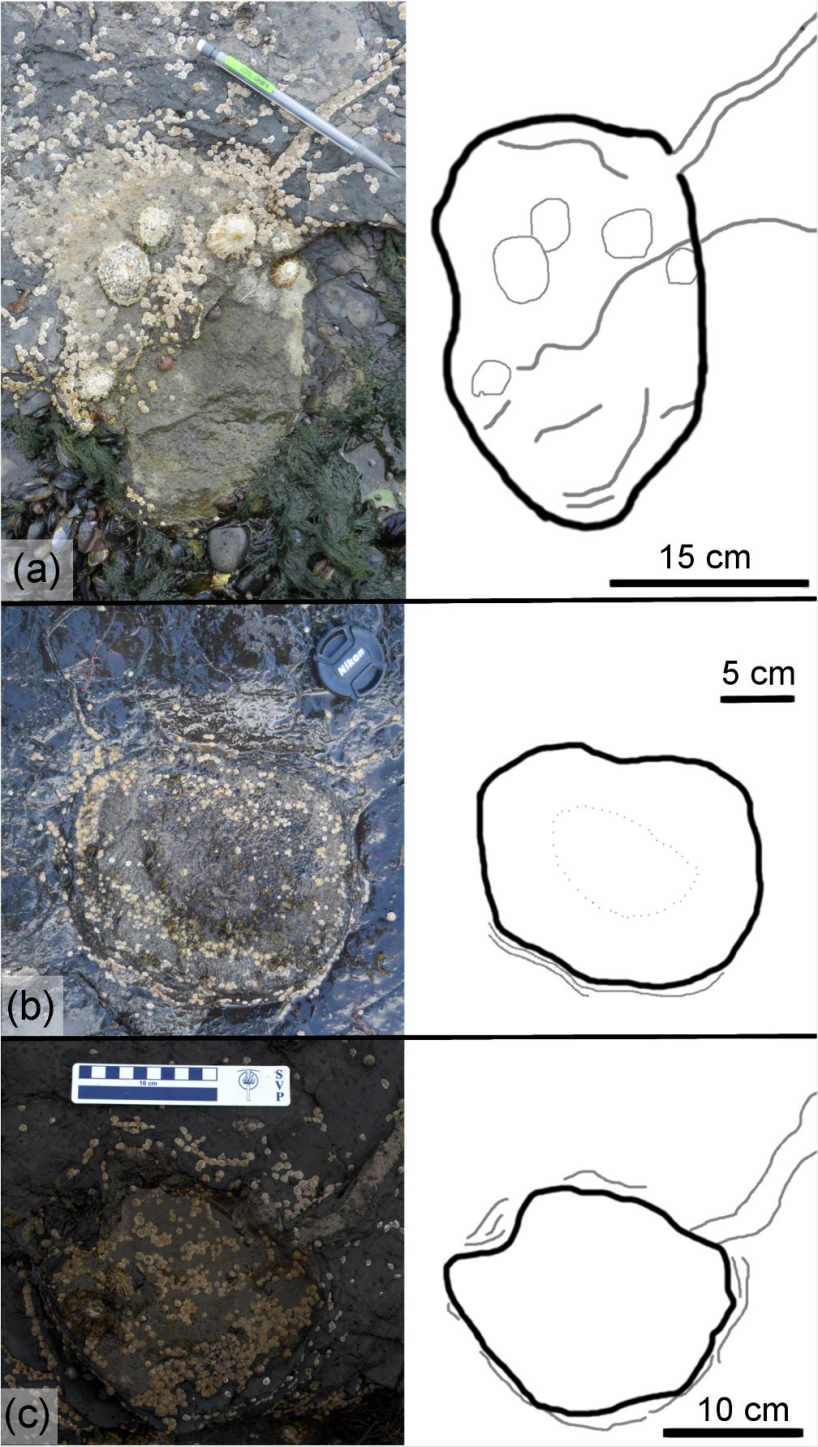
Isolated tracks with similar morphology to BP1_Twy_01 at BP1. Several isolated tracks at BP1 show similar morphologies to those of quadrupedal trackway BP1_Twy_01 from the elongate, subtriangular shape of BP1_03 (a) to the sub-circular presentations of BP1_05 (b) and BP1_14 (c).

Several isolated tracks (BP1_03, BP1_05, and BP1_14; Fig 8) on the platform show similar morphologies to those that compose BP1_Twy_01. BP1_03 is an impression with the remnants of an in-filling cast forming a thin drape over its base. BP1_03 measures 23.8 cm along its long axis and 14.7 cm along its short axis. It has an elongate, subtriangular shape with a rounded, narrow posterior margin widening to the broadest portion of the track near its anterior edge. One of the lateral margins of BP1_03 is slightly concave while the other is more nearly straight to convex. No clear digits are observed on BP1_03. BP1_05 and BP1_14 are both casts filling impressions and are more sub-oval to sub-circular in shape than BP1_03. These more rounded shapes could result, in part, from the influence of the infilling material (which can alter the shape of the underlying track). BP1_05 measures 20.6 cm on its long axis and 16.9 cm on its short axis while BP1_14 measures 21.2 cm and 18.5 cm, respectively. The track margins of BP1_05 are smooth and one of the margins parallel to the long axis of the track is slightly convex while the other is slightly concave. Some soft-sediment deformation-related ridging is present immediately adjacent to the convex margin of the track. The margins parallel to BP1_05’s short axis are nearly straight. Although the long axis margins in BP1_14 exhibit similar curvatures to BP1_05, BP1_14 is notable for preserving hints of a left lateral digit. The lateral margins of the track are straight but angled outwards from a narrower heel toward a broader anterior margin. A large mudcrack propagates outward from the right edge of BP1_14.

The pes impressions of BP1_Twy_01 share many features with the ichnotaxon *Deltapodus* [73, 74]. They are generally subtriangular and broaden from a narrower heel to their maximum width near the anterior margin. Additionally, the pes impressions are mesaxonic with three blunt, but subtle digits along the anterior edge. The lateral margins of the pes impressions and the long axis margins of BP1_03 are nearly straight with minimal curvature as observed in *Deltapodus brodericki* [74]. The manus impressions of BP1_Twy_01 are congruent with those of *Deltapodus brodericki* in possessing a convex anterior margin, being shorter than they are wide, and in being entaxonic. The trackway’s manus impressions differ from those of *Deltapodus brodericki* in being more rounded (rather than having a more pronounced crescentic shape) and in having smooth edges rather than a more irregular outline. Additionally, the pollex impressions in *Deltapodus brodericki* can constitute up to 1/7 overall width of the manus [74], while the protrusion observed on BP1_08 (the most distinct manus) is much less pronounced. A final difference between the tracks at BP1 and the Yorkshire *Deltapodus* is their length/width ratios with the BP1 tracks showing lower ratios (1:1.5, manus; 1:0.75, pes) than the ranges reported for the type series (1:1.75–2.5, manus; 1:1.06–12.5, pes; [73]).

In considering other ichnogenera associated with quadrupedal, non-sauropod trackmakers, it becomes clear that the footprints of BP1_Twy_01 most closely resemble those of *Deltapodus*. The tracks of BP1_Twy_01 are similar to those of *Stegopodus* [75, 76] in possessing a tridactyl pes. However, the pes of *Stegopodus* is transverse (wider than long) with a short heel trace and clearly defined digits with clear hypices between them [75, 76]. In contrast, the pes of *Deltapodus* is longer than it is wide and preserves a more pronounced, elongate heel [73,74]. Additionally, the manus of *Stegopodus* preserves four distinct digits and is ectaxonic (possessing longer outer digits than inner digits) in contrast with the pronounced pollex impression and consequently entaxonic manus of *Deltapodus*. The footprints of BP1_Twy_01 preserve a tridactyl pes that is longer than it is wide and a weakly entaxonic manus and are therefore more consistent with *Deltapodus* than with *Stegopodus*. *Tetrapodosaurus* [77, 78] and *Metatetrapous* [79, 80] are characterized by a tetradactyl pes with distinct toes. The ungual impressions of *Tetrapodosaurus* are more blunted than the conical ones preserved in *Metatetrapous*. These ichnogenera contrast with *Deltapodus’* tridactyl pes that lacks pronounced individual digits. In these respects, the footprints of BP1_Twy_01 are more consistent with *Deltapodus*.

Therefore, despite slight differences in the presentation of the manus and the narrower track aspect ratios, the sum of the observed features supports the assignment of the tracks in BP1_Twy_01 to the ichnogenus *Deltapodus*. Additionally, isolated tracks BP1-03, BP1_05, and BP1_14 also conform to the morphological variations expected of *Deltapodus* tracks (Fig 8). These isolated tracks resemble alternative morphologies in the type series for *Deltapodus* (cf. Fig 3C and 3F, [74]). In particular, BP1_03 exhibits the elongate subtriangular shape, which motivated the naming of the ichnogenus [73], while BP1_05 and BP1_14 both match the more sub-circular morphologies illustrated.

An important caveat in this discussion is that all of the tracks (including the exemplar tracks BP1_07 and BP1_08) are at least partially infilled by sediment. Sediment infills can distort tracks in a variety of ways including (1) changing linear measurements like track length, (2) smoothing track margins, and (3) potentially filling other abiotic features (which would give the track an anomalous shape).

1. While linear measurements can be affected by sediment infilling, the majority of the comparisons that underlie this ichnotaxonomic work rely upon the interactions of multiple measurements (i.e. investigating the aspect ratio of the track). We reason that, unless there is an external factor like a plane of weakness in the surrounding rock, sediment infills would distort the different track axes to a similar extent. Thus, it seems unlikely that the overall aspect ratio of tracks would radically change (i.e. that a track which has an anteroposteriorly directed long axis would not, under the influence of infilling sediment, develop a mediolaterally directed long axis). This reasoning is important when aspect ratio is one of the key factors that differentiates the *Deltapodus* pes (which is anteroposteriorly elongated) from the *Stegopodus* pes (which is mediolaterally elongated). We reason that, despite the sediment infills, the elongate nature of the pes of BP1_07, supports reference to *Deltapodus* over *Stegopodus*.
2. Sediment infills can result in the smoothing of track margins. Functionally, that implies that protruding features (like digits) have the potential to become less pronounced. This effect is indeed a concern as both the three pes digits and the pollex impression of BP1_07 and BP1_08, respectively, are weakly indicated in the track margin. However, on similar reasoning to the above concern about linear measurements, one would expect the smoothing to occur in a way commensurate with the original extent of features. Since the digits of both *Tetrapodosaurus* and *Metatetrapous* are roughly symmetric across the center of the foot, it could be expected that they would be reduced in similar fashions. Thus, in this context, it does not seem likely that the sediment infilling the tracks of BP1_Twy_01 could result in initially tetradactyl tracks appearing tridactyl. Additionally, the presence of a weakly defined pollex on BP1_08 without indication of any other digits in the anterior margin of the track hints that, prior to sediment infilling, this digit was more pronounced. This inferred pronounced pollex is again consistent with the morphology expected for *Deltapodus*.
3. Finally, we can investigate the potential for infilling of abiotic features causing anomalous shapes within these tracks by examining how these infills interact with extensive mudcracks on the trackbearing surface. A mudcrack propagates from the posterior margin of BP1_08 (the manus impression of the exemplar pair of tracks associated with BP1_Twy_01; Fig 7). However, the raised lip of the displacement rim clearly defines the track margins and no clear distortion along the plane of weakness introduced by the mudcrack is observed. Thus, in context of this particular trackway, it appears that the infilling sediment was not emplaced in such a way that existing, clearly abiotic features that intersected with the track introduced anomalies into the perceived shape of the track.

For these reasons, we feel confident that, although the shape of these footprints has undoubtedly been affected by subsequent, post-registration taphonomic process (like in-filling), it remains most consistent with the ichnogenus *Deltapodus*.

Despite the similarity in shape and characteristics between the tracks at BP1 and the type *Deltapodus* tracks from Yorkshire, there are several apparent differences between the two assemblages–particularly in track size and trackway gauge.

**Fig 9:**
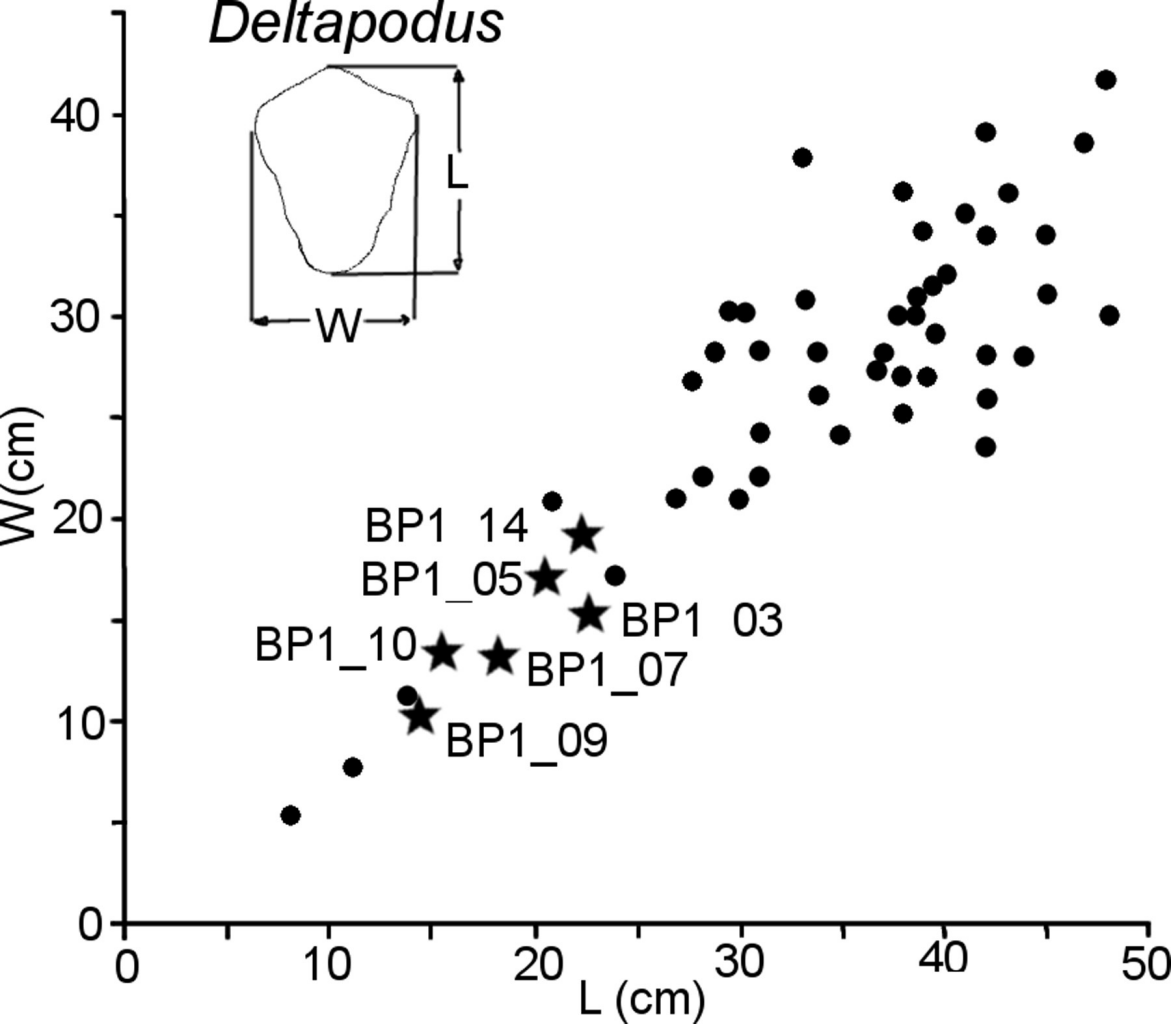
Relative sizes of Skye and Yorkshire *Deltapodus* tracks. The Skye quadrupedal tracks (shown as stars) are plotted with known *Deltapodus* track measurements from Yorkshire (redrafted and modified from Fig 13, [85]). All Skye tracks fall within the general size trends for the ichnogenus.

The majority of the *Deltapodus brodricki* tracks from Yorkshire have lengths of >25 cm (avg. length of ~35 cm with a standard deviation of ~8.8 cm) and *Deltapodus* tracks of a similar size are reported in the western US, Spain, and China [73, 74, 81–84]. The size of the Skye *Deltapodus* tracks (avg. length of 18.25 cm with a standard deviation of 3.59 cm), therefore, seems quite small (approximately half the size of other observed tracks). However, rare tracks from Yorkshire and an additional report of *Deltapodus* from Morocco (length 16.6 cm) extend the size range for the ichnogenus to encompass that of the Skye tracks (Fig 9) [85, 86]. Thus, the Skye tracks fall within the size range observed for *Deltapodus* globally, albeit at the lower end of the range, and may indicate that the trackmaking individuals were indeed smaller bodied than the Yorkshire trackmakers (i.e. either juveniles or members or a smaller-bodied species).

**Fig 10:**
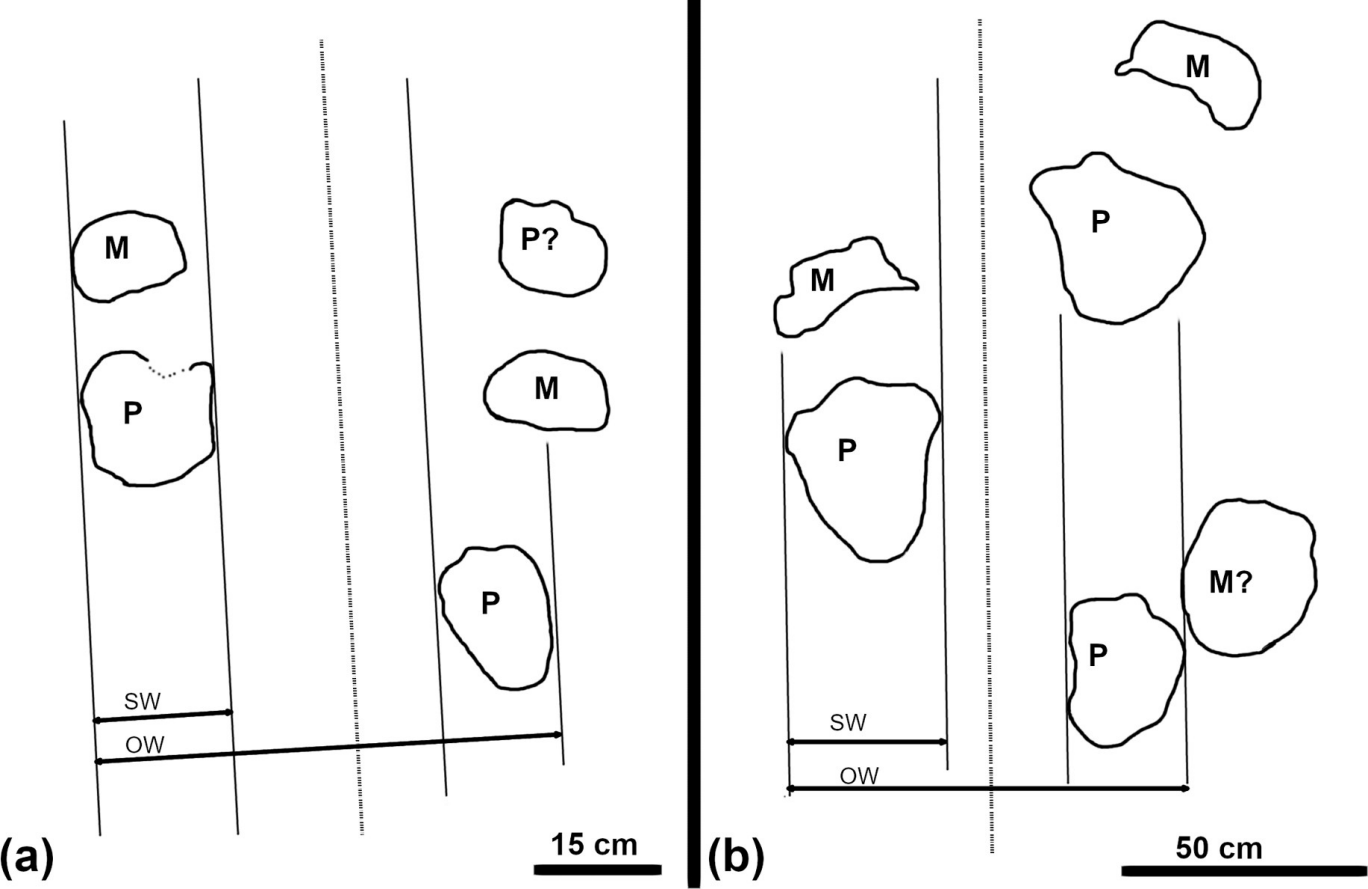
Comparison of *Deltapodus* trackway gauges. (a) The Skye trackway shows a wider gauge than (b) the type *Deltapodus* trackway (redrafted from Fig 7, [63]). M denotes manus impressions, P denotes pes impressions, SW denotes the side width, and OW denotes the outer width. The trackway midline is denoted by a gray dashed line.

The quadrupedal trackway at BP1 exhibits a wide gauge for *Deltapodus*-like trackways, with tracks made by the left manus and pes and tracks made by the right manus and pes separated by ~49 cm. This contrast can be appreciated qualitatively by comparing BP1_Twy_1 (Fig 10A) with the type *Deltapodus* trackway from Yorkshire (Fig 10B). The observation of a wider gauge holds even when normalized for track size using the trackway ratio (*sensu* [52]). The average pes trackway ratio for the type tracks is 29.5% while the average trackway ratio for the Skye trackway is ~23.5% (in the case of this metric, the ratio decreases as the gauge increases). It has been suggested that quadrupedal trackway gauge relates at least in part to both the gait of the organism and the nature of the substrate over which it travels [51, 87, 88] (*contra* [89]) and some of these behavioral or environmental factors may be at play in the case of the wide gauge of this particular trackway.

The hip heights of the BP1 *Deltapodus* trackmaker estimated using Eqs 3 and 4 were quite similar (~ 68 cm and ~72 cm, respectively) and indicate that this trackmaker stood ~ 70 cm at the hip. The progression (distance which the individual travels with a step) of the trackway is low (~5.9 cm) as a result of the extremely wide gauge of the trackway. The speed at which the individual was traveling is estimated to be a walking pace of 40 cm/s (1.45 km/hr). Thus, the trackmaking individual, in this case, was both quite small and moving quite slowly.

#### Bipedal trackways

In addition to the quadrupedal trackway, BP1_Twy_01, a set of associated tracks and two bipedal trackways are present at the site. The track association (TA_1) consists of three clear tracks with diagnostic features. BP1_Twy_02 and BP1_Twy_03 each consist of more ambiguous tracks.

**Fig 11:**
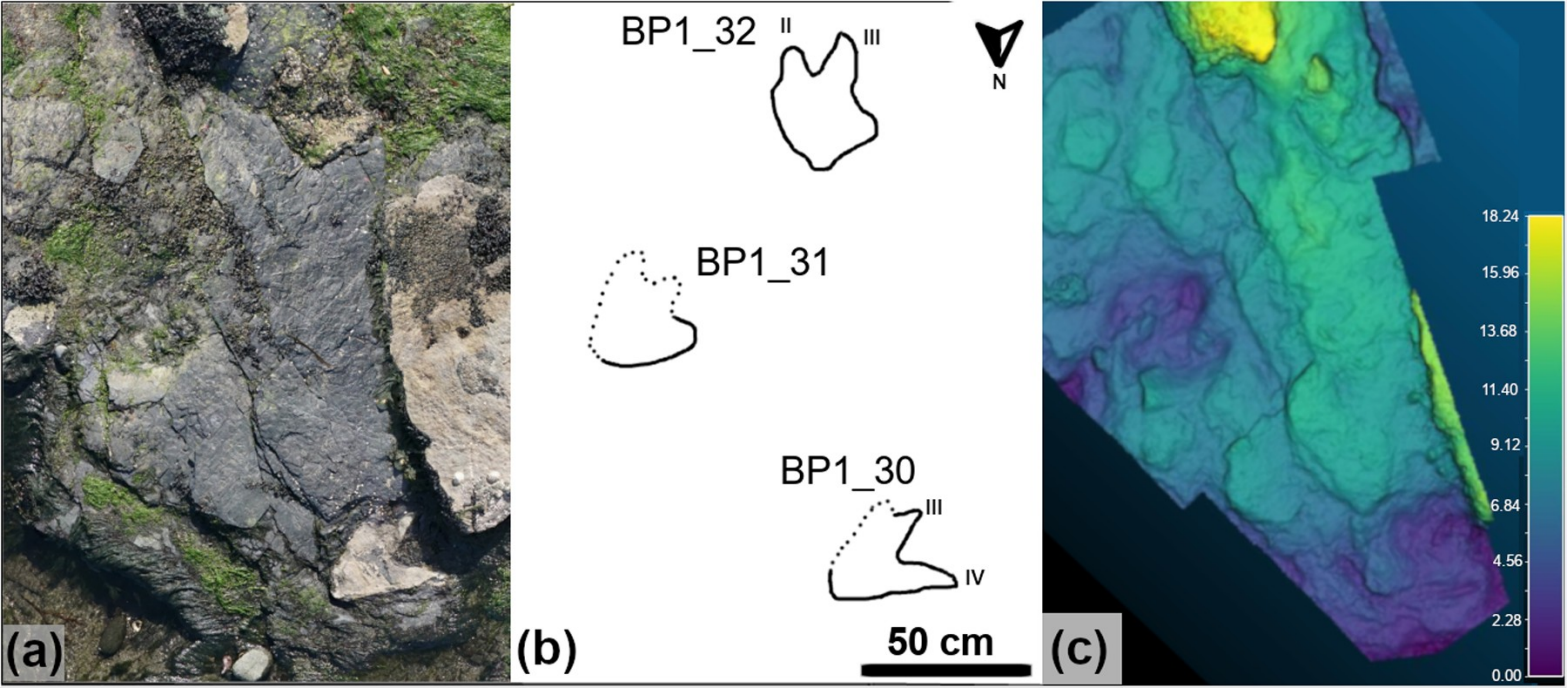
Overview of TA_1. TA_1 shown from (a) the model orthophoto, (b) as a line drawing, and (c) as a false color depth rendering. The three tracks composing the trackway present a variety of preservation styles, with BP1_30 and BP1_32 preserved as limestone casts and BP1_31 as a shallow impression. The impression can be seen in the orthophoto by looking both at subtle changes in vegetation and shading along the left margin and can be more clearly visualized using the false color depth rendering. The color scale of (c) is in units of cm.

TA_1 (Fig 11 and S1 Appendix) is located on the southwestern corner of the tracksite and is separated from the main track-bearing platform by a narrow strip of sand. BP1_30 and BP1_32 both appear to be right pes impressions that are infilled with limestone casts. BP1_31 is an impression that does not have an infilling sediment. It shows less distinctive track boundaries and is primarily recognizable through localized sediment deformation and bowing in the underlying shale layers near its posterior margin. The preservation grade [39, 40] of tracks that compose TA_1 ranges from 0 (BP1_31) to 1 (BP1_30, BP1_32).

**Fig 12:**
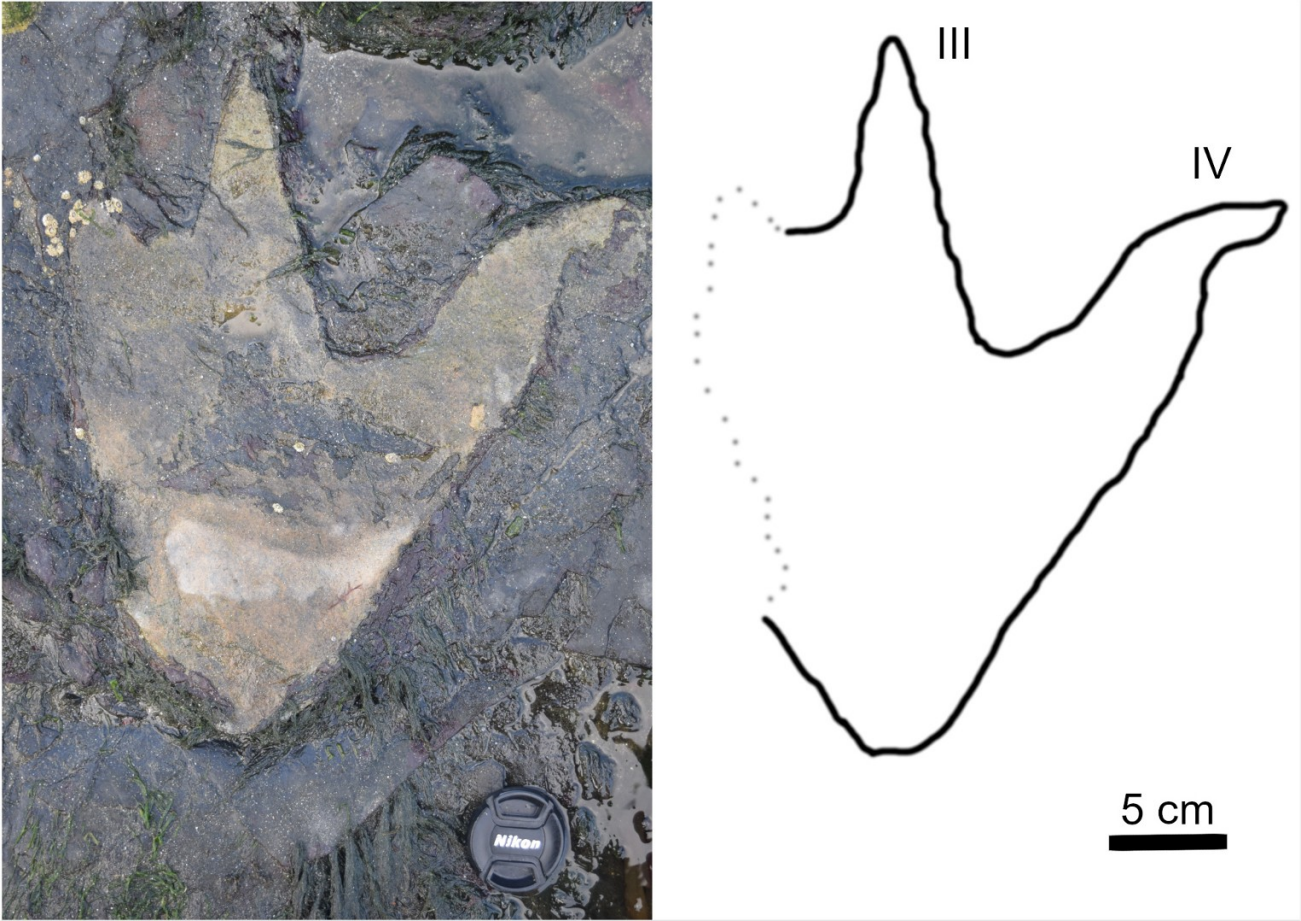
Exemplar track BP1_30. A field photograph and corresponding outline drawing showing the distinctive limestone cast infilling the impression of BP1_30, the most clearly defined track in TA_1. The strong color contrast between the rock types serves to accentuate the shape details of the track including the narrow, pointed digits and the elongated heel.

The tracks composing TA_1 are tridactyl and mesaxonic with narrow, sharply pointed digits (Fig 12). The average length (L) of the tracks is ~39 cm. The heel is narrow, elongated, and tapers to subtriangular posterior margin. The track interdigital angles exhibit a relatively broad range from 20.5° to 35°. Although both BP1_30 and BP1_32 are broken and lack a lateral digit, the remaining lateral digits have a sigmoid curve (likely from the motion of the foot through the substrate). Both BP1_30 and BP1_32 hint at an overall asymmetry in track shape (with BP1_30 showing a straight lateral margin below digit IV and BP1_32 showing a slight notch in the lateral margin below digit II). It is not possible to evaluate whether phalangeal pad impressions are present in these tracks due to the presence of infilling sediment.

Since BP1_30, BP1_31, and BP1_32 are located very near one another on the platform, are all about the same size, and all face in generally the same direction, it is tempting to group them as all being made by the same individual. However, grouping all three tracks into a single trackway requires a non-parsimonious interpretation of the animal’s locomotion (a lateral step within an already wide gauge stance). Therefore, it is unlikely that all three tracks constitute a trackway. However, it is also unlikely that all three footprints are completely unrelated to one another, particularly because they all are facing approximately the same direction on a track-bearing platform that does not show a dominant direction with regard to track orientation. It is more likely that two of the tracks are associated with a single individual and that the third track is from a different instance of an individual walking across the platform. When considering which tracks look most similar, the grouping of BP1_30 and BP1_32 into a partial trackway seems to be favored as both show similar limestone infillings, levels of preservation, and track characters (including a sigmoidal shape in the lateral digits). However, associating these footprints together in the same partial trackway would necessitate a bipedal individual with a relatively wide stance and fairly extreme rotation of the feet away from the track midline. Additionally, both tracks appear to be right pes impressions which raises the question of where the missing left pes impression between them is. In light of this, the group of BP1_31 and BP1_32 seems to be the most likely scenario with the resultant trackway exhibiting a very narrow gauge and strong inward rotation of both feet. Although BP1_31 and BP1_32 are favored as a grouping with BP1_30 being the track formed by an unrelated individual walking in a similar direction, we refer to all three tracks as a track association (TA_1) to reflect continued uncertainty about how these tracks can best be associated with one another.

For BP1_30 and BP1_32 (the two better-preserved tracks in TA_1), their general morphology (elongate track shape and narrow toes) suggests a likely theropod affinity for the trackmaker. However, the measured track parameters (Table 1), show a mixture of theropod and ornithopod affinities. Both assessed tracks displayed a slightly greater ornithopod affinity based on whole track characteristics, while parameters specifically focused on ratios from digit measurements tended to yield strong theropod affinities. Due to the overall shape of the tracks (particularly the pointed nature of the toes), the 'digit-focused' parameters are considered more representative of trackmaker, which we interpret to most likely be a theropod.

**Table 1:** Track parameters and assessment of the trackmaker affinity of TA_1 at BP1.

Track Parameters	Threshold values and probability that the track is either theropod or ornithopod	BP1_30	BP1_32
L/W	80.0% Theropod > 1.25 > Ornithopod 88.2%	n/a	n/a
L/K	70.5% Theropod > 2.00 > Ornithopod 88.0%	1.37	1.79
L/M	65.0% Theropod > 2.00 > Ornithopod 90.7%	1.77	1.93
BL2/WMII	76.1% Theropod > 2.00 > Ornithopod 97.4%	n/a	2.35
BL3/WMIII	72.7% Theropod > 2.20 > Ornithopod 97.7%	4.34	2.27
BL4/WMIV	76.1% Theropod > 2.00 > Ornithopod 97.6%	2.95	n/a
LII/WBII	84.6% Theropod > 3.75 > Ornithopod 90.2%	n/a	4.12
LIII/WBIII	70.6% Theropod > 4.00 > Ornithopod 91.5%	4.37	4.40
LIV/WBIV	73.7% Theropod > 3.75 > Ornithopod 93.4%	4.55	n/a

Track measurements (as outlined in Fig 2) from TA_1 were used to determine the likelihood of the track having either theropod or ornithopod affinity [42]. Both tracks demonstrated a slightly more ornithopod affinity based on whole track characteristics, while more 'digit-focused' parameters consistently yielded a theropod affinity. The blank spaces in the table reflect parameters that are incalculable because the track was broken.

Since these tracks have low preservation grades and outlines that are heavily influenced by the in-filling sediments, they cannot be confidently assigned to any ichnogenus. Although a firm ichnotaxonomic diagnosis cannot be made, we observe that these tracks share some similarities with the ichnogenus *Megalosauripus* [90] and could be further discussed with reference to this ichnogenus. The tracks conform to the diagnostic characteristics of this ichnogenus in being large, tridactyl, and having an elongate heel relative to digit III [90]. [50] observes that some *Megalosauripus* tracks have pointed claw marks and have low (22–40°) interdigital angles. TA_1 manifests all of these characteristics, with the tracks only differing from the extended description of [50] in lacking a “squared U-shaped metatarso-phalangeal impression”.

Interestingly, *Megalosauripus* tracks exhibit some of the supposed ‘ornithopod’ affinities seen in the Skye tracks, particularly a low length to width ratio. *Megalosauripus* tracks, while still being elongate, tend to have a lower length to width (L/W) ratio than other large theropod ichnogenera. For example, the L/W ratios of BP1_30 and BP1_32 (1.20 and 1.14) fall near to the range of ratios (1.24–1.39) described for ~80 *Megalosauripus* tracks from Portugal [50]. This ratio is far below the observed L/W ratios for the large theropod ichnogenus *Eubrontes* (~1.5, measured and averaged from Fig 5A and 5B [91]). The tracks of TA_1 do not correspond with *Eubrontes* because they lack distinctive phalangeal pad impressions and digit III has a shorter relative length and parallel (instead of spindle-shaped) sides [90, 91]. *Kayentapus* [92, 93] is another large theropod ichnogenus with a low L/W ratio caused by relatively broad interdigital angles ((II^III) = ~34 and (III^IV) = ~29). These values were averaged from UCMP 83668–1 and UCMP 83668–4, the two most distinct tracks of the type trackway [93]. However, TA_1 has a lower L/W ratio than *Kayentapus* and smaller interdigital angles. Additionally, the constituent tracks of TA_1 do not show the pronounced extension of digit III evident in the type series of *Kayentapus*. Furthermore, although it should be noted that this features are strongly controlled by the nature of the substrate, TA_1 does not correspond to *Kayentapus* because the extent of their ‘heel’ is more pronounced than the strongly digitigrade presentation observed in the type series [92, 93]. The presence of the distinctive phalangeal pad impressions associated with *Kayentapus* could not be assessed for these tracks because the infilling sediment obscures the base of the track.

**Fig 13:**
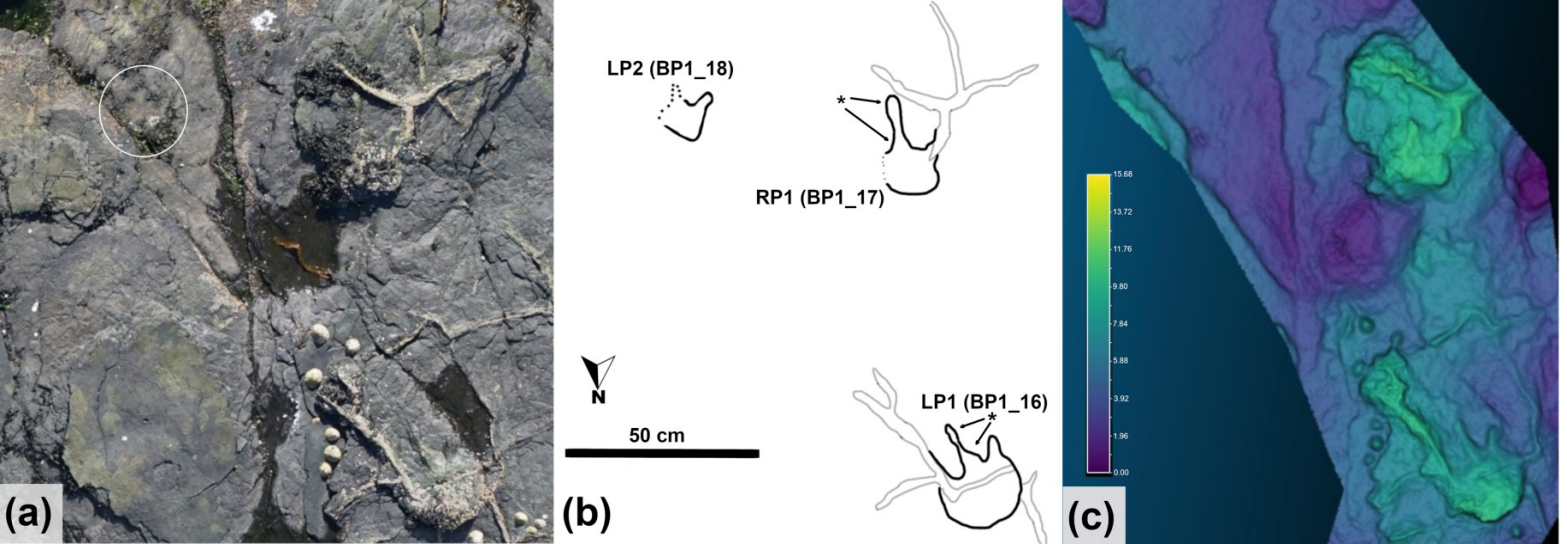
Overview of BP1_Twy_02. (a) The orthophoto of BP1_Twy_02 is shown with (b) a line drawing highlighting distinctive features of both the tracks and the desiccation cracks that preferentially propagate from the toes of the tracks and (c) a false color depth map of the trackway. The line drawing particularly emphasizes the presence of phalangeal pads on digit III of LP1 and RP1 with arrows (labeled *) pointing out their location within the tracks. The white circle on the (a) shows the location of BP1_18 since it is difficult to discern in both the orthophoto and the depth map. The color scale of (c) is in units of cm.

**Fig 14:**
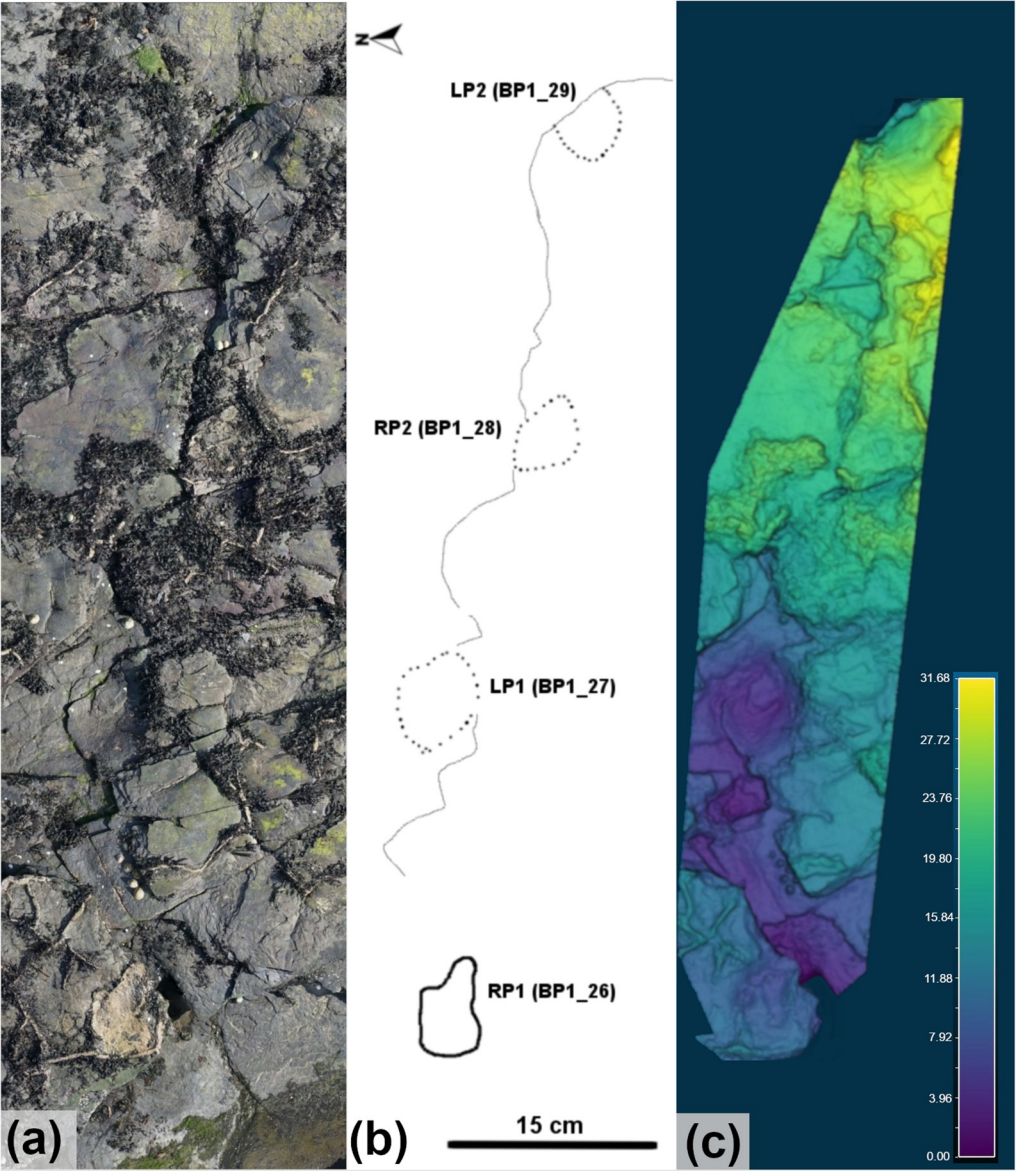
Overview of BP1_Twy_03. BP1_Twy_03 is shown as an (a) orthophoto, (b) a line drawing, and (c) a false color depth map. The trackway spans the whole southern portion of the main track-bearing platform with BP1_29 being present solely as a broken heel mark on the platform edge. The color scale of (c) is in units of cm.

The two bipedal trackways, BP1_Twy_02 and BP1_Twy_03, are not as well-preserved as TA_1. BP1_Twy_02 (Fig 13; link in S1 Appendix) is located towards the center of the main track-bearing platform. Its line of progress is generally southward and the trackway seems to emerge from underneath the overlying limestone bed. The trackway consists of three tracks (BP1_16, BP1_17, and BP1_18). The first two are positive relief casts while the third track is a shallow impression. The casts are crosscut by large desiccation cracks. The preservation grade [39, 40] of the positive relief casts is 1 while the shallow impression does not preserve clear morphological details and is therefore at grade 0. BP1_Twy_03 is located on the southern side of the track-bearing platform. BP1_Twy_03 (Fig 14, link in S1 Appendix) is the most poorly preserved of the trackways at the site. The preservation grade [39, 40] of the tracks in BP1_Twy_03 is 1 for exemplar track BP1_26 and 0 for the subsequent deep impressions.

BP1_Twy_02 is 1.73 m long with an orientation of 155°. The average pace length is ~79 cm and the stride length is 1.45 m. The pace angulation is 131° and the width of pace angulation (WAP) of the trackway is 50 cm. The individual track rotation for BP1_Twy_02 is towards the midline (negative angle). BP1_Twy_03 is oriented at ~100° and is 3.8 m long. The pace and stride lengths are essentially regular throughout the trackway. The average pace length is 1.19 m and the average stride length is 2.31 m. The average pace angulation of the trackway is 153°.

**Fig 15:**
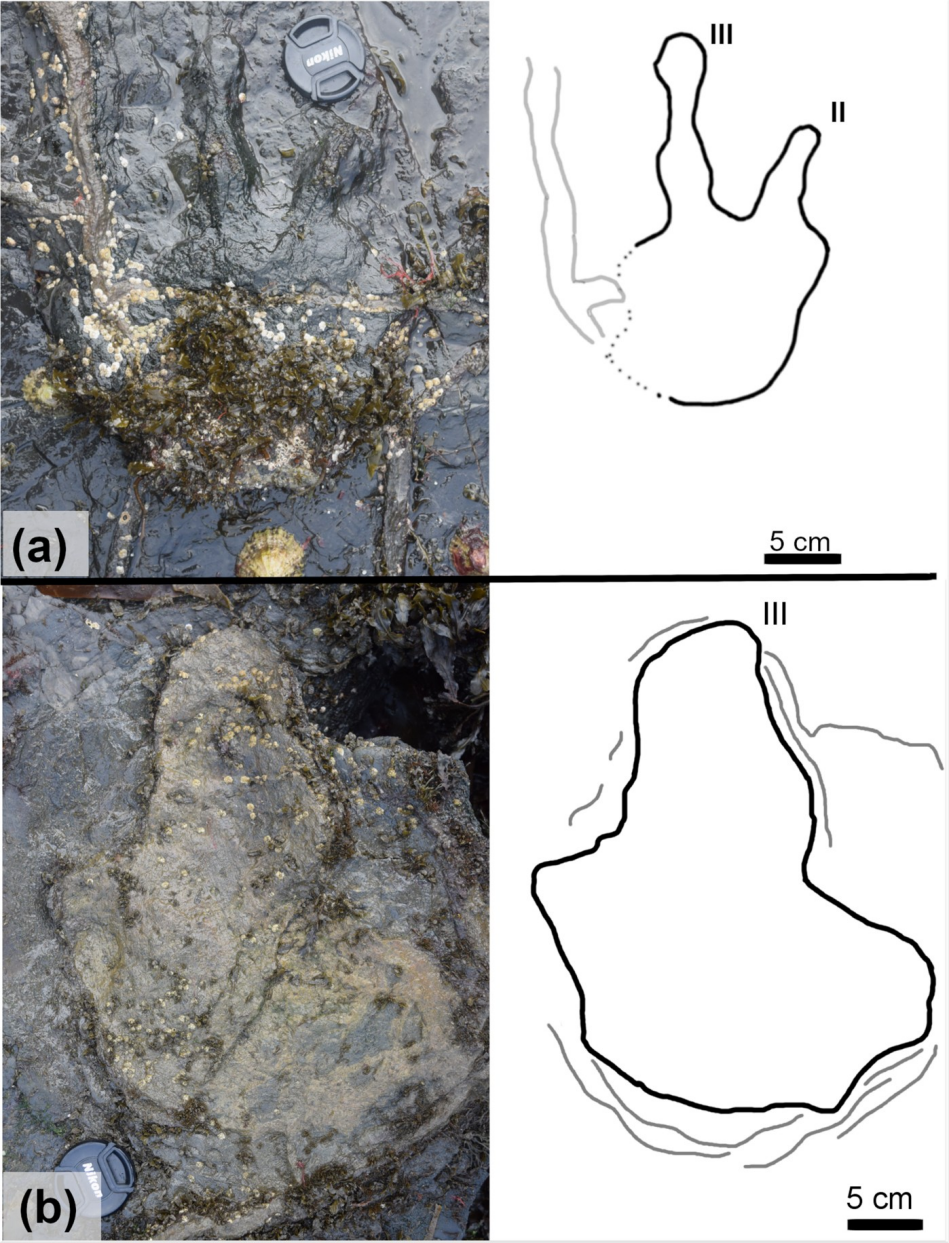
Exemplar tracks from BP1_Twy_02 and BP1_Twy_03. (a) A field photograph and outline drawing of BP1_16 illustrating the presence of remnant phalangeal pads on the positive relief casts present in BP1_Twy_02. (b) A field photograph and outline drawing of the most clearly defined tracks in BP1_Twy_03 (BP1_26). The track is preserved as a broken limestone cast (modifying the true track) set in an area of strong sediment deformation. This deformation is illustrated on the line drawing as thin gray lines concentrated around the track’s heel.

Although many of the features of the positive relief casts in BP1_Twy_02 were later obliterated by the propagation of the desiccation cracks, BP1_16 and BP1_17 show widenings of digit III that could be the remnants of phalangeal pads (Fig 15A). Two such widenings are on each track: one at the base of digit III and the other at the most distal end of the digit. In addition to the long, narrow toes exhibited by each track in the trackway, the presence of these pads strengthens the inference that the trackmaker was a theropod. The presence of distinctive phalangeal pads along the toes is more characteristic of theropod tracks than ornithopod tracks [94]. Although the presence of probable phalangeal pads on the tracks suggests that the component tracks of BP1_Twy_02 are more likely made by a theropod trackmaker, we refrain from assigning them to a ichnogenus.

The only track in BP1_Twy_03 that displays distinctive features is BP1_26 –the first track in the trackway sequence. Like the majority of the other tracks at the site, BP1_26 (Fig 15B) is a modified true track since it is infilled by a limestone cast. Both edges of the track are broken and only one digit (III) is clearly visible. It is likely that the track was originally tridactyl and mesaxonic. The length of the BP1_26 is ~33 cm, which places the bipedal trackmaker in the large size category. This track length was used for subsequent hip height and speed estimations (Table 2) as the features of the other tracks composing the trackway were indistinct. The other tracks in BP1_Twy_03 (BP1_27, BP1_28, and BP1_29) are deeply impressed in the underlying shale bed, display indistinct margins, and are partially infilled with limestone casts. Although the track impressions are deep (as seen in particularly with BP1_27 in Fig 14C), the angles of the bedding deflections around them are shallow. The highly localized sediment deformation around the tracks, which is particularly visible along the platform edge with BP1_29 (LP2), indicates that these impressions are indeed footprints.

**Table 2:** BP1 bipedal trackway measurements.

	N	RP[1]	RP[2]	LP	S[1]	S[2]	WAP[1]	WAP[2]	γ[1]	γ[2]	α	Length	Orientation
BP1_Twy_02	3	0.70	n/a	0.88	1.45	n/a	0.50	n/a	131	n/a	-10.4	1.73	155
BP1_Twy_03	4	1.28	1.04	1.24	2.34	2.28	0.39	0.50	150	156	n/a	3.80	100

The measurements of the bipedal trackways present at Brother's Point Site 1 are summarized here. All measurements are in meters except for γ, α, and trackway orientation (measured in degrees). N is the number of tracks in each trackway. RP[1] and RP[2] are the right pace measurements taken between RPx and LPx of the trackways respectively (track designations figured on outline drawings). LP represents the left pace measurements. S[1] and S[2] are the stride lengths between the right feet and the left feet, respectively, in BP1_Twy_03 and S[1] denotes the only measurable stride length in BP1_Twy_02. For BP1_Twy_03, WAP[1] is the trackway width measured between RP1 and RP2 with LP1 as the focal point while WAP[2] is the trackway width measured between LP1 and LP2 with RP2 as the focal point. WAP[1] for BP1_Twy_02 denotes the trackway width measured between stride line and the opposite footprint (RP1). γ is the pace angulation of the trackway. For BP1_Twy_03, γ is the pace angulation with [1] using RP1 as the vertex of the angle and [2] using LP2 as the vertex of the angle. α measures the track rotation of footprints with regard to the midline. LP1 in BP1_Twy_2 demonstrates inward rotation (a negative angle).

All measurements related to the bipedal trackways at BP1 are summarized in Table 2.

Since variations in the shape of dinosaur footprints result from the combination of gross pedal morphology, the motion of the foot through the substrate, the consistency of the substrate, and post-registrational taphonomic processes [40, 59], variations in track morphology observed in the trackways and track association the site do not definitively imply that there are multiple trackmaking theropod species at BP1. Using an average of the track lengths in each trackway, the hip heights of the trackmakers for BP1_Twy_02, BP1_Twy_03, and TA_1 were calculated (Table 3). Relative stride lengths (S/h) of <2 indicate walking gaits for the two trackway trackmakers (Table 3).

**Table 3:** BP1 theropod size and velocity estimations.

	Approximate Trackmaker Hip Height (cm)	Estimated Velocity	
		m/s	km/hr
BP1_Twy_02	116	1.2	4.4
BP1_Twy_03	131	2.3	8.3
TA_1	155	-	-

Based on the range of hip heights estimated, at least two sizes of bipedal trackmaker were present at BP1 with TA_1 being made by the largest individual and the trackmaker of BP1_Twy_02 estimated to be moving slower than that of BP1_Twy_03.

While [53]’s formulation to estimate hip height is a coarse measurement (due to variation of the anatomy/posture of different organisms and the effects of substrate and dynamic motion on the pes length), the wide range in estimated sizes for the theropod tracks at the site (~116 cm to ~155 cm) hints that at least two individuals traversed the mudflat.

#### Additional tridactyl track morphologies

**Fig 16:**
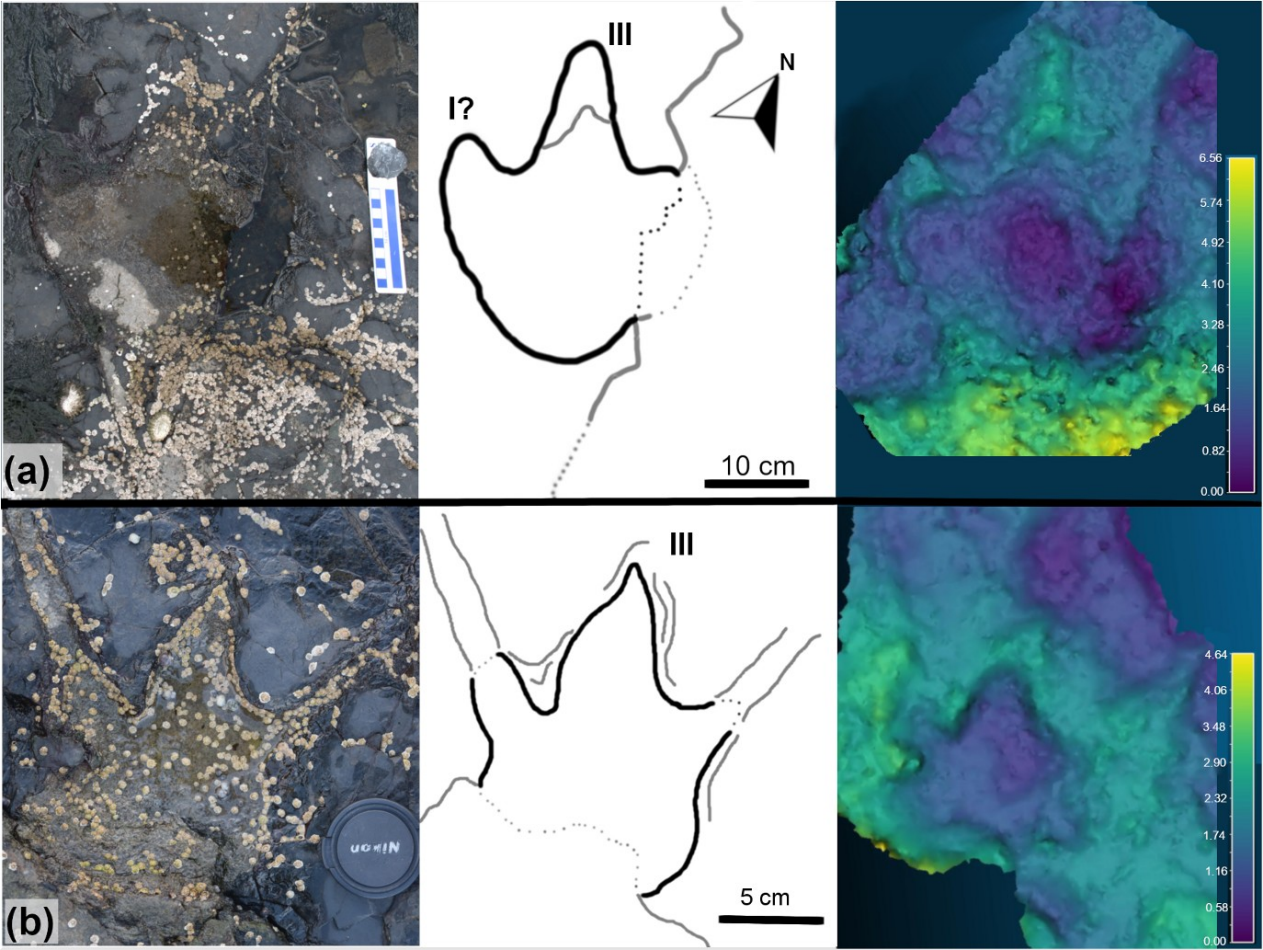
Additional tridactyl track morphologies from BP1. (a) Although broken, BP1_01 (shown with a field photograph, corresponding line drawing, and false color depth map) demonstrates the strongest ornithopod affinity of any of the tracks present at the site. (b) BP1_06 is shown with a field photo, a corresponding line drawing, and a false color depth map. The track preserves strong sediment deformation around the toes and the hypices and desiccation cracks propagate from the lateral digits. These sedimentary features are denoted with light gray outlines on the line drawing. The diameter of the camera lens is 5 cm. The color scale of both false color depth maps is in units of cm.

BP1_01 (Fig 16A) is located in the northwestern-most portion of the track-bearing platform. The track is eroded and is missing one of the lateral digits. Since the track is not associated in a trackway, digit numbering is arbitrary with the complete left lateral digit referred to as digit I and the incomplete right lateral digit as digit IV. BP1_01 is mesaxonic and tridactyl. The digits taper to slightly pointed tips (but do not show distinctive claw impressions) while the heel of the track is moderately broad. The preservation grade [39, 40] of this track is 1.

The track length is 32.5 cm, which places it in the large bipedal size class (S1 Appendix of S2 Table). The interdigital angle between digit II and digit III is 30.7°. The affinity of this track is difficult to assess qualitatively because elongate toes with pointed tips are associated with theropods while a broad heel is more characteristic of ornithopods. Although not all parameters could be calculated, the likelihood analysis of [42] was used to determine the most likely affinity of BP1_01 (Table 4).

**Table 4:** Assessment of the trackmaker affinity of BP1_01.

Track Parameters	Threshold values and probability that the track is either theropod or ornithopod	BP1_01
L/W	80.0% Theropod > 1.25 > Ornithopod 88.2%	1.32
L/K	70.5% Theropod > 2.00 > Ornithopod 88.0%	1.64
L/M	65.0% Theropod > 2.00 > Ornithopod 90.7%	1.67
BL2/WMII	76.1% Theropod > 2.00 > Ornithopod 97.4%	1.70
BL3/WMIII	72.7% Theropod > 2.20 > Ornithopod 97.7%	4.1
BL4/WMIV	76.1% Theropod > 2.00 > Ornithopod 97.6%	n/a
LII/WBII	84.6% Theropod > 3.75 > Ornithopod 90.2%	3.51
LIII/WBIII	70.6% Theropod > 4.00 > Ornithopod 91.5%	4.27
LIV/WBIV	73.7% Theropod > 3.75 > Ornithopod 93.4%	n/a

Track measurements from BP1_01 were used to determine the likelihood of the track having either theropod or ornithopod affinity. Comparing the track parameters with [42] results in a further indeterminate result. In terms of overall track shape parameters, the track consistently presents as ornithopod while ratios of the measurements taken on individual digits yield mixed theropod and ornithopod results. The blank spaces in the table reflect parameters that incalculable because the track was broken.

This probabilistic analysis slightly favored an ornithopod trackmaker but did not result in a strong affinity with either dinosaur clade (Table 4).

The final tridactyl track morphology at BP1 is track BP1_06 (Fig 16B). BP1_06 is mesaxonic, tridactyl track with a potentially broad heel and moderately wide toes. Its lateral digits have acted as propagating channels for desiccation cracks. Digit III tapers to a point and preserves the remnants of a probable claw impression. As a result of this impression, BP1_06 can be framed as one of the most well-preserved of the tracks at this site and, although still ranked as 1, teeters on the edge of preservation grade 2 (where tracks preserve clear toe and ungual marks) [39, 40]. The track is 16.4 cm long and 13.7 cm wide. The interdigital angle between the left lateral digit and digit III is 32° and between the right lateral digit and digit III is 29°.

BP1_06 is distinctive from the larger tridactyl tracks observed in TA_1 because of its overall size, the more curved lateral margins of its digits, and because the heel is less elongate (broken). It differs from the tracks of BP1_Twy_02 because the digits are broader and from the isolated track BP1_01 because the digits taper sharply and end in distinctive claw impressions instead of slightly pointed tips. Although the track cannot be assigned to a specific ichnotype because diagnostic characters are lost to the desiccation cracks propagating from both lateral digits and obstruction of the heel by the overlying bed, its presence at BP1 hints at an additional, bipedal trackmaker.

**Fig 17:**
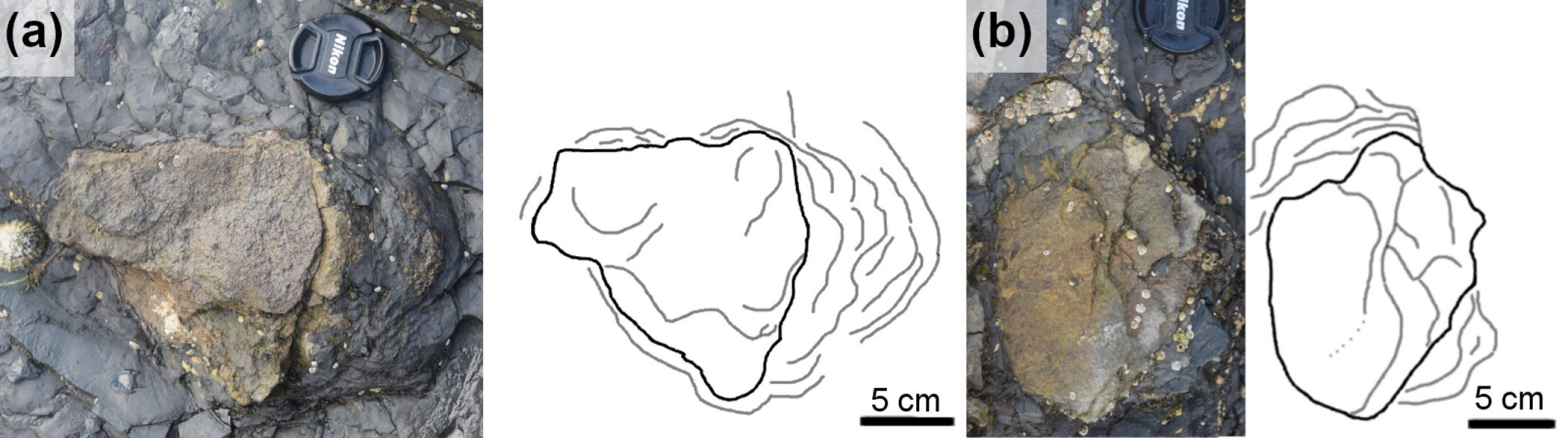
Eroded and in-filled casts observed at BP1. Examples of this track type can be seen with (a) BP1_21 and (b) BP1_22. These eroded casts are particularly abundant around the margins of the track-bearing platform. The track outlines are shown in black while soft-sediment deformation is illustrated in gray.

Several other possibly modified tracks are observed at BP1 and represent eroded, in-filled casts with pronounced soft-sediment deformation around them (Fig 17). These non-diagnostic, broken casts are useful in giving a sense of the relatively high density of tracks on the paleosurface.

### Brothers’ point 3

A variety of track morphologies are preserved at Brothers’ Point 3. A total of 18 tracks are observed at the site and numbered using a system of BP3_01, BP3_02, etc. Overall, two trackways (BP3_Twy_01 and BP3_Twy_02) are observable. A link to the subsampled photogrammetric model of the concentration of large tridactyl tracks at the outcrop can be found in S1 Appendix.

#### Large tridactyl tracks

**Fig 18:**
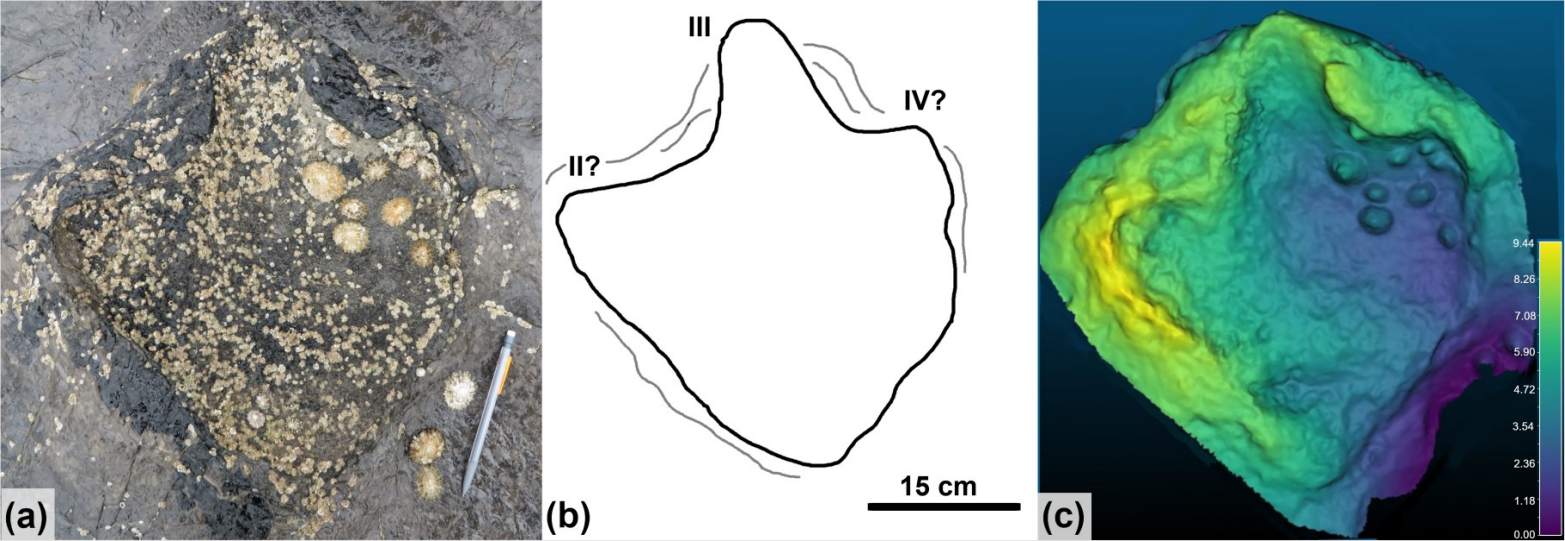
Exemplar large tridactyl track (BP3_08). (a) A field photograph of BP3_08 with (b) an interpretive outline and (c) a false color depth map. This track (black outline) is the most complete and diagnostic presentation of the dominant track morphotype at BP3. The light gray outlines show the areas of most intense sediment deformation. The color scale of the false color depth map is in units of cm.

BP3_08 (Fig 18) is the best preserved and most diagnostic track at the site. It is located along the southern edge of the platform immediately adjacent to BP3_Twy_01. The track forms a deep impression in the shale unit with a pronounced deformation rim around the entirety of the track margin. The digit outlines are particularly affected by the sediment deformation, as the shale layers appear to curl around their outer edges. The track is partially infilled by a thin layer of light tan limestone consistent in character with the overlying bed. The preservation grade [39, 40] of this track is 1 because the marks of the toes are distorted but visible and only the general outline of the track is clearly preserved.

BP3_08 is tridactyl, mesaxonic, and slightly asymmetric (i.e. digits II and IV are slightly different shapes). It is 40.2 cm long and 35 cm wide with an interdigital angle of 38.5° (II^III) and 23.8° between digits (III^IV). Thus, the angle of divarication between the lateral digits (II^IV) is 62.3°. The assignation of digits II and IV is tentative, as this track is not part of a trackway. Since the tracks that compose BP3_Twy_01 exhibit a digit II that is more prominent than digit IV, a similar inference was made for numbering the digits of this isolated track. However, the existence of a slight notch below assumed digit IV might indicate that this is in fact the medial toe [95]. Since ambiguity exists in the siding of this track, the later quantitative analysis is run twice (once with the track sided as a right pes and once as the left).

BP3_08 has a wide, broad heel and short, blunt toes with u-shaped outlines. The width of the heel is wider than the basal width of digit III. The lateral digits are nearly as wide as they are long and the length/width ratio of the track (1.15) demonstrates that these two values are nearly subequal. There is not clear curvature in the digits or evidence of a hallux impression in the smooth posterior margin of the track.

**Fig 19:**
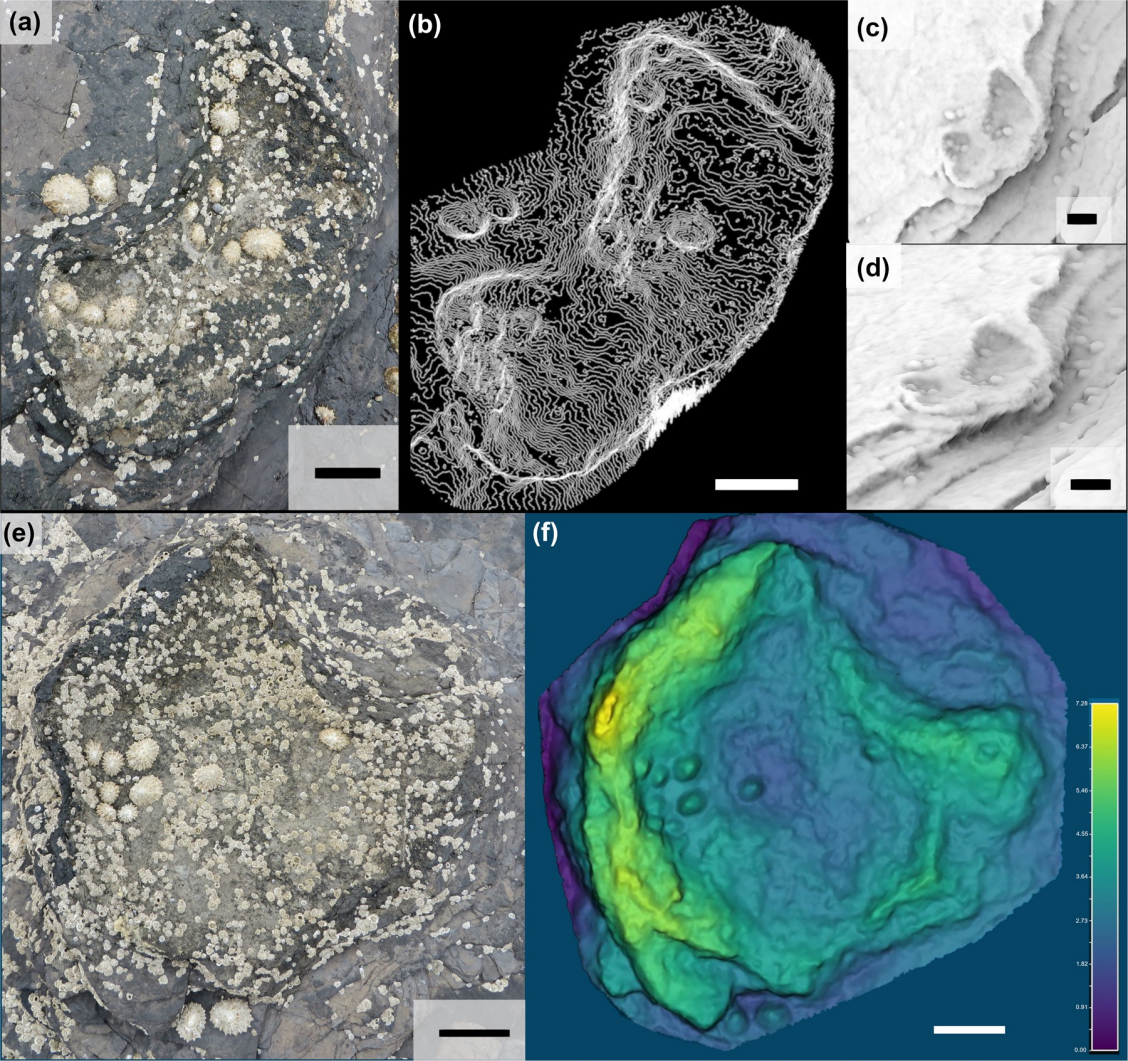
Large tridactyl track preservation at BP3. (a) A field photograph of track BP3_05 illustrating the preservation typical of the outcrop–intense sediment deformation such that the outer margins of the track are affected by curling in the underlying shale layers. BP3_05 is near unique within the tracksite for retaining a minimal amount of in-filling sediment with some present in the digit impressions, but otherwise little in the track. (b) Contoured (1 mm distance between lines) and (c, d) ambient occlusion renderings of the tracksite photogrammetric model highlight the presence of a ridge between the toes of BP3_05. (e) A field photograph and (f) false color depth map of track BP3_01 again illustrating the intense sediment deformation around the margins of the track typical at the site. All scale bars 5 cm.

The majority of other tracks at the site broadly conform to a similar morphology to that of BP3_08 across a varied range of completeness (some tracks are broken along the edges of the platform while others are in-filled and partially obscured by the overlying limestone bed) (Fig 19).

**Fig 20:**
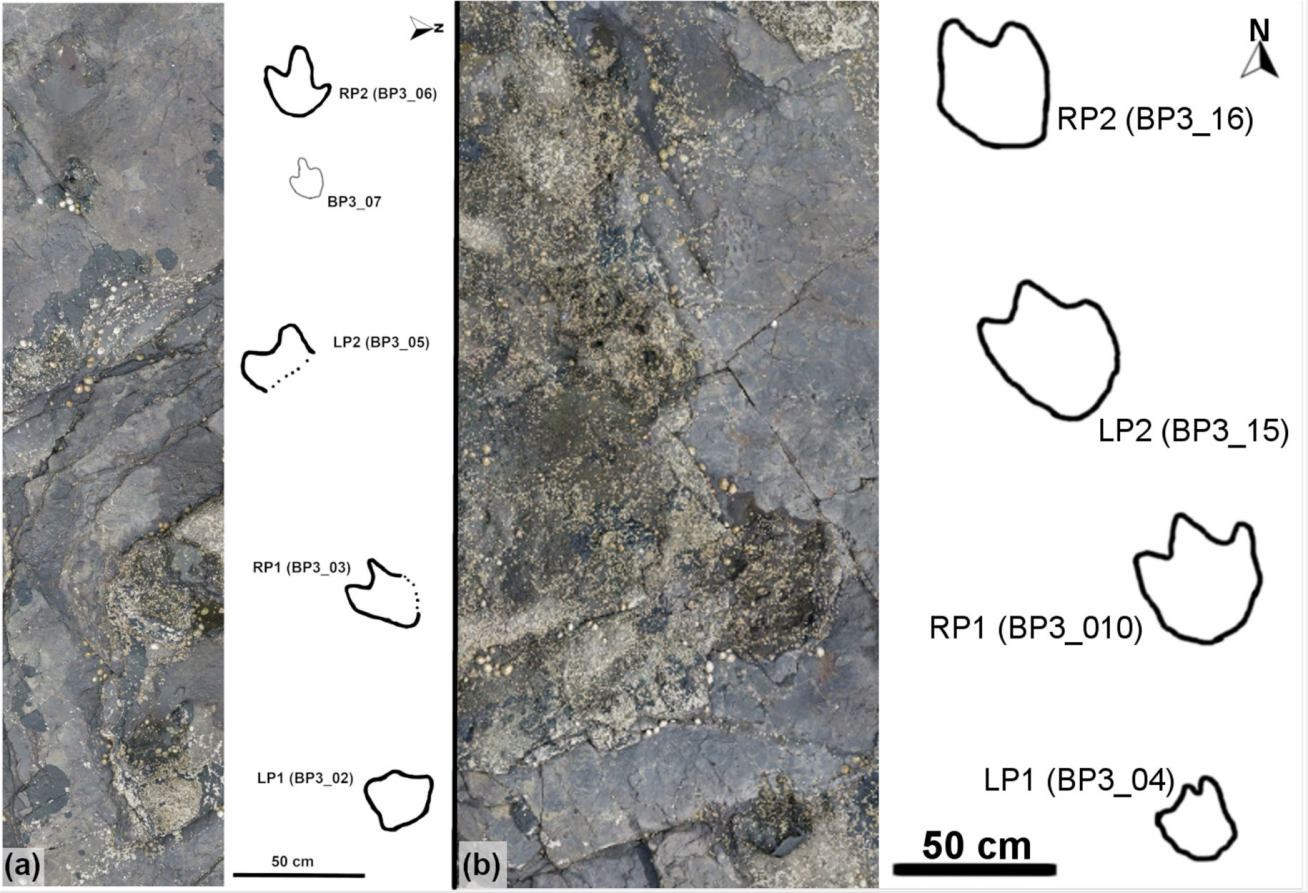
Overview of trackways at BP3. (a) BP1_Twy_01 shown in both the photogrammetric orthophoto and as a line drawing. The trackway consists of four, partially complete, alternating pes impressions where LP denotes the left pes and RP denotes the right pes. LP1 and RP1 are separated from LP2 and RP2 by a break in the track-bearing platform. (b) BP1_Twy_02 shown in both the photogrammetric orthophoto and as an outline drawing. The trackway consists of four pes impressions. LP1 is the most fragmentary track and is only preserved as a deep heel impression. This deep heel persists through the other tracks. The trackway skirts the edge of the overlying beds and, as a result, many of the features of the constituent tracks are obscured.

In addition to BP3_08, two short tridactyl trackways (BP3_Twy_01 and BP3_Twy_02), each consisting of four tracks, are present at BP3. BP3_Twy_01 consists of the large tridactyl tracks BP3_02, BP3_03, BP3_05, and BP3_06. BP3_02 and BP3_05 (the left pes impressions of the trackway) both are truncated by the edge of the track-bearing platform and preserve remnants of the medial and one lateral digit. In both bases, the medial digit is short, blunt, and slightly longer than the lateral digit. The preserved hypices of these tracks form flattened, slightly concave scoops between the digits. Like BP3_02 and BP3_05, BP3_03 preserves the medial digit and the internal lateral digit (II). However, unlike the left pes impressions of the trackway, erosion of the in-filling sediment drape is the mechanism by which the lateral digit was lost. In place of digit IV, there is a broad and shallow impression in the underlying, shale layer. BP3_06 is unique within the BP3_Twy_01 as it does not have the in-filling drape of the overlying limestone that characterizes most of the tracks at this site. It consists of a shallow impression with a pronounced digit III and less distinct digits II and IV. BP3_Twy_01 (Fig 20 and Table 5) is oriented at 263° and is 3.29 meters long. The stride and pace lengths of the trackway were quite variable, with both exhibiting approximately 40 cm variability between the maximum and minimum measurements (Table 5). This variation can be explained, at least in part, by the fragmentary nature of the tracks (particularly the left pes impressions), which makes consistent measurements difficult. The average pace length of the trackway is 1.05 m and the average stride length is 2.04 m. The pace angulation of the trackway is slightly more regular and averages to 147°.

**Table 5:** BP3 trackway measurements.

	N	LP[1]	LP[2]	RP	S[1]	S[2]	WAP[1]	WAP[2]	γ[1]	γ[2]	α[1]	α[2]	Total Length	Orientation
BP3_Twy_01	4	0.81	1.23	1.12	1.84	2.25	0.19	0.49	150	147	60	23	3.29	260
BP3_Twy_02	4	0.72	0.72	0.77	1.41	1.45	0.12	0.16	147	150	26	10	2.70	350

The measurements made on the two trackways at BP3 are summarized here. N represents the number of tracks in the trackway. All measurements are reported in meters except for γ, α, and orientation which are measured in degrees. LP[1] and LP[2] represent the left pace measurements taken between LPx and RPx of the trackways respectively (track designations figured on outline drawings). RP represents the right pace measurements between RP1 and LP2. S[1] and S[2] are the stride lengths between the left feet and the right feet respectively. WAP[1] is the trackway width measured between LP1 and LP2 with RP1 as the focal point while WAP[2] is the trackway width measured between RP1 and RP2 with LP2 as the focal point. γ is the pace angulation with [1] using RP1 as the vertex of the angle and [2] using LP2 as the vertex of the angle. Α measures the track rotation of individual footprints. In this case, α [1] corresponds to LP1 and α [2] corresponds to RP2. The fragmentary nature of the footprints measure meant that this value could not be determined with a great deal of confidence.

BP3_Twy_02 consists of BP3_04, BP3_10, BP3_15, and BP3_16. The primary characteristics of interest for this trackway are unusually deep heel impressions. In fact, the first track in the sequence (BP3_04) is preserved solely as a heel impression with no clear indication of the toes. BP3_10, BP3_15, and BP3_16 all are dominated by the remnants of an in-filling cast of the overlying limestone. These infilling casts hint at the remains of short, broad anteriorly directed digits, but the tracks themselves are, again, characterized by pronounced heel impressions.

Due to only the posterior margin of the BP3_04 being preserved, this track is substantially smaller than the other tracks that compose this trackway. Strangely, although all four tracks are observed on the same bedding plane, there is no hint of an anterior margin and little of the lateral margins of BP3_04. This difference of in the preservation between this track and subsequent ones in the trackway raises the concern that it may not, in fact, belong to the trackway. To combat this concern, all trackway parameters tabulated in Table 5 are calculated individually. However, notably, the stride and pace lengths measures between BP3_04 and the subsequent 3 tracks are the most consistent of any measured from other trackways at these two sites which seems to imply that the trackway relationship between BP3_04 and the subsequent tracks of BP3_Twy_02 remains a reasonable inference.

BP3_Twy_02 (Fig 20B) is oriented at 350° and is 2.70 meters long. The pace and the stride lengths of this trackway are quite regular and average to 0.734 m and 1.43 m respectively. The pace angulation of the trackway is approximately 148°. The preservation grade [39, 40] of the tracks that constitute BP3_Twy_01 ranges from 0 to 1 while the preservation grade of those that constitute BP3_Twy_02 is more consistently 0.

BP3_Twy_01’s trackmaker hip height was calculated using the average length of the complete tracks. The average trackway stride length was 2.04 m while the relative stride length is less than 2.0. Similarly, the smaller stride length of BP1_Twy_02 (1.43 m) and the average of the two most complete tracks (BP3_10 and BP3_15) yield an even lower stride length value. These values indicate that the trackmakers were both moving at a walking pace. We summarize the approximate hip heights of the trackmaking individuals and their estimated velocities in Table 6.

**Table 6:** BP3 large bipedal trackmaker size and velocity estimations.

	Average Approximate Trackmaker Hip Height (cm)	Estimated Velocity	
		m/s	km/hr
BP3_Twy_1	128	1.9	7.0
BP3_Twy_2	169	0.77	2.8

The range in approximate trackmaker sizes at BP3 indicates that at least two differently sized individuals were present on the mudflat. BP3_Twy_02’s trackmaker represents the largest individual at the site and was moving at the slowest estimated velocity.

Due to the poor quality of preservation of BP3_08 and the other large tridactyl tracks at the site, it is not possible to confidently assign them to a specific ichnotaxon. Since the track lengths on average are greater than 30 cm, they fall into the large, bipedal dinosaur size category. Two different trackmaker inferences are possible for the dinosaurian trackmaker of BP3_08 –theropod or ornithopod.

A theropod trackmaker could potentially be inferred for the large tridactyl tracks at BP3 because these footprints are generally longer than they are wide which results in a narrower aspect ratio [63]. Additionally, the tracks at BP3 often present with asymmetry across the track midline with several tracks showing a disparity of ~10° between the (II^III) and (III^IV). Asymmetry in tridactyl tracks is more strongly associated with theropod footprints (and can be employed to assist in siding isolated pes impressions) [95] while ornithopod footprints are considered more likely to be subsymmetrical [43, 66].

An ornithopod trackmaker could potentially be inferred for BP3_08 on the basis of the track's wide, broad heel with a smooth posterior margin and its short, blunt toes with u-shaped outlines [38, 96]. The tracks lack clear curvature in the digits and evidence of claw marks or hallux impressions [96]. Additionally, the angle of divarication between the lateral digits is (II^IV) is 62.3° which is generally congruent with the angle of 60°-65° postulated for ornithopod trackmakers [38, 96]. In terms of trackway characteristics, the slight (inward) rotation of the pes in BP3_Twy_01 provides another line of evidence for a potential ornithopod trackmaker.

This trackmaker inference is further supported by the statistical parameters derived by [42], but these measurements must be regarded with caution because they are affected by the presence of infilling sediment. Sediment infilling tends result in a smoothing of the track outline and can result in tracks showing more ‘ornithopod-like’ features, like shorter toes and longer distances between the posterior margin of the track and the hypex. In the case of BP3_08, the measurements of the hypices, in particular, are likely affected by the presence of infilling sediment. Table 7 summarizes the statistical parameters. For each ratio presented, BP3_08 exhibits more ornithopod affinities.

**Table 7:** Assessment of the trackmaker affinity of BP3_08.

IF BP3_08 IS A RIGHT PES		
Track Parameters	Threshold values and probability that the track is either theropod or ornithopod	BP3_08
L/W	80.0% Theropod > 1.25 > Ornithopod 88.2%	1.15
L/K	70.5% Theropod > 2.00 > Ornithopod 88.0%	1.10
L/M	65.0% Theropod > 2.00 > Ornithopod 90.7%	1.29
BL2/WMII	76.1% Theropod > 2.00 > Ornithopod 97.4%	1.04
BL3/WMIII	72.7% Theropod > 2.20 > Ornithopod 97.7%	1.42
BL4/WMIV	76.1% Theropod > 2.00 > Ornithopod 97.6%	1.09
LII/WBII	84.6% Theropod > 3.75 > Ornithopod 90.2%	2.88
LIII/WBIII	70.6% Theropod > 4.00 > Ornithopod 91.5%	3.79
LIV/WBIV	73.7% Theropod > 3.75 > Ornithopod 93.4%	3.35
IF BP3_08 IS A LEFT PES		
Track Parameters	Threshold values and probability that the track is either theropod or ornithopod	BP3_08
L/W	80.0% Theropod > 1.25 > Ornithopod 88.2%	1.15
L/K	70.5% Theropod > 2.00 > Ornithopod 88.0%	1.29
L/M	65.0% Theropod > 2.00 > Ornithopod 90.7%	1.10
BL2/WMII	76.1% Theropod > 2.00 > Ornithopod 97.4%	1.09
BL3/WMIII	72.7% Theropod > 2.20 > Ornithopod 97.7%	1.42
BL4/WMIV	76.1% Theropod > 2.00 > Ornithopod 97.6%	1.04
LII/WBII	84.6% Theropod > 3.75 > Ornithopod 90.2%	3.35
LIII/WBIII	70.6% Theropod > 4.00 > Ornithopod 91.5%	3.79
LIV/WBIV	73.7% Theropod > 3.75 > Ornithopod 93.4%	3.35

The calculated track parameters for BP3_08 are summarized and compared with the likelihood analyses of [42]. Since the siding of this pes impression was equivocal, the analysis was run twice (once assuming the foot was a right pes and once with the assumption of it being a left pes. The analysis yielded the same results in both cases because the parameters of [42] propose identical thresholds for values that are symmetric across the midline (albeit with different confidences). In this case, the observed asymmetry of the track does not have a notable influence on the resultant affinity. For each parameter examined, BP3_08 shows a stronger ornithopod affinity than a theropod affinity.

Although the identity of the trackmaker for BP3_08 cannot be resolved with confidence, if this track was made by an ornithopod trackmaker, some interesting implications arise that merit further discussion (see ‘Implications of Potential Large Ornithopod Tracks’ section below).

Although the outcrop at BP3 is dominated by large tridactyl tracks, the track assemblage at this site is not monotaxic. A second, noteworthy track morphology (small, tridactyl) is visible through a window in the overlying bioclastic limestone.

#### Small tridactyl track

**Fig 21:**
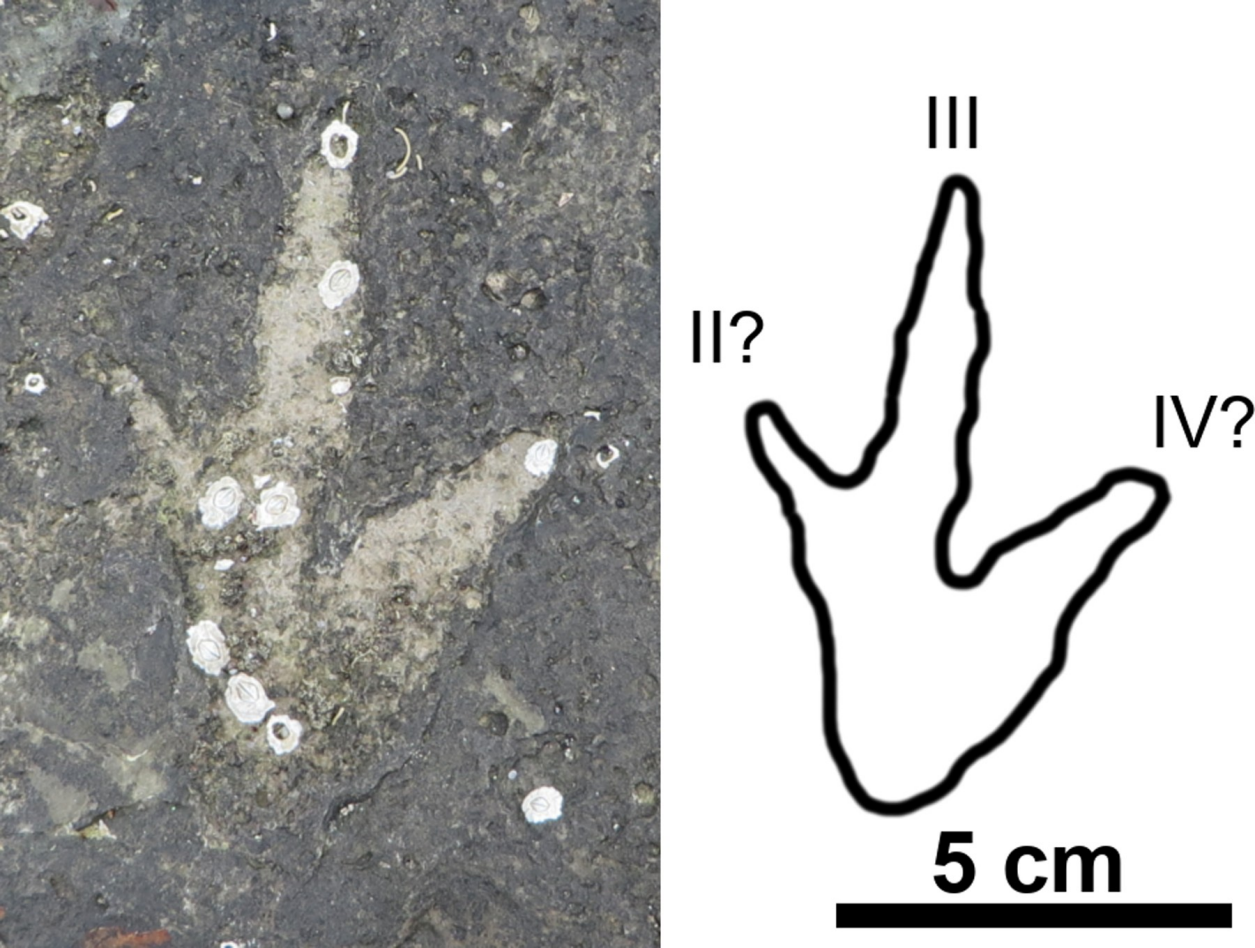
Small tridactyl track from BP3 (BP3_13). BP3_13 is shown with both a field photo and a line drawing. It is the smallest track at BP3. This narrow-toed track morphology–typical of Triassic and Early Jurassic tracks–is unique with regard to all other tracks at BP1 and BP3.

Track BP3_13 (Fig 21) is a small track located west of Trackway 2, east of the small tidal pool shown on the site map with a dashed outline, and north of two large isolated boulders in the south-west corner of the platform (Fig 5). The orientation of the track's elongation axis is almost directly north. This track is not associated with the large concentration of tracks along the south edge of the platform. The track is preserved as a shallow impression in the uppermost layer of the track-bearing shale unit. The presence of a dark, biotically-derived (algal?) coating on most of the rock surface makes interpretation of this layer and the subtle features of the footprint within it difficult to interpret. The preservation grade [39, 40] of this track is 1 because the outlines of the digits are clear, but there are no clear indications of ungual marks or digital pads.

BP3_13 is tridactyl and mesaxonic, with elongate narrow toes ending in sharp points and a narrow ‘heel’ with a ‘v-shaped’ posterior margin. No distinctive claw impressions were observed. There is no evidence of a hallux impression. The track is 8.9 cm long and 5.1 cm wide with interdigital angles of 27° between digit IV? (tentatively assigned to the right digit) and digit III and 21° between digit III and digit II? (tentatively assigned to the left digit). This track plots as having a theropod affinity according to the statistical parameters from [41] (Table 8).

**Table 8:** Assessment of the trackmaker affinity of BP3_13.

Track Parameters	Threshold values and probability that the track is either theropod or ornithopod	BP3_13
L/W	80.0% Theropod > 1.25 > Ornithopod 88.2%	1.75
L/K	70.5% Theropod > 2.00 > Ornithopod 88.0%	1.93
L/M	65.0% Theropod > 2.00 > Ornithopod 90.7%	2.62
BL2/WMII	76.1% Theropod > 2.00 > Ornithopod 97.4%	2.33
BL3/WMIII	72.7% Theropod > 2.20 > Ornithopod 97.7%	4.30
BL4/WMIV	76.1% Theropod > 2.00 > Ornithopod 97.6%	3.11
LII/WBII	84.6% Theropod > 3.75 > Ornithopod 90.2%	4.55
LIII/WBIII	70.6% Theropod > 4.00 > Ornithopod 91.5%	6.85
LIV/WBIV	73.7% Theropod > 3.75 > Ornithopod 93.4%	3.12

The calculated track parameters for BP3_13 are compared with the likelihood analyses of [42]. For the majority of the parameters examined, BP3_13 is more likely a theropod track than an ornithopod track. The track shows a slight ornithopod affinity with regard to two parameters: LIV/WBIV and L/K. The first inconsistency occurs because the width at the base of digit IV is relatively large. This result is because of a slight outward bend along the right edge of the track. It is possible that this feature results from the eye being tricked by the growth of the dark coating on the limestone. Similarly, the L/K ratio examines the hypex distance between the digits III and II. As digit II appears to truncate into digit III quite early, the length of the hypex distance is increased. As with LIV/WBIV, it is possible that the perception of this track parameter may have been affected by the eye being drawn to areas of sharp contrast (e.g. between light and dark colors) rather than to the correct track margins.

The short length of this footprint places it in the tiny bipedal trackmaker size class [44] and the estimated hip height of the trackmaker is ~36 cm. BP3_13 is the smallest track documented across both BP1 and BP3.

## Discussion

BP1 and BP3 preserve tracks from a variety of dinosaurian trackmakers, including bipedal theropods, possible ornithopods, and a quadrupedal ornithischian. The only major dinosaur clade that is not represented at the site is Sauropoda. The lack of sauropod tracks at these sites may be environmental as the sauropod tracks previously described from Skye [24, 25] were made in shallow lagoons and not on subaerially exposed mudflats (the depositional environments inferred for the BP1 and BP3 sites). Although sauropod tracks are not present at BP1 and BP3, the track morphologies observed at these sites (which are located in close stratigraphic and geographic proximity to one another) illustrate a diverse Middle Jurassic dinosaur fauna for Skye. These sites are currently the single best glimpse into the diversity of Skye’s Middle Jurassic ecosystems because other tracksites on the island exhibit a much lower trackmaker diversity ([21] and references therein). Indeed, the desiccation surfaces on which both of these tracksites formed were likely only exposed for short periods of time before being reclaimed by the marginal lagoons. Thus, this wide diversity of dinosaurs is recorded as living more or less contemporaneously in the same environments. Although behavioral interpretations of the animals are limited by the small surface area of the outcrops and the low number of trackways present, the range of trackway orientations and lack of a preferential direction of movement on these admittedly limited exposures more strongly hints at either the time averaging of multiple generations of tracks at the site or milling behavior rather than any obvious predator-prey, migratory, or other complex behaviors. Essentially, BP1 and BP3 give us a snapshot of a ‘day in the life’ of a rare Middle Jurassic ecosystem.

### Implications of *Deltapodus* tracks

**Fig 22:**
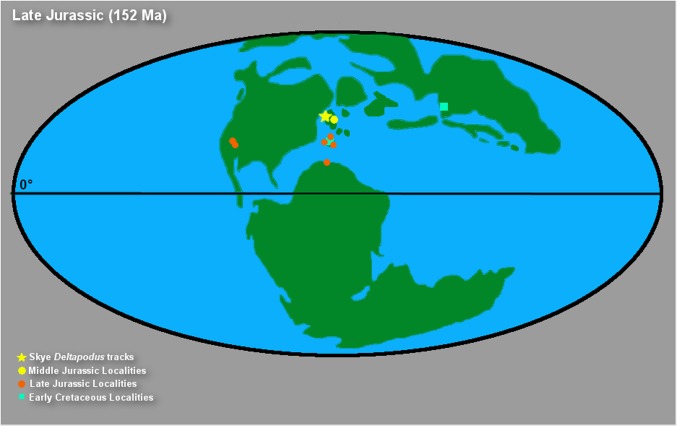
Global distribution of *Deltapodus* tracks. A paleogeographic reconstruction showing the global distribution of *Deltapodus* tracks (localities from [56, 81–84, 86, 97, 98]). The majority of reported *Deltapodus* tracks are concentrated in western Europe, primarily in Iberia and the UK, while sparser groupings exist in the western United States, China, and northern Africa. The yellow, orange, and teal symbols indicate Middle Jurassic-aged, Late Jurassic-aged, and Early Cretaceous-aged localities respectively. The Skye *Deltapodus* tracks represent the most northerly occurrence of the ichnogenus. Paleomap redrafted from [99].

The *Deltapodus sp*. tracks at BP1 are the first reported examples of the ichnogenus from Skye and are among the oldest examples of this ichnotaxon from anywhere in the world. To our knowledge, the only reported *Deltapodus* tracks that are definitively older come from the Aalenian-aged type series in Yorkshire [74, 85]. All other *Deltapodus* tracks have been described from Late Jurassic to Early Cretaceous-aged sediments [56, 81–84, 86, 97, 98]. Additionally, the Skye *Deltapodus sp*. tracks are the most northerly occurrence of this ichnogenus and thus extend its geographic range (Fig 22). Therefore, the Skye tracks give important insight into the origin and early evolution of the *Deltapodus* trackmaker, widely regarded to be a thyreophoran ornithischian and most likely a stegosaur [84].

It is generally held that *Deltapodus* tracks were produced by a stegosaur, a member of the subgroup of plate-backed, beaked, plant-eating dinosaurs that includes the iconic Late Jurassic *Stegosaurus* [84]. However, there is some debate about this assignment, as other workers have proposed that the trackmaker was an ankylosaur [76, 78]. Theis alternative hypothesis has been based on postulated trackway characteristics (i.e. that the rotation of the pes more accurately represents ankylosaurs) and because of the shortness and bluntness of the toes which are held to be different from the more elongate toes of stegosaurs [78]. Additionally, [76] argued that presence of another aptly named ichnogenus, *Stegopodus*, with longer, more developed digits was more representative of the track morphology implied from the osteology of the stegosaurian manus and pes. They concluded that another trackmaker must, therefore, be sought for *Deltapodus* tracks.

Despite these challenges, we find the argument of stegosaurian affinity for *Deltapodus* to be most persuasive based on the number of digits in the pes. Although the ankylosaur pes has ranged from tetradactyl to tridactyl through the history of the clade, tridactyly is currently known only in the Cretaceous genera *Euoplocephalus* [100], *Pinacosaurus* [101], and *Liaoningosaurus* [102]. Most ankylosaurs, for instance, *Sauropelta* [103], have four pedal digits. Similarly, the basal thyreophorans that are outgroups to Eurypoda (Ankylosauridae + Stegosauridae), *Scutellosaurus* and *Scelidosaurus*, have a tetradactyl pes (phalangeal formulae summarized in [104]), further suggesting this morphology is primitive for ankylosaurs and would be expected in the earliest and most basal members of the group. Indeed, the basal ornithischian, *Lesothosaurus*, which has been found as the most basal member of Thyreophora in some phylogenies [105, 106] also has a tetradactyl pes [107]. Thus, a Middle Jurassic-aged tridactyl ankylosaurian trackmaker seems unlikely. In contrast, a tridactyl pes is broadly observed among stegosaurs, with genera like *Kentrosaurus* [76, 108] and *Stegosaurus* [109] possessing this characteristic.

Biomechanical analyses of the postural differences between ankylosaurs and stegosaurs also support a stegosaurian affinity for *Deltapodus*. Stegosaurs, like ceratopsids, appear to have held their forelimbs in a flexed stance with the elbows abducted from the parasagittal plane [110]. This stance would have loaded the medial side of the manus [111]. In contrast, the elbows of ankylosaurs were held parallel to the parasagittal plane and, thus, they were more likely to evenly distribute their weight across all the digits of the manus [111, 112]. *Deltapodus* manus prints from Yorkshire often preserve a medial projection of the pollex [74]. Similarly, the clearest *Deltapodus* manus impression from Skye shows a smooth anterior margin and the subtle hint of a pollex on the medial margin (Fig 7). The preservation of the pollex in the *Deltapodus* manus as that of a more pronounced impression than the other digits could indicate that the trackmaking individual was loading medial portion of the manus more than the lateral digits. Indeed, [112] recently claimed that *Deltapodus* tracks “agree with the foot skeleton and anticipated footprints of a quadrupedal stegosaur such as *Stegosaurus* and not with those of other potentially contemporaneous dinosaurs.”

The strongest ichnological critique of *Deltapodus* tracks possibly not having a stegosaurian origin came from the contrast of these tracks with *Stegopodus* prints, which are widely considered to have been made by stegosaurs [76]. However, recent discoveries from China hint that some of the differences between these ichnotypes may be due to the consistency of the substrate and subsequent preservation [113]. The variable preservation of *Shemuichnus* (an early Jurassic ichnogenus attributed to ornithischians of unknown affinity) from shallow impressions that strongly resemble *Stegopodus* to deep tracks whose morphology is more consistent with *Deltapodus* has the broader implications that *Deltapodus* tracks may simply be 'deep tracks' from the same trackmaker as *Stegopodus* [84, 113]. This realization has the potential to resolve much of the apparent dichotomy between the ichnogenera. Thus, in summary, a consensus is slowly being reached between both ichnologists and osteologists regarding a likely stegosaurian affinity for *Deltapodus*.

The likely stegosaurian identity for *Deltapodus* and the identification of *Deltapodus* tracks on Skye have important implications for our understanding of Middle Jurassic dinosaur faunas and stegosaurian evolution. The *Deltapodus sp*. tracks at BP1 are the trace fossil evidence of possible stegosaurs from Skye, and the first strong indication that these dinosaurs were part of the diverse Middle Jurassic fauna of the island. A single osteological discovery–a partial ulna and radius from the Bearreraig Sandstone (a nearshore marine unit that underlies the Lealt Shale Formation)–hinted that a thyreophoran may have been present on Skye, but the fragmentary nature of these bones precluded a precise identification [9]. The bones lacked any specific diagnostic features of stegosaurs. The *Deltapodus* tracks corroborate the presence of a thyreophoran on Skye using an independent line of evidence and, specifically, point to a stegosaur.

Additional evidence of stegosaurs from the Middle Jurassic can be found with the *Deltapodus* tracks from the Aalenian in Yorkshire and body fossils including the partial skeleton of *Loricatosaurus* from the Callovian Oxford Clay Formation, vertebrae and a humerus of *Adratiklit boulahfa* from the of Bathonian of Morocco, isolated vertebrae and dermal plates from the UK referred to Stegosauria indet. and dorsal vertebrae from Kyrgyzstan referred to Stegosauria indet. [74, 112, 114–117]. Additionally, the Bajocian-aged ornithischian *Issaberrysaura mollensis* has been found as a stegosaur in recent phylogenies [118–120]. The oldest definitive ankylosaur, *Sarcolestes leedsi* is also from the Oxford Clay [121]. The presence of body fossils from Ankylosauridae (the sister group to Stegosauridae) further demonstrates that these groups had clearly diverged by the Middle Jurassic. In conjunction with these fossils, the Skye *Deltapodus* tracks suggest that stegosaurs had evolved by the Aalenian-Bajocian at latest and were already present in European and Asian ecosystems during the Middle Jurassic, before becoming larger in size and more geographically widespread later in the Jurassic.

### Implications of possible large ornithopod tracks

The large tridactyl tracks found at BP3 range in size from ~30-~40 cm, with the corresponding hip heights for the trackmakers falling between an estimated 123 and 160 cm. Notably, we did not observe any clear manus impressions in the concentration of tracks at BP3. Indeed, BP3_Twy_01 at this site clearly shows an alternating series of deep pes impressions only. We find no clear taphonomic reason (resulting from post-registrational processes) for manus track to be missing from this trackway if the animal was in actuality walking quadrupedally. However, the manus impressions of ornithopod tracks can often be shallow and weakly impressed into the rocks so it is possible that a bias against these features being registered in the substrate to similar depths and extents as the deep pes impressions. Based on their track size and characteristics, the animals that made these tracks might potentially have been large ornithopods that could have been at least functionally (facultatively) bipedal.

Large ornithopod footprints are not unknown from the Middle Jurassic of Skye. Indeed, the first dinosaur footprint discovered from Skye (GLAHM V1980) was derived from a layer of the Lonfearn Member of the Lealt Shale Formation very close in the stratigraphy to BP3 [22]. Although initially postulated to be a theropod track, it was later reinterpreted as being made by a large ornithopod trackmaker [22, 122]. Reexamination of GLAHM V1980 in context of this discussion proves useful because this isolated positive relief cast shows several qualitative features consistent with an ornithopod affinity. GLAHM V1980 is 45 cm long, 55 cm wide, and has wide interdigital angles (35° and 44°). It has a broad aspect ratio (L/W = 0.82) and the presence of low, weakly developed ridges partitioning between the toes and heel. These ridges are most clearly observed in the left lateral and central digits (cf. Fig 3A of [16]). The ornithopod affinity of GLAHM v1980 seems more clearly supported than that of the *in-situ* footprints at BP3 and provides another line of evidence for the potential of large ornithopods in the Middle Jurassic of Scotland.

GLAHM V1980 was attributed to a *Camptosaurus-*like trackmaker [122] and similar-shaped footprints that reached about 70 cm in length from the Late Jurassic of Portugal were also attributed to a *Camptosaurus*-like trackmaker [64]. *Camptosaurus* is recognized as one of the most basal members of the ornithopod clade Ankylopollexia whose oldest members are known from Late Jurassic rocks [105–107].

The story told by the Skye tracks is challenging because large ornithopod footprints are predominantly found in Cretaceous-aged strata and generally are not postulated to extend much earlier in time than the Late Jurassic [43, 94]. Additionally, body fossils of the most commonly suggested trackmaker for this particular track morphology–ornithopods of similar size and phylogenetic affinity to *Camptosaurus*—are not known from the Middle Jurassic. Insight cannot be drawn from other fossils found on Skye because, in contrast to the fragmentary remains known from sauropods, theropods, and thyreophorans, no definitive ornithopod body fossils are yet known from the island.

Although scarce, Middle Jurassic-aged ornithopod body fossils are known from elsewhere in the UK, most notably a femur from the Oxford Clay of Peterborough assigned to *Callovosaurus leedsi*, which is interpreted to be a dryosaurid [123, 124]. Although dryosaurids generally had a small to medium body size (2–3 m long; [125]), it is possible that the trackmaker of GLAHM V1980 could be a large member of this clade. Although less definitive, the large tridactyl footprints at BP3 might also correspond to feet of these small-medium bodied ornithopods as the pes of these animals is narrow [126]. Alternatively, recent phylogenies of basal ornithopods recover Ankylopollexia as a sister group to Dryosauridae [105, 106, 124]. Based on these relationships, fossil evidence of dryosaurids from the Middle Jurassic implies that an ankylopollexian ghost lineage extends back into this time. Thus, the ornithopod tracks found on Skye could have been made by a smaller, primitive member of the generally large-bodied ankylopollexian clade–a ghost taxon, unrecorded by body fossils, known only from its traces.

In summary, we suggest that the trackmaker for Skye’s large ornithopod tracks (if, indeed, the tridactyl footprints at BP3 and GLAHM V1980 are such) belongs to the clade Dryomorpha (the Dryosauridae + Ankylopollexian clade). It could either be a larger member of Dryosauridae or, alternatively, a small, basal ankylopollexian (*sensu* [106]). In general, ‘ornithopod’-type footprints from the Early—Middle Jurassic are small (12–20 cm) [127–130] with some compelling medium to large prints known from Yorkshire [85, 131]. The ornithopod tracks found on Skye are some of the earliest potential large ornithopod footprints known from anywhere in the world, providing additional evidence for the existence of fairly large-bodied ornithopods in the Middle Jurassic and augmenting the sparse body fossil record for this time interval.

We reiterate, however, that the difficulties in distinguishing theropod and ornithopod tridactyl trackmakers means we cannot yet be fully confident that ornithopods lived on Jurassic Skye. Future body fossil discoveries will be the best test of the ornithopod trackmaker hypothesis.

### Variations in theropod size range

Between BP1 and BP3, tridactyl tracks attributed to theropods range from the tiny size class (PL < 10 cm) to the large size class (PL ≥ 30 cm) are described (Tables 3 and 5, S2). Conservatively, we can interpret that at least two trackmakers traversed these sites based on the size variation within the tracks. The theropods from BP1 group into medium and large bipedal dinosaur size classes with the approximate hip heights of the bipedal trackway trackmakers ranging from 116–155 cm (Table 3). In contrast, the estimated hip height for the narrow-toed trackmaker of BP3_13 (35.6 cm) is much smaller and falls into the 'tiny' bipedal dinosaur size class. The theropod track from the site implies significantly smaller (~3 to 4 x smaller) trackmaker than the far larger tridactyl tracks located adjacently.

The substantial variation in the size of the theropod tracks at BP1 and BP3 indicates that multiple individuals left their traces on Skye’s Middle Jurassic mudflats. However, identifying these trackmakers beyond the general category of ‘theropod’ is difficult given that there are few major osteological differences among the feet of theropods that would clearly register in tracks. Similarly varying assemblages of theropod ichnotaxa are observed in other Mesozoic tidal flats [132].

### Interpretations of dinosaur behavior

**Fig 23:**
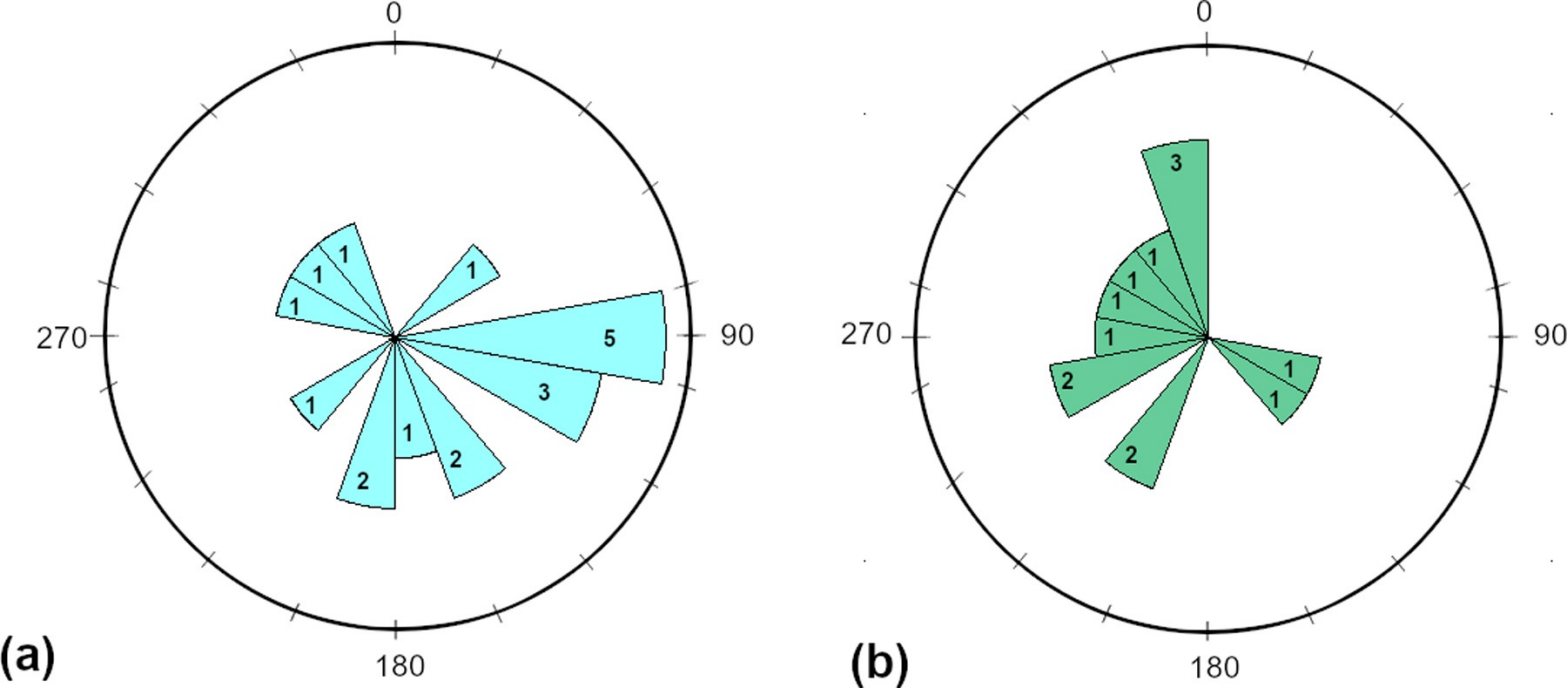
Rose diagrams of track orientations for at BP1 and BP3. Rose diagrams show the orientation of the central axis of all footprints for which this parameter could be measured from (a) BP1 and (b) BP3. These diagrams illustrate that there was no preferred direction of movement among the dinosaurs at either tracksite. The total number of tracks tabulated for BP1 was 18 and for BP3 was 13.

Although the presence of multiple trackways made by individuals of varying sizes shows that multiple dinosaurs traversed these mudflats, the wide variation in orientation of the trackways at both BP1 and BP3 (Fig 23) is more indicative of either different track generations (where individuals traverse the same space asynchronously and do not interact in time) or solitary behavior (milling) than any social or herd behavior (where multiple individuals travel together in a coherent manner) [133]. The trackways and isolated tracks seem randomly scattered across the outcrops and no evidence of gregariousness or interspecific interaction is observed. Speed estimations of the trackways provide slow (walking pace) velocity estimates. While the area of these outcrops, the number of trackways present, and the length of the trackways are too small to allow for confident interpretations, the trackmakers do not seem to be strongly constrained either along a coastline or by some other topographic feature.

### Insights into middle Jurassic dinosaur diversity on Skye

In the context of previously discovered dinosaur tracksites from Skye, BP1 and BP3 are remarkable for their lateral extent, the comparatively high number of tracks, and the presence of a novel ichnotaxon for the Great Estuarine Group (*Deltapodus*). The main contrast between the tracks described from BP1 and BP3 and previous discoveries lies in the novelty of the *Deltapodus sp*. tracks. Stegosaurian tracks have not been previously recognized from Skye. Additionally, the potential large ornithopod tracks provide a clearer understanding of these animals as fairly large-bodied and possibly facultatively bipedal–biological traits that are not evident from the single ornithopod footprint from Skye previously described in the literature [22, 122].

The range in size, morphology and preservation of the theropod tracks at these sites is generally consistent with previously discovered tracksites on Skye [23, 134–137]. Additionally, the subaerial mudflat where these tracks were formed generally corresponds with the depositional environments of these earlier track discoveries.

Notably, BP1 and BP3 lack any evidence of sauropod tracks. However, a recently described tracksite from slightly lower in the stratigraphy at Brothers’ Point is dominated by them [25] as is another tracksite from the slightly younger Duntulm Formation [24]. When we consider the three geographically and temporally close tracksites at Brothers’ Point together, we see representatives from all major dinosaur clades (theropods, sauropods, and ornithiscians).

**Fig 24:**
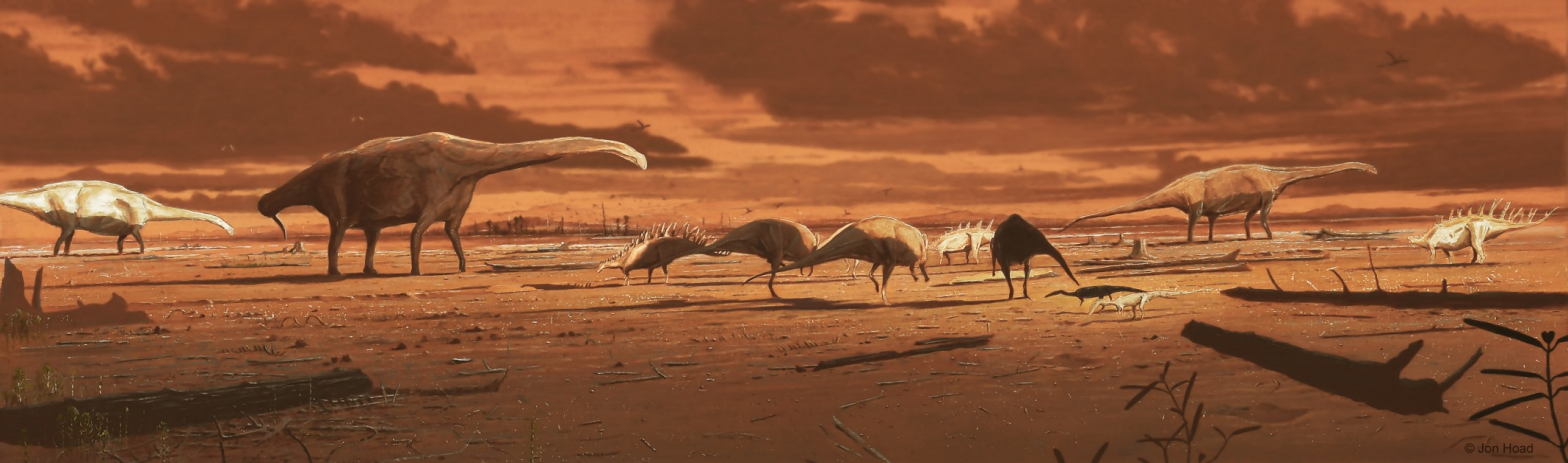
Reconstruction of Middle Jurassic ecosystem on the Isle of Skye, Scotland. A snapshot of what the dynamic coastal environment of Skye may have looked like during the Middle Jurassic which bipedal ornithopods, theropods of various sizes, and stegosaurs in the foreground and middle on subaerially-exposed mudflats. In the distance, large sauropods wade in shallow lagoons. Paleoartist: Jon Hoad.

Dinosaur tracks provide a useful complement to the body fossil record. The body fossil record on the Isle of Skye is sparse and in-situ tracksites allow better inferences to be made about the diversity, abundance, and paleobiology of dinosaurs on Skye. Tracks from Skye hint that sauropods, potentially three size classes of theropod, fairly large probable ornithopods, and possible small stegosaurs all existed relatively contemporaneously in this Middle Jurassic coastal margin environment [21] (Fig 24).

The sauropod and theropod trace fossil records of the island lend weight to the fragmentary body fossil record of these groups [8, 10–14]. Now, the *Deltapodus* sp. tracks at BP1 similarly corroborate the thyreophoran bone record [9]. However, no distinctive ornithopod skeletal material has yet been described from Skye. Thus, the Skye tracks provide evidence of a greater diversity of dinosaurs in this area during the Middle Jurassic than body fossils in isolation. As a result of this diversity, we can infer that a thriving community of dinosaurs lived in and near the subtropical lagoons of Middle Jurassic Scotland.

## Conclusion

We report ca. 50 dinosaur tracks from two new tracksites (BP1 and BP3) at Brothers’ Point on the Isle of Skye. Both sites span two distinctive lithologies–a fine-grained shale and an overlying bioclastic limestone. At both sites, the preservation of the tracks as impressions in the shale layer with desiccation cracks propagating from the toes indicates that the track-bearing surface was subaerially exposed when the dinosaurs were walking on it. The bioclastic limestones indicate that the track-bearing surfaces were resubmerged in a lagoonal environment after desiccation. Thus, these sites show evidence of a dynamic nearshore to coastal depositional environment. Although they cover a relatively small geographic area, BP1 and BP3 show a relatively high degree of dinosaur diversity, with tracks formed by theropod, possible ornithopod, and possible stegosaurian trackmakers present.

In addition to its series of poorly preserved, large tridactyl tracks, BP3 offers a striking testament to the utility of revisiting areas that have been previously prospected, especially dynamic environments like the northern coastlines of Skye. Although this part of the coast of Port Earlish between Valtos and Brothers’ Point is a popular place for tourists to walk and has been measured in detail by stratigraphers and prospected by paleontologists for almost half a century, the tracksite was not recognized until the spring storms of 2017 moved the boulders along the beach to more opportune resting places.

## Supporting information

S1 Appendix Additional method details, intervalometer design, model construction, and comprehensive track measurements. This file is formatted as a .pdf and includes additional information about track measurements and description, the design of the intervalometer, a standardized workflow for constructing photogrammetric models of tracksites, and the comprehensive listing of all track measurements from BP1 and BP3. It also contains information about accessing the photogrammetric datasets associated with the project (reposited in Dryad).
(PDF)

## Institutional abbreviations

GLAHM: The Hunterian, University of Glasgow, Glasgow, Scotland, UK
UCMP: University of California Museum of Paleontology, Berkeley, California, USA
