# Supplementary material for: Novel track morphotypes from new tracksites indicate increased Middle Jurassic dinosaur diversity on the Isle of Skye, Scotland
Source: PLoS One. 2020 Mar 11;15(3):e0229640. doi: 10.1371/journal.pone.0229640 (PMC7065758; doi:10.1371/journal.pone.0229640)
Supplement: S1 Appendix — This file is formatted as a .pdf and includes additional information about track measurements and description, the design of the intervalometer, a standardized workflow for constructing photogrammetric models of tracksites, and the comprehensive listing of all track measurements from BP1 and BP3. It also contains information about accessing the photogrammetric datasets associated with the project (reposited in Dryad). (PDF) [file pone.0229640.s001.pdf]

Supporting Information

to

Novel track morphotypes from new tracksites indicate increased Middle Jurassic dinosaur diversity on the Isle of Skye, Scotland

Paige E. dePolo[1*], Stephen L. Brusatte[1], Thomas J. Challands[1¶], Davide Foffa[2¶], Mark Wilkinson[1¶], Neil D. L. Clark[3&], Jon Hoad[4&], Paulo Victor Luiz Gomes da Costa Pereira[5&], Dugald A. Ross[6&], Thomas J. Wade[1&]

[1]School of GeoSciences, University of Edinburgh, Edinburgh, Scotland, United Kingdom
[2]National Museum Scotland, Edinburgh, Scotland, United Kingdom
[3]The Hunterian Museum, University of Glasgow, Glasgow, Scotland, United Kingdom
[4]Art of Ancient Life Limited, Perth, Scotland, United Kingdom
[5]Departamento de Geologia, Universidade Federal do Rio de Janeiro, Rio de Janeiro, Brazil
[6]Staffin Museum, Staffin, Isle of Skye, Scotland, United Kingdom

* Corresponding author
Email: paige.depolo@ed.ac.uk

¶These authors contributed equally to this work.
&These authors also contributed equally to this work.

Additional Details of Track Descriptions and Measurements

The preservation quality of dinosaur footprints can influence what features are visible with them and should nuance resulting ichnotaxonomic interpretations. We used the preservation grade criteria of [1, 2] to evaluate the quality of each footprint at the site (Table S1).

TABLE S1: Preservational Grade Criteria Summary (adapted from [1, 2])

Value	Description	Ichnotaphonomic Observations
3	All digit impressions sharp and clear; digit walls well defined; all ungual marks clearly preserved; distinct digital pads present. In quadrupedal animals; both manus and pes perfectly preserved.	Absence of mono- or multi- directional deformation of anatomically informative morphology, superimposition, and erosion.
2	Toe marks fairly clear and sharp (over substantial portions of the track); ungual marks and some digital pads recognizable.	Low occurrence of directional deformation, superimposition, and erosion.
1	Toe marks faint, blurred or distorted, but recognizable. Some ungual marks recognizable. For quadrupeds, manus prints distinguishable from pes prints. Only general outline preserved.	Considerable directional deformation. Extensive superimposition and erosion.
0	No visible morphological details.	Preponderance of directional deformation, superimposition or erosion.

Details of How Each Tridactyl Track Measurement Was Taken

With regard to tridactyl tracks, we measured L from the back of 'heel' to the most distal portion of digit III, and W between the outermost edges of lateral digits II and IV. We measured LII and LIV from the back of 'heel' to most distal portion of the respective digit and K and M from 'heel' to lowest point of the hypex. The basal digital lines were defined by connecting the terminations of K and M. We determined basal digital lengths (BL2, BL3, BL4) by measuring from the basal digital lines to distal end of each digit. We ascertained the basal digital widths (WBII, WBIII, WBIV) by measuring parallel the basal digital line of each digit and the middle digit widths (WMII, WMIII, WMIV) by measuring perpendicular to the middle of the basal digital lengths. We traced the interdigital angles between the central digit and the two lateral digits (α and β) between the overall digit length lines.

We assigned the tracks at the sites to different size classes based on the pes length (PL) (Table S2). These size classes then informed discussion of how many individual trackmakers might have traversed the sites overall.

Table S2: Dinosaur Footprint Size Classes

	Size Class	Pes Track Length (PL)
Sauropods	Tiny	PL < 25 cm
	Small	25 cm ≤ PL < 50 cm
	Medium	50 cm ≤ PL < 75 cm
	Large	PL ≥ 75 cm
Bipedal Dinosaurs	Tiny	PL < 10 cm
	Small	10 cm ≤ PL < 20 cm
	Medium	20 cm ≤ PL < 30 cm
	Large	PL ≥ 30 cm
Thyreophorans	Small	PW < 10 cm
	Medium	10 cm ≤ PW < 20 cm
	Large	20 cm ≤ PW < 35 cm
	Very Large	PW ≥ 35 cm

The size classes used to describe the dinosaur footprints at the site as defined by pes length (PL) or pes width (PW) (table redrafted from [3]).

Intervalometer Design Report

Problem Statement

The primary challenge encountered in flying a UAV (drone) along the coast of the Isle of Skye was that posted by unpredictable weather conditions. The UAV could not be safely or successfully flown with either extreme weather (rain and hail) or high wind conditions (> 15 mph). The weather on Skye is notoriously unpredictable and, on an hourly basis, can vary widely. When juxtaposing the weather conditions against the time windows during which the track-bearing platforms were exposed, the available windows for drone flight became vanishingly narrow. The situation imposed by the physical conditions of the site was further complicated by the necessity of having trained personnel available to fly the drone in the field.

Timing weather, tide, and human resources to fly the drone on Skye was attempted during fieldwork in November 2016 (unsuccessfully) and March 2017 (with partial success). The time- and resource-intensive nature of fieldwork on the Isle of Skye necessitated the successful collection of additional bedding-plane scale data sets during fieldwork in May 2017. However, the confluence of weather, tide, and human resource conditions for successful drone flight could not be guaranteed to coincide with the scheduled fieldwork.

The problem at hand can most colloquially be summarized with the question: "How can one collect a drone-based photogrammetry dataset without a drone?" More specifically, how can the aerial perspective, regular spacing of photographs (with predefined overlap), and efficiency of drone photography be duplicated without being reliant on ideal weather conditions?

Engineering Design

While an optimized design for the intervalometer was conceptualized in the lab, resource and time limitations resulted in the protoype differing from design specification in several key ways. In the following design summary, the optimized design will be reported and then contrasted with the actual design used for the prototype. Figures S1 and S2 shows the idealized design used to construct the intervalometer.

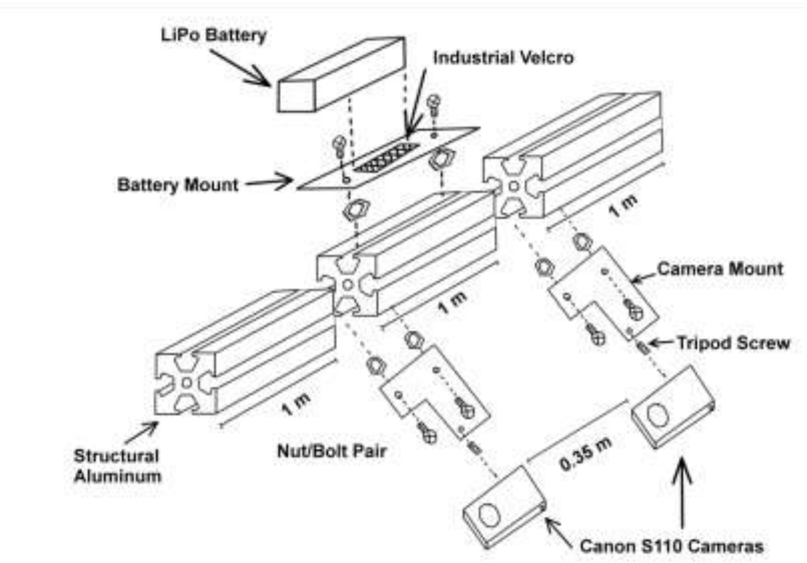

Figure S1: Intervalometer Design Schematic

The components necessary to build the intervalometer and the idealized distances of and between elements are shown. This sketch demonstrates the relative positioning of the elements of the intervalometer, but is not to scale.

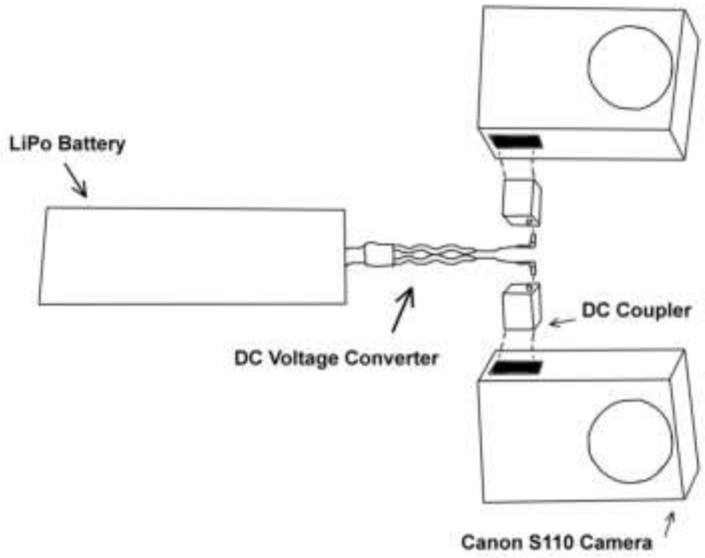

Figure S2: Details of LiPo Battery Connection

The connection between the LiPo battery and the cameras is shown. While on the intervalometer, the DC voltage converter hangs loose against the pole. It is recommended that loose wiring be secured with elastic or velcro straps to reduce the hazard of disconnecting the cameras from the power supply.

The pole of the intervalometer was composed of RS Pro aluminum alloy struts (40x40 mm, 8 mm grove) that were connected with screws with a 1 mm pitch to ensure that the grooves in adjoining struts could be aligned precisely. The optimized design for a machine held at approximately 1.5 m above the ground called for a pole 3 m long to remove the possibility of the operator's feet being in the image. The protoype was constructed of scrap aluminum struts from other projects and, therefore, was only 2.4 m long.

Two Canon S110 cameras (12 mp) were modified using CHDK (Canon Hacker Development Kit) firmware to enable a repeating timer (3s) to be set in order to trigger the shutter [4]. The script used to set the timer - interval.bas - was included in the basic CHDK package. Additional settings that were adjusted in the CHDK software were 'focus at infinity' (disabled) and 'autofocus' (enabled). These settings allowed for the camera to refocus with each sequential photograph and thus to correct to inconsistencies in the height at which the machine was held.

The cameras were then connected to a Bormatec 3s (3-cell) 8000 mAh battery (LiPo battery) in order that both could run off the same large power source instead of relying on the smaller battery provided by the camera manufacturer. A DC step-down voltage converter (a specialized cable used in UAV construction) was used to connect the battery with the cameras and to reduce the voltage produced by the battery to a level that was appropriate to the cameras. The voltage converter was connected to the cameras using a DC coupler (originally a component of a third-part AC adapter setup for the Canon S110 cameras).

The cameras were mounted on the pole using rough-made plywood supports with holes to accommodate the standard tripod thread size (Whitworth 1/4”-20). These plywood supports were then attached to the intervalometer pole using a two nut and bolt pairs that fit into the 8 mm groove in the structural aluminum pole. A similar plywood support was constructed for the LiPo battery and placed between the two camera supports. The LiPo battery was attached to the support with industrial velcro and secured with a two fabric straps.

Standard trigonometric equations relating the sensor size of the camera, the focal length of the lens, and the distance at which the object of interest was located were used to determine the necessary spacing of the cameras along the intervalometer pole. Canon S110 cameras have a 1/1.7” CMOS sensor (7.53 x 5.64

mm) with a total surface area of about 42.47 mm^2. The focal length of the lens ranges from 24 mm to 124 mm with a 35 mm equivalent.

In order to determine the area which a photograph covers, the sensor length and the focal length are related using the following equations (Eq. 1 and 2):

$$Width\ of\ Field\ of\ View = 2\ \times\ \tan^{-1}\frac{x\ sensor\ width}{2\ \times focal\ length} \tag{1}$$

$$Height\ of\ Field\ of\ View = 2\ \times\ \tan^{-1}\frac{y\ sensor\ width}{2\ \times focal\ length} \tag{2}$$

These equations result in the angles of view of the camera. These angles can then be coupled with the height at which the camera was held (set at 1.5 m in this case) to determine the distances captured (Eq. 3 and 4):

$$Width\ captured\ in\ Photo = Camera\ Height\ \times\ \tan(Width\ of\ Field\ of\ View) \tag{3}$$

$$Height\ captured\ in\ Photo = Camera\ Height\ \times \tan(Height\ of\ Field\ of\ View) \tag{4}$$

The necessary side lap between paired cameras for the construction of photogrammetric models is approximately 25-30%. The minimum amount of necessary overlap (25%) was chosen as a design parameter with an eye towards creating the widest possible spacing of cameras so that the outcrop could be captured with the least number of photographs. Similarly, the focal length was adjusted to 24 mm to allow for objects close to the camera to be viewed clearly. Table S3 summarizes the results of the camera calculations.

Table S3: Results of Camera Overlap Calculations

Width of Field of View (^{0})	17.8	Width captured in Photo (cm)	48.2
Height of Field of View (^{0})	13.4	Height captured in Photo (cm)	35.7
Desired Overlap between Cameras (cm)		12.1	

The results of the reported trigonometric equations as applied to Canon S110 specifications. The desired overlap between the cameras on the pole is based upon the width values because the cameras are placed with their long edge parallel to the pole ('right-side' up).

Using geometric constructions from similar triangles (Fig S3), the ideal distance between the cameras was calculated to be ~35 cm.

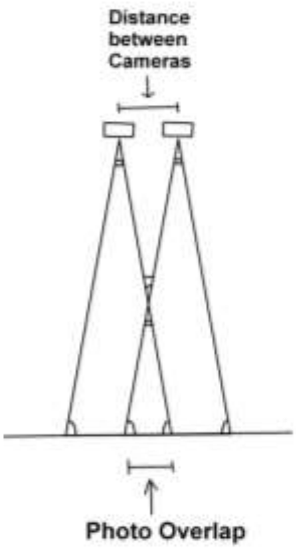

Figure S3: Similar Triangle Relationships in Camera Field of View

This schematic shows the similar triangles used to calculate the distance between cameras from the desired photo overlap.

The spacing of the cameras on the prototype was a little bit higher than the calculated ideal spacing at ~60 cm. This spacing resulted in a less than ideal amount of overlap, but was necessitated by the size of the LiPo battery used to power the set-up and the need to make sure the pole was balanced for easy use in the field. In order to account for the reduced sidelap between cameras, multiple sweeps of the platform at slightly different offsets were planned.

The distance for each successive point in the grid survey was calculated using a similar procedure for the height captured by the cameras and the assumption that an ideal photogrammetric survey requires ~60% end lap between sucessive photographs. However, the idealized distance (13.8 cm) was impracticable in terms of easy field measurements e.g. a pace. Therefore, the grid across the outcrop was set at 'one pace' intervals and additional sweeps were planned to make up for the lack of desired overlap.

Evaluation of Design Effectiveness

The intervalometer prototype was briefly tested under laboratory conditions to ascertain that the cameras would capture adequate overlap with each step forward along the imagined grid. Although both instrument operators' feet were observed in the test photographs, the desired overlap between photos was achieved. Due to the time constraints imposed by the short window between conception and prototyping, the prototype was used for data collection during fieldwork in May 2017 with the caveat of

ensuring instrument operators' stood along the outer edges of the pole instead of directly behind it. Figures S4 and S5 depict people operating the intervalometer in the field and are used with the consent of the research participants.The prototype worked sufficiently with regard to data collection, particularly at Brother's Point Site 3 (Fig S4).

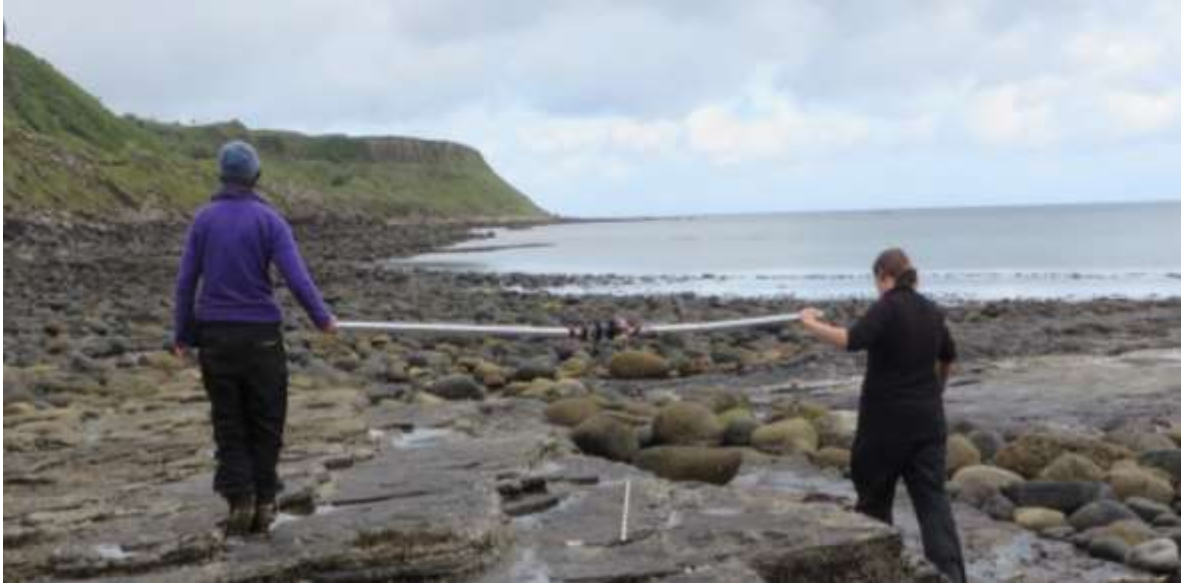

Figure S4: Intervalometer Data Collection

The intervalometer in use while surveying Brother's Point 3. Due to the stepping of the sedimentary layers, the operators needed to vary the height of the intervalometer relative to their own bodies in order to maintain a consistent height. Photo credit: Jon Hoad.

Once a rhythm was found in timing the steps taken with the clicks of the paired cameras, the outcrop was able to be covered with both efficiency and confidence. In the case of Brother's Point 3, multiple redundant sweeps were made, including some with the camera angled at about 25° above the horizontal. These redundancies proved critical in serving to fill holes in the model during post-processing.

The intervalometer was less successful at a different track locality (Brothers' Point Site 2 (BP2); site described in [5]) for several reasons linked to the nature of the site itself and the conditions under which the data were collected.

Since Agisoft Photoscan's tie-point detection algorithms work by identifying points within photos that remain invariant under different lighting conditions, it is vital for successful photo alignment and point cloud generation that the objects being modeled remain unchanged during the data collection stage. One of the instances where the detection algorithm struggles is with water features as ripples and other

surface disturbances are inherently transient and variable. Like BP1 and BP3, BP2 is located on an intertidal platform and is fully submerged for a portion of every day. The way in which the beds at BP2 step and the dominant, albeit slight, dip of the site mean that a significant amount of water pools against the raised edges of overlying layers and does not drain away during low tide (fig. 5, [5]). Since the intervalometer was held only 1.5 m above the ground surface, in contrast to the 3-6 m height of drone surveys, the pooled water tended to dominate the photoset. Additionally, the intervalometer operators had to step in the pools themselves in order to maintain the necessary spacing between subsequent photos generating additional ripples.

The topography of the site also proved challenging because tracks were located flush against small, but steep-sided rock features. Negotiating this topography resulted in the operators not keeping the cameras facing directly down (as assumed in the design calculations) (Fig S5).

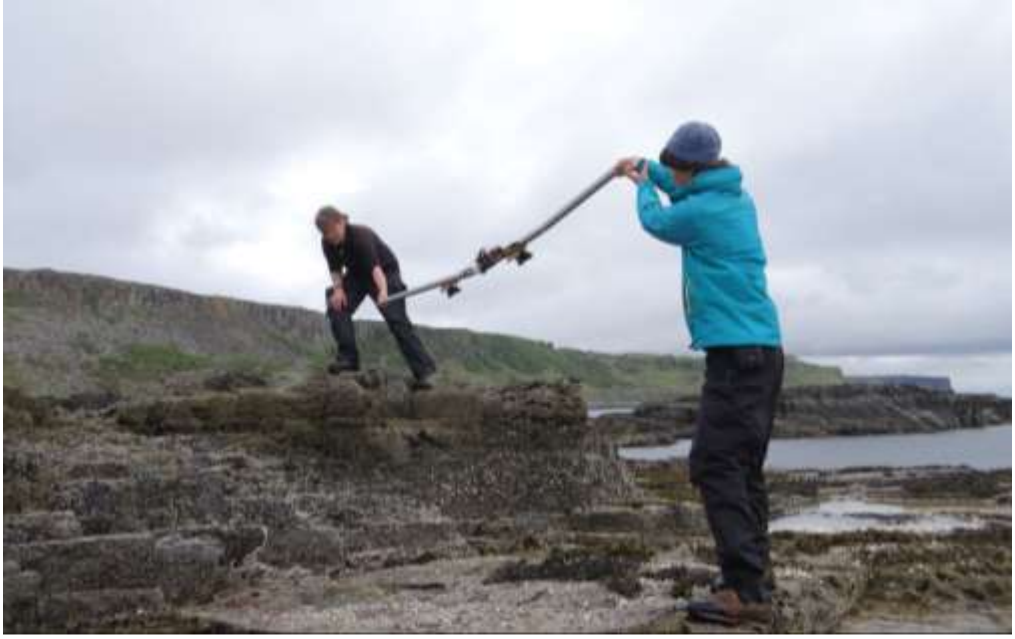

Figure S5: Challenging the Intervalometer Design

The topography of Brother's Point Site 2 also proved challenging in keeping the cameras angled directly downward (as was the assumption when design calculations were made). This resulted in a high number of photographs capturing the operator's feet and legs in addition to the outcrop. Photo credit: Shasta Marrero.

To compound these difficulties, a rain shower occurred at BP2 halfway through the completion of the survey. This event drastically changed the color and contrast between the rock surfaces and individual raindrops served to further disturb the surfaces of the pools. While the color change in the rocks was not

as significant a limiting factor in model construction as the pooled water, tie-points were not detected between the same areas before and after the rain shower. Thus, sizeable swaths of the dataset were excluded from automatic alignment because no matches could be found. The rain also meant that fewer redundant runs were collected due to concerns about the waterproofing of the cameras.

The prototype generated sub-satisfactory results with regard to several aspects.

- The feet of the instrument operators were often present along the edges of the photographs. This error derived from the pole being shorter than the distance calculated as necessary to avoid overlap between the images and the outermost ends of the pole.

- The screws which held the prototype pole together in the field because dirty and, were stripped, over the course of multiple constructions and deconstructions because the selected pitch of the screws was too narrow.

- The resulting photogrammetric dataset became unwieldy and difficult to use for larger scale outcrops (~ > 1000 square meters). The high photo density generated was a necessity from holding the pole at 1.5 m above the ground.

Several aspects of the prototype design could be improved for better performance in the field.

- The pole on which the cameras are mounted should be lengthened to eliminate the issue of feet being captured along the edges of the photographs.

- The threading of the screws used to construct the intervalometer pole should be selected at a coarser resolution (e.g. a 2 mm pitch instead of the 1 mm pitch used in the prototype) so that the pole is easier to construct in field conditions.

- The height at which the pole is held in the field can be increased by constructing additional extensors for the pole operators to hold. Increasing the height at which the pole is held will serve to decrease the number of photos necessary for complete coverage of the outcrop. This photo reduction will result in positive benefits in the processing stage of photogrammetric model construction.

- It is recommended that additional cameras be placed along the pole. The placement of additional cameras would increase the width of the outcrop that can be covered in a sweep and, thus, decrease the amount of time necessary to capture the whole outcrop (which is particularly desirable in situations where time is of the essence). Additionally, additional cameras would allow for a greater automation of the amount of overlap between the photos and reduce the amount of subjectiveness (lateral steps along the grid) in the survey by reducing the number of sweeps across the outcrop.

Despite the challenges in using the prototype in data collection, the intervalometer was successful in accomplishing its design objective – namely, capturing a photogrammetric dataset similar to that produced by flying a drone over an outcrop. The intervalometer demonstrates the greatest utility in capturing small to medium-sized outcrops (~180 to ~670 square meters) as the number of photos necessary to deal with a larger outcrop (4,387 photographs for ~3250 square meters) becomes unwieldy in photogrammetric software.

The simple construction of the intervalometer and low strain on existing resources makes the intervalometer a viable alternative for collecting photogrammetric datasets of small outcrops in conditions where drone flight is not possible or where the cost of flying a drone is prohibitive.

Standardized Agisoft Workflow for Tracksite Models

Preliminary quality control on the model data sets was initially conducted in the field. The photographs were briefly examined to check image quality (i.e. that they weren't blurry and that the color balance was acceptable) and image overlap (that ~60% overlap was achieved between successive images). When back on the processing computer, photographs that did not meet these general criteria were eliminated from the model data set.

Multiple passes were made over each site, which ensured that the initial data set had a large amount of redundancy in terms of photo coverage. This redundancy was useful because it allowed for flexibility with regard to which images were ultimately used in model construction.

The following workflow is detailed for Agisoft Photoscan 1.2.5.2735.

1) Import photographs into Agisoft Photoscan

    a. Using the tool 'Estimate Image Quality', the quality of each photograph is quantitatively assessed.

        - All photographs below a threshhold value of 0.5 were eliminated from the data set.

The remainder of the workflow used to construct the tracksite models can best be explicated using Agisoft Photoscan's "Workflow" menu with further details provided for each processing step.

2) Align photographs to build a sparse point cloud

    a. A lower quality ('Medium Accuracy') photo alignment was initially conducted on the whole dataset

        - The reconstructed camera positions were the checked to make sure that there were not any obvious inconsistencies in the camera's general location

        - The ground control markers were located in the photos and manually assigned at the pixel-level.

- The WGS 84 coordinates of each ground control marker were placed in a .csv file and imported into the “Reference” pane.

b. The camera alignment was optimized from the 'Reference' pane

c. The error values associated with the control points were assessed and ~25% of the control points were 'unchecked'. Unchecking the control points mean that they would not be used in constraining the model and could then serve as external check points to assess overall model quality. The combination of control and check points was determined through trial and error with the goal of reducing the total error (summed from latitudinal, longitudinal, and altitudinal error) on both sets of marker points.

d. A second, high quality ('High Accuracy') photo alignment was then run.

- Note: Depending on the number of photographs being aligned, this is one of the longest processing steps in the workflow.

e. The chunk in the 'Workspace' was then duplicated

- This allowed the processing work done so far to be saved in case later steps resulted in an undesirable output. Agisoft Photoscan has limited capabilities when it comes to 'undoing' steps.

f. Using the 'Gradual Selection' tool in the 'Edit' menu, a Reconstruction Uncertainty threshhold of 10 was set and all points above this threshhold were highlighted.

g. Using 'Delete Selection' in the 'Edit' menu, the selected points were removed from the model. This processing step removes tie-points (the calculated points of commonality between photos) with a high level of uncertainty.

h. Steps f and g were repeated to ensure that points above the threshhold value of 10 were eliminated.

i. The camera alignment was optimized from the 'Reference' pane and the project was saved.

3) Construct a dense point cloud

a. 'Build dense point cloud' was selected from the 'Workflow' menu

- Settings: High Density; Agressive Depth Filtering (enabled)

- This processing step is also extremely time-intensive.

4) Build polygon mesh.

a. 'Build mesh' was selected from the 'Workflow' menu

-Settings: Surface Type = Height field; Source data = Dense Cloud

b. Two meshes were constructed from the dense cloud – one with a 'high' face count as determined by Agisoft Photoscan and one with a 'medium' face count. The 'high' face count mesh was often quite resource intensive when it came to manipulating it on the screen, but made a more accurate basis for the orthophoto. The 'medium' face count mesh was easier to manipulate on the computer screen.

c. It is necessary to create a duplicate chunk of the model in between constructing the different meshes so that the data is not overwritten.

5) Construct orthophoto

a. 'Build orthomosaic' was selected from the 'Workflow' menu

-Settings: Projection = Geographic (WGS 84), Surface = Mesh, Blending Mode = Mosaic

- From the additional functions, hole filling was enabled and color correction was disabled.

Additional Processing Notes:

1. Approach to Masking Unnecessary Elements

Sometimes during the collection of the photogrammetric data sets in the field, small changes occurred in the surface being surveyed occurred during the survey or extraneous objects were photographed. For the most part, problematic photos could be excluded from the dataset entirely due to the redundancies built into the collection step.

However, sometimes, it was necessary to mask out objects that were pervasive throughout the images. In the case of this project, the most common element masked out of the images was the boots of those who were carrying the intervalometer as the pole was not quite long enough to ensure that the surveyers' feet were excluded from every image.

The masks were applied using the 'Intelligent Scissors' tool at the pixel level. At the photo alignment step, the option 'Constrain features by mask' was enabled. All other processing steps were executed as detailed in the generalized workflow.

c. Mesh Construction

When photogrammetric models of individual tracks were construct from field models or when the outcrop-scale point cloud was sub-sampled to create models of trackways/other features of interest, slightly different parameters were selected constructing the mesh.

-Settings: Surface Type = Arbitrary; Source Data = Dense Cloud; Face Count = High

Changing the surface type from 'height field' to 'arbitrary' allows for more details of the texture of the models to be emphasized at the cost of increased processing resources. 'Height field' is a mesh reconstruction algorithm that is optimized for primarily planar surfaces in that it bases the reconstruction projection direction along the z-axis of the model. It works particularly well for planar features and thus is appropriate for the bedding-planes of the full outcrop models. 'Arbitrary' is a mesh reconstruction algorithm that does not assume a dominant reconstruction projection direction. While the processing costs are prohibitive for using this mode on full outcrop scale, the reduced data set of features of interest/individual tracks makes it possible and, indeed, advantageous to use this mesh algorithm to draw out fine details of objects on a smaller scale.

This workflow was developed upon consultation with [6-8]

Reproducibility of Results

To allow for reproducibility of results and comply with best practices for three-dimensional data outlined in [9], the following materials are reposited at doi:10.5061/dryad.n6f068k in the Dryad data package associated with this manuscript:

(a) The photogrammetric models of each tracksite and subsampled models of areas of interest [file type: .ply];

(b) The point clouds of the models [file type: .ply];

(c) The original images used to construct the model [file type: .jpg];

(d) The ground control points used to constrain the models [file type: .csv].

The repository is organized with the following contents:

A. README.txt
B. BP1_Model_Dataset.zip
C. BP3_Model_Dataset.zip
D. Full_Tracksite_Models.zip (and Full_Tracksite_Models_Mac.zip)
E. High_Quality_Orthophotos.zip
F. Subsampled_Areas_of_Interest.zip

When downloading data from the repository, a link to a .tar.gz file will be sent to the recipient's email address. The procedure for opening tar files varies between operating systems and are roughly outlined below:

- For Linus and Mac OS, the following command line can be input into the terminal to extract files from a .tar.gz (where *filename* denotes the file to be opened).
    - tar –xzf *filename*
- For Windows OS, an external program (like the opensource software 7-Zip) is necessary.
    - Download and install 7-Zip (or alternative program of choice)
    - Right-click .tar.gz file, select '7-Zip' from resultant menu, and select 'Extract files"
    - Select a new file location for the extracted files and press 'Okay'

*Notes Regarding File Usability:

- The full tracksite model meshes (.ply files) can cause lag when manipulated using rotation and zoom functions in standard 3D viewing software.

- On older OSs, the High Quality Orthophoto .jpgs may take several minutes to open.

- The Full_Tracksite_Models.zip folder does not open reliably on the MacOS. Files can be extracted from the alternative folder Full_Tracksite Models_Mac.zip.

Assessment of Photogrammetric Models

With the increasing prevalence of the use of photogrammetric models in documenting tracksites, an increasing need for rigorous evaluation of the quality of the models exists. Agisoft Photoscan has several useful internal metrics for model evaluation which were reported for each of the generated models. Additionally, each model's correspondence to the reality of the tracksite was evaluated using a Taylor diagram. Taylor diagrams were originally developed as a way of graphically assessing pattern correlations using basic summary statistics and are often applied to the evaluation of climate models [10]. Since Taylor diagrams compare modeled results with reference data, they can also be used to evaluate how well models correspond to reality. In this study, Taylor diagrams were generated by comparing track measurements taken in the field with the same measurements taken off the point clouds of the general models. Although [10] suggested that this graphical representation was not strictly necessary in comparing the differences between a single model and a frame of reference as a table of summary statistics could provide the same information, these single model diagrams were found useful in comparing the quality of the individual site models and in parsing through slight site-specific differences (Fig S6).

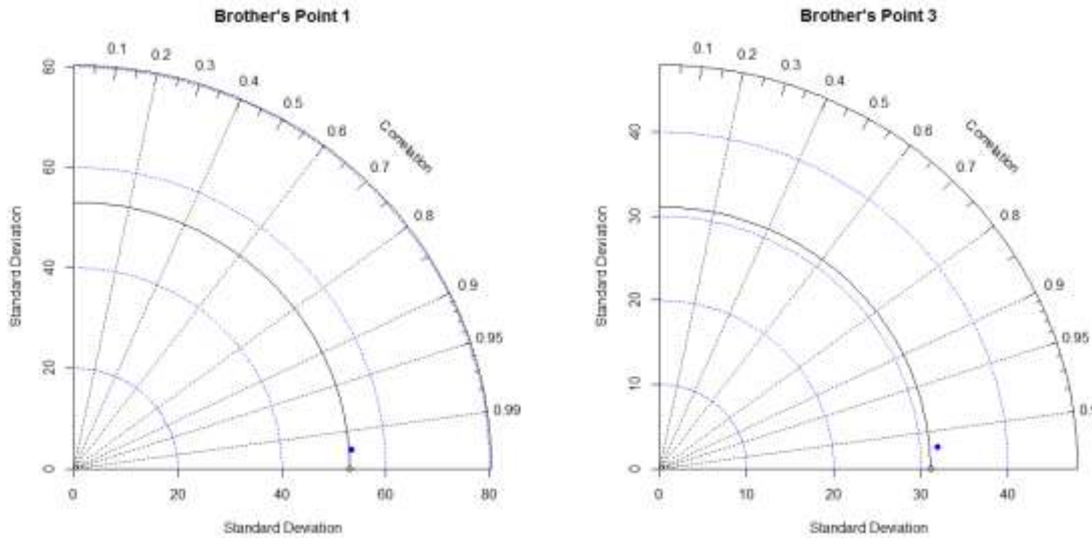

Figure S6: Taylor Diagrams of BP1 and BP3

The Taylor diagrams evaluating both sites show a high correspondence between the reference measurements taken in the field (shown by an open circle on the x-axis) and the same measurements made in the model (shown by the blue circle).

Overall, the >0.99 correlation coefficients between the field data and the model data mean that measurements taken from the models can be considered representative of the reality of the track bearing surface. The standard deviation of the BP1 model measurements was slightly closer to the reference standard deviation than that of BP3. This difference may partially be explained by the high contrast between the limestone casts infilling many of the footprints and the dark shale in which the tracks were impressed making locating the precise edge of the tracks easier in the model of BP1.

Tables S4 and S5 show the measurement comparisons that underlie the construction of the Taylor diagrams. It is noted that some of the measurements reported here are not congruent with other length measurements listed for the same footprints elsewhere in the text. These incongruencies arise from electing to choose model validation measurement lines between clear features on the track so that the model quality can be more easily assessed.

Table S4: BP1 Measurement Comparison for Taylor Diagram

Track ID	Measurement	Field (cm)	Model (cm)
BP1_1	Length (L)	29	23.7
	Width (W)	24	24.4
BP1_3	Long Axis (LA)	23.5	21.8
	Short Axis (SA)	17	13.2
BP1_4	Long Axis (LA)	20	19.3
	Short Axis (SA)	20	19.9
BP1_5	Long Axis (LA)	22	21
	Short Axis (SA)	15	16.6
BP1_6	Length (L)	16.4	16.3
	Width (W)	14	14.4
BP1_7	Long Axis (LA)	15	15.7
BP1_8	Long Axis (LA)	16.5	16.3
BP1_9	Long Axis (LA)	15	14.6
BP1_11	Long Axis (LA)	16	14.4
BP1_14	Length (L)	19	18.8
	Width (W)	18.5	17.3
BP1_16	Length (L)	38	30.6
	Width (W)	20	21.3
BP1_17	Length (L)	28	27.1
	Width (W)	18	16.3
BP1_26	Length (L)	35.5	36
	Width (W)	27	26.6
BP1_30	Length (L)	58	37
	Width (W)	34	35.4
BP1_32	Length (L)	41	39.5
	Width (W)	32	31.3
BP1_34	Length (L)	16	13.2
	Width (W)	13	12.4
D_7_8		30	30
D_8_9		20.5	20
D_7_9		50	51.2
D_10_11		21	21.3
D_16_17		87	86.9
D_20_21		69	70.6
D_21_22		58	57.7
D_20_22		109	114.4
D_22_23		147	144
D_26_27		122	122
D_27_28		115	112.5
D_26_28		227	232.9
D_28_29		123	117.4
D_27_29		228	226.4
D_30_32		132	131.6
D_31_32		87	89.1
D_30_31		96	96.7

Table S5: BP3 Measurement Comparison for Taylor Diagram

Track ID	Measurement	Field (cm)	Model (cm)
BP3-1	Length (L)	33	32.7
	Width (W)	32	31.9
BP3-2	Length (L)	23.5	22.4
	Width (W)	27	25.9
BP3-3	Length (L)	38	37
	Width (W)	30.4	29.6
BP3-4	Length (L)	19.2	21.6
	Width (W)	21	22.7
BP3-5	Length (L)	23.7	23.6
	Width (W)	24.3	26.5
BP3-6	Length (L)	30.3	30.6
	Width (W)	18	16.5
BP3-7	Length (L)	20.4	19.9
	Width (W)	15.5	15.7
BP3-8	Length (L)	38.3	38.3
	Width (W)	35.4	34.2
BP3-9	Length (L)	30.5	30.6
	Width (W)	31.1	30.5
BP3-10	Length (L)	37.2	42.2
	Width (W)	31.2	31.4
BP3-11	Length (L)	32.2	32.7
	Width (W)	28.4	28.2
BP3-12	Length (L)	36.4	34.2
	Width (W)	33.2	32.7
BP3-13	Length (L)	8.9	8.1
	Width (W)	6.1	5.9
BP3-15	Length (L)	47.5	48.7
	Width (W)	33.2	36
32	Length (L)	50	46.8
	Width (W)	42	44.6
D_2_3		82	80
D_3_4		112	108.1
D_3_5		105.5	106.4
D_5_6		110	121.4
D_4_10		69	70.2
D_10_15		80.4	79
D_10_16		147	153
D_15_16		67.4	65.3

Additional False Color Depth Images from Photogrammetric Models

While false color depth images were included in the main text for the majority of the tracks discussed, some of the resultant images were blurry or otherwise uninformative due to the scale of the features or the resolution of the model. These images are of the exemplar *Deltapodus* tracks at BP1 (Fig S7) and of the trackways at BP3 (with BP3_Twy_01 in Fig S8 and BP3_Twy_02 in Fig S9).

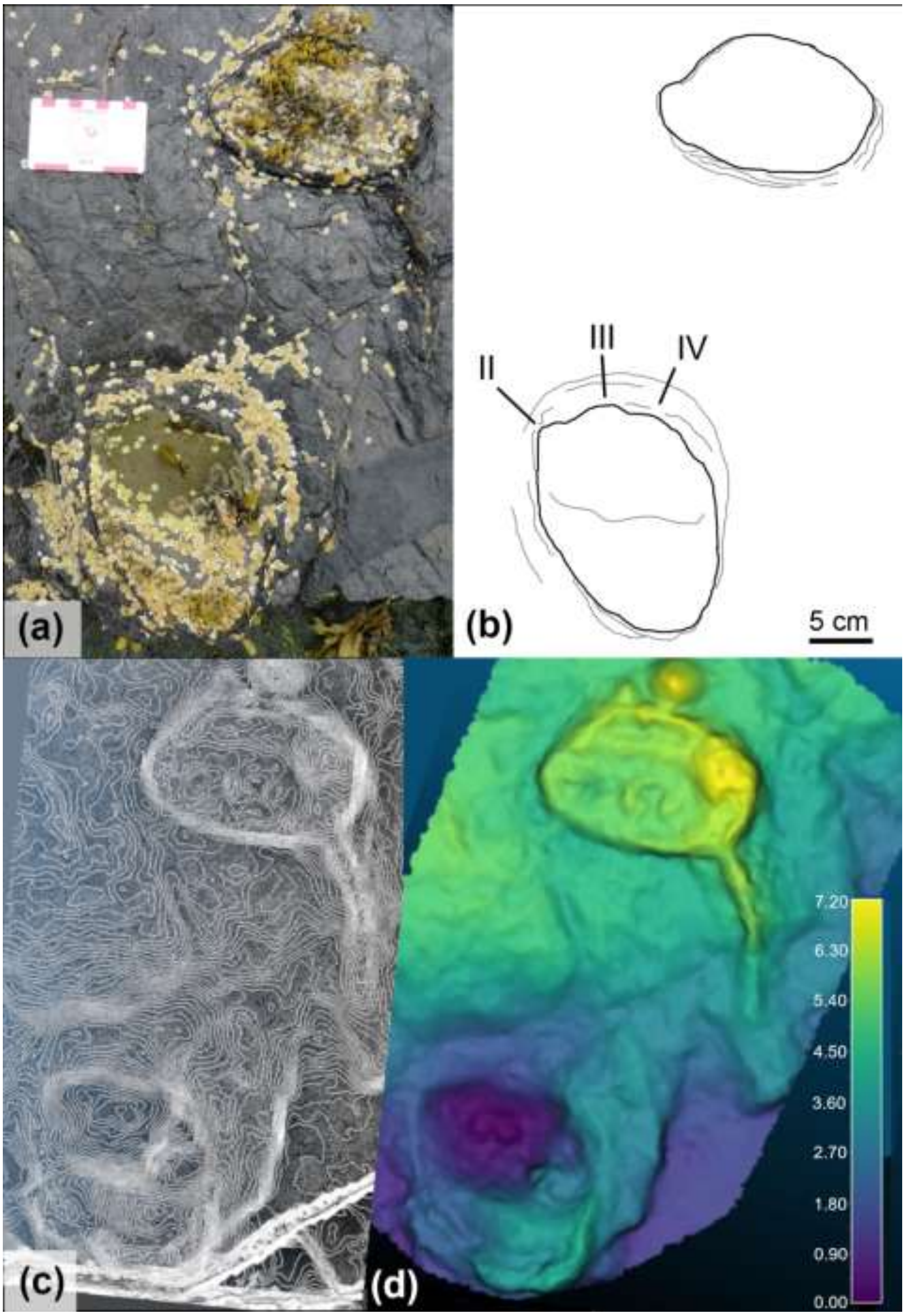

Figure S7: Photogrammetric Renderings of BP3_Twy_01 exemplar tracks

The (a) field photo, (b) line drawing, and (c) contour map of the main text have be placed with a false depth color map of the exemplar tracks of BP1_Twy_01. The color scale of (d) is in units of cm.

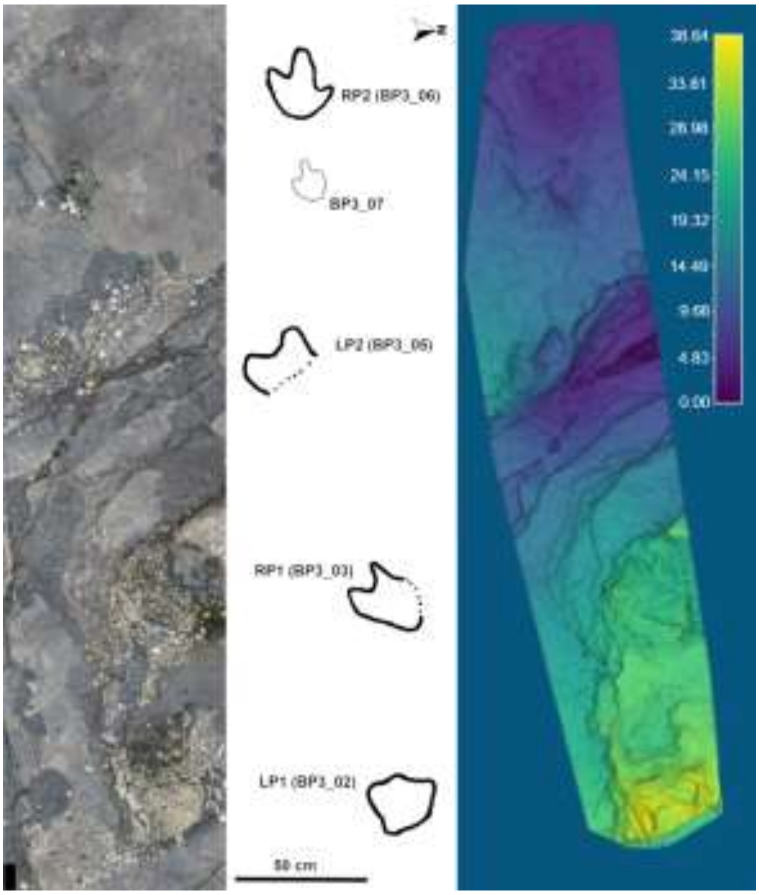

Figure S8: Photogrammetric Renderings of BP3_Twy_01

The field photo and line drawing of the main text have be placed with a false depth color map of the BP3_Twy_01. The color scale of the photogrammetric rendering is in units of cm.

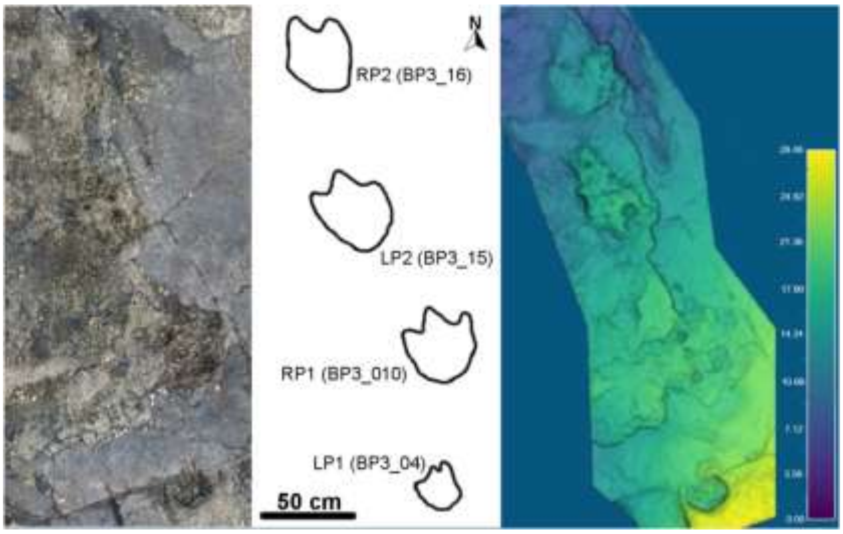

Figure S7: Photogrammetric Renderings of BP3_Twy_02

The field photo and line drawing of the main text have be placed with a false depth color map of the BP3_Twy_02. The color scale of the photogrammetric rendering is in units of cm.

Comprehensive Tracksite Measurements

The following set of tables (S6, S7, S8, S9, S10, S11, S12, S13) compiles all measurements and some qualitative observations made on the tracks from BP1 and BP3. Table S14 illustrates the preservation grades assigned to each of the footprints at the sites.

Table S6: BP1 Tridactyl Track Measurements

	L	W	K	M	BL2	BL3	BL4	LII	LIII	LIV	WBII	WBIII	WBIV	WMII	WMIII	WMIV	α	β
BP1_01	32.5	24.7	19.8	19.5	7.8	12.3	n/a	25.3	32.5	n/a	7.2	7.6	n/a	4.6	3	n/a	30.7	n/a
BP1_02	24.1	23.6	14.2	n/a	5.2	10.2	n/a	17	24.1	n/a	7.7	9.8	n/a	3.6	5.6	n/a	34.5	n/a
BP1_06	16.4	13.7	7.3	8.7	3.7	6.6	4	10.2	16.4	10.9	3	5	2.1	2.5	3.1	2.5	32.2	29.3
BP1_16	30.5	21	18.9	17.9	7.8	11.4	n/a	25.5	30.5	n/a	3.4	3.8	n/a	1.8	1.6	n/a	20.5	n/a
BP1_17	27.3	20.8	n/a	12.7	n/a	13.4	8.4	n/a	27.3	18.1	n/a	2.5	3.8	n/a	2.2	1.9	n/a	35.3
BP1_30	37.6	>31.4	27.4	21.3	>8.4	16.5	16.5	>30.1	37.6	35.5	5.56	8.6	7.8	3.2	3.8	5.6	20.5	34.9
BP1_32	40	>35.2	22.4	20.7	12.9	16.6	n/a	34.2	40	n/a	8.3	9.1	n/a	5.5	7.3	n/a	26.4	n/a
BP1_34	17.9	14.1	10.1	9.2	4.4	7.6	3.6	12.9	17.9	11.1	5	4	3	2.5	3.3	3.3	25.9	32.1

All measurements taken on the distinctive tridactyl tracks at Brother's Point Site 1 are summarized in this table (ref. manuscript Fig 2 for measurement abbreviations). All values are shown in centimeters with the exception of α and β (both in degrees).

Table S7: Parameters for Evaluating Theropod/Ornithopod Affinity at BP1

Track Parameters	Threshold values and probability that the track is either theropod or ornithopod	BP1_01	BP1_02	BP1_06	BP1_16	BP1-17	BP1-30	BP1-32	BP1-34
L/W	80.0% Theropod > 1.25 > Ornithopod 88.2 %	1.32	1.02	1.20	1.45	1.31	1.20	1.14	1.27
L/K	70.5 % Theropod > 2.00 > Ornithopod 88.0%	1.64	1.70	2.25	1.61		1.37	1.79	1.77
L/M	65.0% Theropod > 2.00 > Ornithopod 90.7%	1.67		1.89	1.70	2.15	1.765	1.93	1.95
BL2/WMII	76.1% Theropod > 2.00 > Ornithopod 97.4%	1.70	1.44	1.48	4.33		2.63	2.35	1.76
BL3/WMIII	72.7% Theropod > 2.20 > Ornithopod 97.7%	4.10	1.82	2.13	7.13	6.09	4.34	2.27	2.30
BL4/WMIV	76.1% Theropod > 2.00 > Ornithopod 97.6%			1.60		4.42	2.95		1.09
LII/WBII	84.6% Theropod > 3.75 > Ornithopod 90.2%	3.51	2.21	3.40	7.50		5.41	4.12	2.58
LIII/WBIII	70.6 % Theropod > 4.00 > Ornithopod 91.5 %	4.28	2.46	3.28	8.03	10.92	4.37	4.40	4.48
LIV/WBIV	73.7% Theropod > 3.75 > Ornithopod 93.4 %			5.19		4.76	4.55		3.70

The parameters for evaluating theropod/ornithopod affinity are summarized for the measurable tridactyl tracks of BP1. The tridactyl tracks at BP1 show a mixture of theropod and ornithopod affinities and indicate a variety of trackmakers were present at the site. Blank spaces in the table represent values that could not be calculated do to track incompleteness.

Table S8: BP1 Bipedal Trackway Measurements

	RP[1]	RP[2]	LP	S[1]	S[2]	WAP[1]	WAP[2]	γ[1]	γ[2]	α	Total Length	Orientation
BP1_Twy_02	0.70	n/a	0.88	1.45	n/a	0.50	n/a	131	n/a	-10.4	1.73	155
BP1_Twy_03	1.28	1.04	1.24	2.34	2.28	0.39	0.50	150	156	n/a	3.80	100

The measurements made on the two bipedal trackways present at Brother's Point Site 1 are summarized here. All measurements are reported in meters except for γ, α, and trackway orientation which are measured in degrees. RP[1] and RP[2] are the left pace measurements taken between RP1 and LP1 and RP2 and LP2 respectively. For BP1_Twy_01, RP[1] is the only right pace measurement taken. The track designations are figured on the outline drawings provided in the main text. LP is the left pace measurements. S[1] and S[2] are the stride lengths between the right feet and the left feet respectively in BP1_Twy_02. S[1] denotes the only measurable stride length in BP1_Twy_01 and BP1_Twy_03. For BP1_Twy_02, WAP[1] is the trackway width measured between RP1 and RP2 with LP1 as the focal point while WAP[2] is the trackway width measured between LP1 and LP2 with RP2 as the focal point. WAP[1] for BP1_Twy_02 and BP1_Twy_04 denotes the trackway width measured between stride line and the opposite footprint. WAP[1] denotes the trackway width of BP1_Twy_02 (RP1) and BP1_Twy_04 (LP1). The track designations in parentheses served as the focal points for the trackway width measurements. γ is the pace angulation of the trackway. For BP1_Twy_03, γ is the pace angulation with [1] using RP1 as the vertex of the angle and [2] using LP2 as the vertex of the angle. α measures the track rotation of individual footprints (LP1 in BP1_Twy_02 and RP1 in BP1_Twy_04). The rotation for BP1_Twy_02 is towards the midline (inward rotation = negative angle) while the rotation for BP1_Twy_04 is away from the midline. This value has a relatively low confidence level since, in all cases the trackways were short and the relationship of individual tracks to the midline was difficult to determine.

Table S9: Quadrupedal Trackway Measurements

	RPP	LPP	RMP	RPP(?)	PS	PS(?)	WAP	WAP (?)	Pace Angulation	Trackway Length	Trackway Orientation	IMS	IPS	Heteropody [R]	Heteropody [L]
BP1_Twy_01	59	62	61	48	52	58	52	46	52	60	105	14.8	14.4	1.7	1.5

The trackway measurements for the BP1_Twy_01 at BP1. RPP and LPP are the right pes pace and the left pes pace respectively. RMP is the right manus pace. RPP(?) denotes the right pes pace if the closer of the two weakly associated tracks is assumed to be part of the trackway. WAP is the width of the angulation pattern of the trackway and WAP(?) denotes this value as measured assuming that the close of the two weakly associated tracks is part of the trackway. The index of manus size (IMS) was calculated for both manus impressions in the trackway (14.8 and 15.4) and then averaged. A similar approach was used to calculate the index of pes size (IPS) with the IPS for being calculated for RP1, RP2, and LP1 and then averaged. The heteropody values were tabulated using the ratio pes/manus with [R] denoting the right manus/pes set and [L] denoting the left manus/pes set. All values are in centimeters except for pace angulation and trackway orientation (both in degrees) and IMS, IPS, and heteropody (dimensionless ratios). IMS, IPS, and heteropody calculated according to methods outlined in [11].

Table S10: Measurements of Ovoid and Indistinct Tracks at BP1

	Long Axis (LA)	Short Axis (SA)	Brief Description of Track Irregularity
BP1_3	23.8	14.7	Suboval cast, *Deltapodus* track morphology
BP1_4	17.7	14.1	Suboval impression with no distinctive toes or heel
BP1_5	20.6	16.9	Suboval cast with sediment deformation around edges
BP1_7	18.9	13.6	First right pes impression in Trackway 1, suboval cast
BP1_8	19.4	12.3	First right manus impression in Trackway 1, suboval cast
BP1_9	14.4	10.7	Second right pes impression in Trackway 1, suboval cast
BP1_10	15.9	13.7	Left pes impression in Trackway 1, suboval cast cut by crack
BP1_11	17.2	11.7	Left manus impression in Trackway 1, suboval cast
BP1_12	14.5	14.1	Suboval cast with sediment deformation rim
BP1_13	26.7	15.1	Suboval cast, partially covered by overlying limestone
BP1_14	21.2	18.5	Isolated suboval impression, *Deltapodus* track morphology
BP1_15	20.1	9.2	Leftmost digit of track possibly associated with Trackway 2; rest of the track obscured by overlying layer
BP1_18	15.5	15.0	Shallow impression in shale layer is indistinct boundaries
BP1_19	19.8	14.8	Very thin cast cross-cut by mudcracks
BP1_20	16.1	15.0	Track cast visible through crack in overlying limestone
BP1_21	16.1	15.8	Broken cast surrounded by large sediment deformation rim
BP1_22	16.8	11.3	Broken cast surrounded by large sediment deformation rim
BP1_23	12.3	10.4	Broken cast surrounded by large sediment deformation rim
BP1_24	25.1	16.6	Large oval cast with no distinctive features
BP1_25	25.1	16.2	Possible composite track (two casts overlying one another)
BP1_26	32.7	25.1	Cast of likely tridactyl track but both lateral digits are broken
BP1_27	39.6	30.8	Impression of first left pes of Trackway 3
BP1_28	31.0	21.0	Shallow cast and impression of second right pes of Tway 3
BP1_29	19.7	19.5	Shallow impression; broken along the edge of the platform
BP1_31	36.9	33	Impression of the left pes of Trackway 4
BP1_33	8.6	7.3	Broken cast near the edge of the platform
BP1_35	9.1	8.0	Broken cast of tridactyl print preserving only digit III
BP1_P1	16.1	13.3	Shallow sub-circular impression without clear boundaries
BP1_P2	14.8	12.9	Heavily weathered cast cross cut by mudcracks
BP1_PG_a	16.9	16.6	Shallow sub-circular impression without clear boundaries
BP1_PG_b	22.0	20.0	Shallow sub-circular impression without clear boundaries
BP1_PB_c	20.3	17.3	Shallow sub-circular impression without clear boundaries

Long and short axis measurements of all ovoid tracks and tracks with indistinct characteristics are tabulated with a brief description of the footprints. All measurements are in centimeters.

Table S11: BP3 Tridactyl Track Measurements

	L	W	K	M	BL2	BL3	BL4	LII	LIII	LIV	WBII	WBIII	WBIV	WMII	WMIII	WMIV	α	β
BP3_01	33.1	33.7	21.1	24.4	8.1	9.6	5.0	27.4	33.1	26.2	7.4	10.1	6.7	4.4	5.9	5.9	44.3	31.9
BP3_03	33.0	32.7	29.8	26.7	5.2	7.8	n/a	31.0	33.0	n/a	8.1	10.0	n/a	5.2	5.3	n/a	26.2	n/a
BP3_06	30.8	33.9	21.3	22.1	8.5	8.9	10.4	25.4	30.8	27.1	6.4	8.2	6.7	4.9	5.5	6.2	41.3	44.7
BP3_07	20.6	15.6	14.6	15.4	2.9	6.9	2.5	15.6	20.6	16.1	4.1	5.7	3	3	3.5	1.8	27.6	24.3
BP3_08	40.2	35.0	36.4	31.1	7.5	10.5	6.2	32.3	40.2	33.2	11.2	10.6	9.9	7.2	7.4	5.7	38.5	23.8
BP3_09	34.6	31.0	25.7	25.1	7.0	8.1	n/a	29.8	34.6	n/a	7.1	11.2	n/a	4.1	6.3	n/a	40.1	n/a
BP3_10	39.5	29.3	24.2	23.8	10.5	13.6	11.4	32.8	39.5	34.8	8.2	7.1	6.6	3.2	3.7	5.0	23.7	29.6
BP3_11	28.8	28.3	20.3	18.2	7.8	11.2	9.0	27.4	28.8	22.7	7.6	9.0	8.3	4.5	5.2	5.3	45.0	27.0
BP3_12	36.6	28.5	26.6	28.4	n/a	8.8	8.0	n/a	36.6	31.1	n/a	7.7	11.7	n/a	4.1	8.7	26.1	27.2
BP3_13	8.9	5.1	3.4	4.6	2.8	4.3	2.1	5.3	8.9	5.0	1.7	1.3	1.1	0.9	1.0	0.9	27.0	21.0
BP3_14	12.9	14.6	8.0	7.7	7.8	7.3	n/a	12.2	12.9	n/a	3.9	6.5	n/a	1.8	2.8	n/a	26.3	32.1
BP3_15	44.8	35.8	n/a	30.4	n/a	14.1	11.5	n/a	44.8	39.7	n/a	9.0	8.2	n/a	4.8	4.9	n/a	28.9
BP3_17	24.8	19.3	14.6	n/a	7.6	11.7	n/a	21.9	24.8	n/a	6.7	10.6	n/a	5.0	7.0	n/a	39.3	n/a
BP3_18	30.0	30.0	22.6	20.9	6.2	11.3	10.9	24.8	30.0	27.1	6.9	11.8	9.3	6.0	7.6	7.3	34.3	29.3

All measurements taken on the distinctive tridactyl tracks at Brother's Point Site 3 are summarized in this table (ref. Fig. 2 for measurement abbreviations). All values are shown in centimeters with the exception of α and β (both in degrees).

Table S12: Measurements of Indistinct Tracks at BP3

	Long Axis (LA)	Short Axis (SA)	Brief Description of Track Irregularity
BP3_02	30.3	26.5	Track belongs to BP3_Twy_1, but is along brokenedge of platform with indistinct toes
BP3_04	24.1	18.1	Track belongs to BP3_Twy2, but is limited to deep 'heel' impression with weak digital remains
BP3-05	24.8	21.4	Track belongs to BP3_Twy1, but majority is along broken edge of platform
BP3_16	50.4	51.6	Track belongs to BP3_Twy2, but is almost completely obscured by overlying limestone

Basic measurements of tracks with indistinctive features are presented with brief explanations of why more detail measurements were not made in these cases. Long axis measurements are taken along the longest possible portion of the track and short axis measurements are made perpendicular to the long axis. In the case of these tracks, the long axis was usually roughly parallel to the line drawn between the inferred locations of digit III and the heel. All measurements are reported in centimeters.

Table S13: Parameters for Evaluating Theropod/Ornithopod Affinity at BP3

Track Parameters	Threshold values and probability that the track is either theropod or ornithopod	BP3_01	BP3_03	BP3_06	BP3_07	BP3_08	BP3_09	BP3_10	BP3_11	BP3_14
L/W	80.0% Theropod > 1.25 > Ornithopod 88.2 %	.98	1.01	0.91	1.32	1.15	1.12	1.34	1.02	0.88
L/K	70.5 % Theropod > 2.00 > Ornithopod 88.0%	1.57	1.14	1.45	1.41	1.10	1.35	1.63	1.42	1.61
L/M	65.0% Theropod > 2.00 > Ornithopod 90.7%	1.36	1.24	1.39	1.34	1.29	1.38	1.66	1.58	1.68
BL2/WMII	76.1% Theropod > 2.00 > Ornithopod 97.4%	1.84	1.00	1.73	0.97	1.04	1.71	3.28	1.73	4.44
BL3/WMIII	72.7% Theropod > 2.20 > Ornithopod 97.7%	1.63	1.47	1.62	1.97	1.42	1.29	3.68	2.15	2.61
BL4/WMIV	76.1% Theropod > 2.00 > Ornithopod 97.6%	0.85		1.68	1.39	1.09		2.28	1.70	n/a
LII/WBII	84.6% Theropod > 3.75 > Ornithopod 90.2%	3.70	3.83	3.97	3.80	2.88	4.20	4	3.61	3.13
LIII/WBIII	70.6 % Theropod > 4.00 > Ornithopod 91.5 %	3.28	3.30	3.76	3.61	3.79	3.09	5.56	3.20	1.98
LIV/WBIV	73.7% Theropod > 3.75 > Ornithopod 93.4 %	3.91		4.04	0.60	3.35		5.27	2.73	n/a

The parameters for evaluating theropod/ornithopod affinity are summarized for the measurable tridactyl tracks of BP3. Notably, the components of Trackway 1 and the tracks concentrated in the souther portion of the platform demonstrate a dominantly ornithopod affinity. Blank cells indicate ratios that could not be calculated due to one of the component measurements not being attainable.

Table S14: Preservation Grades of Footprints from BP1 and BP3 with Reasoning for Each Assignment

Track Identification	Preservation Grade		Track Identification	Preservation Grade
BP1_01	1		BP3_01	1
BP1_02	0		BP3_02	1
BP1_03	1		BP3_03	0
BP1_04	0		BP3_04	0
BP1_05	0		BP3_05	1
BP1_06	1		BP3_06	1
BP1_07	1		BP3_07	1
BP1_08	1		BP3_08	1
BP1_09	1		BP3_09	0
BP1_10	1		BP3_10	0
BP1_11	1		BP3_11	1
BP1_12	0		BP3_12	1
BP1_13	0		BP3_13	1
BP1_14	1		BP3_14	0
BP1_15	0		BP3_15	0
BP1_16	1		BP3_16	0
BP1_17	1		BP3_17	1
BP1_18	0		BP3_18	0
BP1_19	0			
BP1_20	0			
BP1_21	0			
BP1_22	0			
BP1_23	0			
BP1_24	0			
BP1_25	0			
BP1_26	1			
BP1_27	0			
BP1_28	0			
BP1_29	0			
BP1_30	1			
BP1_31	0			
BP1_32	1			
BP1_33	0			
BP1_34	1			
BP1_35	0			
P1	0			
P2	0			
PG	0			

Details of *Deltapodus* Track Length Calculations

Four distinctive *Deltapodus* pes footprints are present at BP1 (BP1_03, BP1_07, BP1_09, BP1_10). The measured lengths of these footprints (23.8 cm, 18.9 cm, 14.4 cm, and 15.9 cm, respectively) were used in determining the average and standard deviation reported in the main text.

Determining the average *Deltapodus* pes length for the Yorkshire specimens proved more challenging as the data are presented as a scatterplot in [12], but not formally tabulated. Table 11 summarizes the estimated pes lengths from the scatterplot (cf. Fig. [12]).

Table S15: Estimated Pes Lengths of Yorkshire *Deltapodus* tracks (cm)

8.3	11.1	18.8	21	24.5	26.6	27.5	27.5	28	29
30	30	30.5	30.5	30.5	32.7	33	34	34	35
36.4	37	37.8	37.8	37.8	37.8	38	38	38.5	38.5
39	39	40	40.5	42.2	42.2	42.2	42.2	42.2	43
44	45	45	46.1	47.8	47.8				

Using these estimated values, the average pes length for the Yorkshire tracks was ~35 cm with a standard deviation of ~8.8 cm.

The average pes length reported for BP1_Twy_01 was determined based on the average of BP1_07 and BP1_10 (the most unequivocal pes impressions). This average length was used in subsequent hip height calcualtions.

References

1. Belvedere M, Farlow JO. A numerical scale for quantifying the quality of preservation of vertebrate tracks. In: Falkingham PL, Marty D, Richter A, editors. Dinosaur Tracks: The Next Steps. Bloomington: Indiana University Press; 2016. p. 92-98.
2. Marchetti L, Belvedere M, Voigt S, Klein H, Castanera D, Diaz-Martinez I, Marty D, Xing L, Feola S, Melchor RN, Farlow JO. Defining the morphological quality of fossil footprints. Problems and principles of preservation in tetrapod ichnology with examples from the Palaeozoic to the present. Ear. Sci. Rev. 2019; 193: 109-145. doi: 10.1016/j.earscirev.2019.04.008.
3. Salisbury SW, Romilio A. Herne MC, Tucker RT, Nair JP. The dinosaurian ichnofauna of the Lower Cretaceous (Valanginian-Barremian) Broome Sandstone of the Walmadany Area (James Price Point), Dampier Peninsula, Western Australia. J Vertebr Paleontol. 2016;36 1-152. doi: 10.1080/02724634.2016.1369539.
4. CHDK Development Team (2017). Canon Hack Development Kit. URL http://chdk.wikia.com/wiki/CHDK.
5. dePolo PE, Brusatte SL, Challands TJ, Foffa D, Ross DA, Wilkinson M, Yi H-Y. A sauropod-dominated tracksite from Rubha nam Brathairean (Brothers' Point), Isle of Skye, Scotland. Scott J Geol. 2018;54: 1-12. doi: 10.1144/sjg2017-016.
6. Mallison H, Wings O. Photogrammetry in Paleontology – A Practical Guide. Journal of Paleontological Techniques. 2014;12: 1-31.
7. Mallison, H. Photogrammetry Tutorial 11: How to handle a project in Agisoft Photoscan. 2015 Oct. 11 [cited 2019 March 30]. Available from: https://dinosaurpalaeo.wordpress.com/2015/10/11/photogrammetry-tutorial-11-how-to-handle-a-project-in-agisoft-photoscan/
8. Diettrich, J. Agisoft Photoscan Crash Course. 2015 June 24 [cited 2019 March 30]. Available from: https://adv-geo-research.blogspot.co.uk/2015/06/photoscan-crash-course-v1-1.html).
9. Davies TG, Rahman IA, Lautenschlager S, Cunningham JA, Asher RJ, Barrett PM, et al. Open data and digital morphology. Proc R Soc B. 2017;284: 20170194. doi: 10.1098/rspb.2017.0194.

10. Taylor KE. Summarizing multiple aspects of model performance in a single diagram. J. Geophys Res. 2001;106(D7): 7183-7192.

11. Marty D. Sedimentology, taphonomy, and ichnology of Late Jurassic dinosaur tracks from the Jura carbonate platform (Chevenez-Combe Ronde tracksite, NW Switzerland): insights into the tidal-flat palaeoenvironment and dinosaur diversity, locomotion, and palaeoecology. PhD. Thesis, l'Universite de Fribourg. 2008.

12. Whyte MA, Romano M, Elvidge DJ. Reconstruction of Middle Jurassic dinosaur-dominated communities from the vertebrate ichnofauna of the Cleveland Basin of Yorkshire, UK. Ichnos, 2007;14(1-2): 117-129. doi: 10.1080/10420940601010802.
